# Conservation and Targeted Wild Tending of *Schisandra chinensis* Under Global Change: Informed by SDMs and Fingerprint Analysis of Climate–Land–Composition Responses

**DOI:** 10.3390/biology15171501

**Published:** 2026-09-02

**Authors:** Detai Duan, Mengsha You, Mengjiao Li, Xinyi Li, Hengjun Xu, Boyan Zhang, Xinxin Zhang

**Affiliations:** Heilongjiang Research Center of Genuine Wild Medicinal Materials Germplasm Resources, School of Life Sciences and Technology, Harbin Normal University, Harbin 150025, China; 15083746885@163.com (D.D.);

**Keywords:** *Schisandra chinensis* (Turcz.) Baill., fingerprint, global change, conservation management, zoning for targeted wild tending

## Abstract

*Schisandra chinensis* (Turcz.) Baill. is a valuable medicinal plant species, and the demand for its wild resources is increasing continuously. However, the response mechanisms of its future habitat suitability and medicinal quality to global change remain poorly understood. This study integrated future climate projections under multiple Shared Socioeconomic Pathway (SSP) scenarios, land use dynamics, high-performance liquid chromatography (HPLC) fingerprint data, and an ensemble species distribution model to assess the habitat suitability, medicinal quality and conservation priorities of *S. chinensis*. Model performance evaluation indicated that the Biomod2 ensemble model achieves high prediction accuracy, with a True Skill Statistic (TSS) value of 0.882. Under the combined threat of climate change and land use change, the area of suitable habitat for *S. chinensis* in Heilongjiang Province will only account for 20.471% of the current suitable habitat area under the SSP5-8.5 scenario in the 2090s, and most suitable habitats will shift to the Greater Khingan Range region. The responses of different medicinal components of *S. chinensis* to future climate change vary considerably: for instance, the area of high-quality distribution for Schisandrol A and Gomisin A shows an overall expanding trend compared with the current status under future climate scenarios, while the area of high-quality distribution for Schizandrin B shows an overall shrinking trend. Within the planned conservation area for *S. chinensis* in Heilongjiang Province, the coverage of existing nature reserves is only 4.281%, and obvious conservation gaps exist in the Greater Khingan Range and Laoye Mountains regions. In response to the spatial distribution characteristics of the medicinal component content of *S. chinensis* under future climate change, we recommend implementing wild tending of *S. chinensis* in Tahe County and Huma County of the Greater Khingan Range region. The above findings provide a theoretical basis for the conservation and wild tending of *S. chinensis*.

## Simple Summary

*Schisandra chinensis* (Turcz.) Baill. is a valuable medicinal plant resource, and the demand for its wild resources has been continuously increasing in recent years. However, the response mechanism of its future habitat suitability and medicinal quality to global change remains poorly understood. This study comprehensively evaluated the suitable habitats, spatial distribution of medicinal quality, and conservation priority of *S. chinensis* by integrating future climate scenario simulation, dynamic prediction of land use, and high-performance liquid chromatography (HPLC) fingerprint data. The results showed that within the planned *S. chinensis* protected areas in Heilongjiang Province, the area actually covered by existing nature reserves only accounts for 4.281%, and significant conservation gaps exist in the Greater Khingan Range and Laoye Range. Based on the spatial distribution trend of the chemical component content of *S. chinensis* under future climate change, it is recommended that priority wild tending measures be implemented in Tahe County and Huma County in the Greater Khingan Range region. This study can provide a scientific basis for the conservation and targeted wild tending of *S. chinensis*.

## 1. Introduction

For thousands of years, plants have been the faithful partners on which humankind relies for survival, providing us with staple food to sustain ourselves and safeguarding our well-being [1]. It is estimated that 70% to 80% of the global population primarily relies on traditional herbal medicine to meet its basic healthcare needs [2]. As the population continues to grow and access to Western medicine (i.e., symptomatic treatment) remains generally limited in developing countries, demand for medicinal plants will continue to rise [3]. Some studies indicate that 70–90% of medicinal and aromatic plants are wild-harvested [4]. The growing demand for medicinal plants has placed enormous pressure on wild medicinal plant populations, and numerous species are currently at risk of extinction due to excessive overharvesting in wild habitats [5]. Therefore, it is crucial to protect the wild resources of medicinal plants and maintain a balance between their availability and medical demand. Currently, academia has proposed multiple strategies to address the shortage of wild medicinal plant resources, including in situ conservation (e.g., nature reserves and wild nurseries), ex situ conservation (e.g., botanical gardens and seed banks), and commercial cultivation [6,7,8,9]. Long-term production practices demonstrate that under the influence of specific ecological environments and anthropogenic factors, medicinal plants exhibit distinct regional characteristics [10,11]. Wild tending of Chinese medicinal materials, which relies on the natural habitats of medicinal plants supplemented with moderate human intervention, aims to maximize the restoration of the wild traits of medicinal materials. This approach effectively alleviates the contradiction between the shortage of wild resource supply and the continuous growth of market demand, and ensures the production of Chinese medicinal materials with authenticity and superior quality [12]. Accordingly, it is urgent to systematically elucidate the multi-scale interaction network between wild medicinal plants and various environmental factors, conduct in-depth analysis of their geospatial distribution patterns, and thereby provide an accurate theoretical framework for the scientific conservation and sustainable utilization of wild medicinal plant populations.

Climate change has been an inherent part of Earth’s evolutionary history and will continue to affect all aspects of plants in the future [13,14]. Furthermore, with the intensification of anthropogenic activities, climate change has become increasingly pronounced, and extreme weather events have also grown more frequent [15]. Driven by ongoing global climate change, biodiversity is experiencing an unprecedented decline this century [16]. Research findings indicate that climate change is projected to alter plant distribution patterns: species will expand into newly suitable habitats while declining in increasingly stressful environments [17]. Against this backdrop, how the distribution pattern of medicinal plants responds to climate change has become a key theoretical basis. It underpins the scientific conservation of their wild resources and the rational planning of planting layouts. Furthermore, the regional dependence of high-quality medicinal materials renders them particularly sensitive to climate change. Climate-driven distribution range migration not only threatens the survival of existing high-quality planting areas, but is also directly manifested as yield loss and quality alteration, which are the most intuitive signals of climate shocks. Most existing studies generally regard climate change as the sole dominant factor affecting biodiversity, while ignoring the key variable of land use change, and even fail to address the superposition effect generated by the interaction between the two factors [18]. Land use change may not only cause the loss of species habitats, but also trigger cascading effects such as the fragmentation of remaining habitats and the increase in agrochemical input into surrounding natural (or semi-natural) habitats [19,20]. Therefore, by incorporating global climate change and land use change into a unified analytical framework, we can achieve a more comprehensive assessment of the current and future potential distribution dynamics of medicinal plant populations. This approach can provide scientific knowledge support for reconciling the relationship between the conservation of wild medicinal plant resources and the demand for medicinal healthcare.

*Schisandra chinensis* (Turcz.) Baill. is a woody vine species belonging to the genus *Schisandra* of the family Schisandraceae. Its populations are mainly distributed in Northeast Asia, with the temperate forest vegetation region of China as its core distribution area [21]. *S. chinensis* Fructus is used as a medicine in the form of its dried ripe fruit. It exhibits the therapeutic effects of astringent and consolidating, enhancing qi while nourishing fluid, and tonifying the kidney and calming the mind. It demonstrates remarkable efficacy in the treatment of persistent cough with dyspnea due to deficiency, nocturnal emission and spermatorrhea, enuresis and frequent urination, persistent chronic diarrhea, spontaneous sweating and night sweating, impaired fluid with thirst due to yin deficiency, internal heat-induced consumptive thirst, palpitations and insomnia [22]. In natural habitats, *S. chinensis* seeds exhibit innate dormancy. The germination rate remains at a low level even after cold stratification treatment. This results in difficulties in sexual propagation of this species [23]. In addition, the differentiation ratio of female flowers is highly sensitive to changes in light conditions, and its instability directly triggers the common alternate bearing cycle in actual production, which has become a key bottleneck restricting the stable fruit yield of *S. chinensis* [24]. Given that *S. chinensis* struggles to achieve large-scale propagation via seeds in its natural habitat, and its wild resources have been excessively consumed, *S. chinensis* is listed as a Class Three protected plant in the National List of Rare and Endangered Medicinal Animal and Plant Species [25]. With the accelerated decline of wild *S. chinensis* resources driven by anthropogenic destruction and global change, the imbalance between the conservation and supply of *S. chinensis* resources has become increasingly prominent. Several critical limitations currently exist in species distribution modeling studies of *S. chinensis*: First, climate change is generally regarded as the sole driving factor. However, land use change and its potential interactive effects with climate change have been completely neglected. Second, existing studies are only limited to predicting the shift in population distribution ranges. They also fail to incorporate the spatial distribution pattern of the content of pharmaceutically active ingredients (such as lignans) into the analytical framework. As a result, it is impossible to assess the impact of environmental changes on the quality of *S. chinensis* medicinal materials [26,27,28,29,30].

To address the aforementioned research gaps, this study integrates multi-source data including environmental conditions, land use patterns and chemical compositions. It then innovatively couples ensemble species distribution models (SDMs), phytochemical prediction and conservation priority ranking, and develops a multi-dimensional analytical framework for the conservation and targeted wild tending of *S. chinensis* in Heilongjiang Province. Based on the above analytical framework, this study sets three core objectives as follows: first, to deeply analyze the functional mechanism of dominant environmental factors in regulating the population dynamics of *S. chinensis* and the accumulation process of its bioactive chemical components; second, to systematically elucidate the response pattern of the spatial distribution pattern of *S. chinensis* populations and the quality of its medicinal materials to climate change; third, based on future climate change and land use scenarios, to comprehensively determine the optimal spatial allocation scheme for the conservation of wild *S. chinensis* resources and targeted wild tending in Heilongjiang Province. 

## 2. Materials and Methods

### 2.1. Simulation of Current and Future Suitable Habitats for S. chinensis

#### 2.1.1. Acquisition of Species Distribution Points and Collection of Medicinal Materials of *S. chinensis*

**Figure 1:**
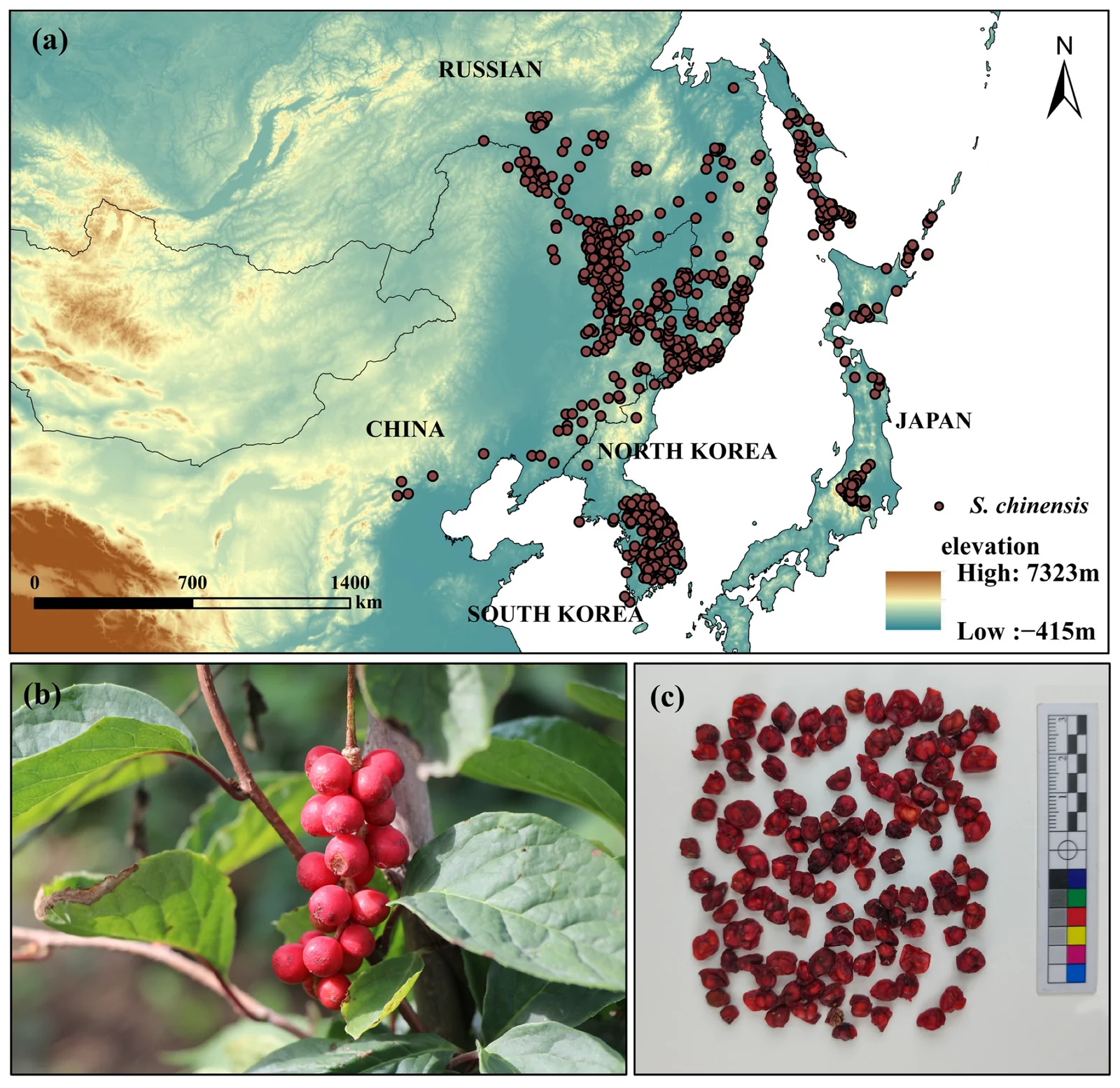
(**a**) Distribution point of *S. chinensis*; (**b**) Plant of *S. chinensis*; (**c**) Medicinal materials of *S. chinensis* (dried fruits).

A total of 2131 occurrence records of *S. chinensis* from 2000 to the present were obtained through field surveys across Heilongjiang Province and global data retrieval from multiple online platforms, including the National Specimen Information Infrastructure (http://www.nsii.org.cn/, accessed on 20 September 2025), the Global Biodiversity Information Facility (GBIF.org, (https://doi.org/10.48580/dgykv, accessed on 20 September 2025)), and the Chinese Virtual Herbarium (http://www.cvh.ac.cn/, accessed on 20 September 2025). Given that species identification is fundamental to species conservation, we verified specimens sourced from global datasets, and eliminated records with erroneous identifications as well as those from non-suitable habitats, such as highways and residential areas [31]. Subsequently, we employed ENMtools to retain only one occurrence point within a 5 km grid. Finally, 727 occurrence points were retained for model construction (Figure 1). A total of 46 batches of *S. chinensis* medicinal materials (mature fruits of *S. chinensis*) were collected from various districts and counties in Heilongjiang Province. The collection was concentrated in the fructescence of *S. chinensis* fruits in each region. Specific collection time and sampling locations are shown in Supporting Information of The Chromatographic Fingerprint. After collection, impurities were removed and the materials were uniformly sun-dried. The identification of all the above data points was assisted and processed by Professor Wang Chen from Harbin Normal University.

#### 2.1.2. Acquisition of Environmental Variables and Geographic Data

In species distribution modeling, factors closely associated with plant physiology and ecological responses should be prioritized to effectively guarantee the reliability of prediction results [32]. Accordingly, this study comprehensively considered the ecological characteristics of *S. chinensis*; that is, *S. chinensis* favors sunlight and moisture, and has specific requirements for temperature and soil conditions. A total of 35 environmental variables (including climate, solar radiation, and soil physical and chemical properties—see Table S1) were incorporated in this study to simulate the distribution range of this species [29,33,34]. Nineteen climatic factors from 2000 to 2018 (e.g., mean diurnal temperature range, temperature seasonality, precipitation of the driest quarter, etc.) were downloaded as climatic variables from the WorldClim database (v2.1) (www.worldclim.org, accessed on 20 September 2025). Solar radiation (Srad) data covering 1970–2000 was also obtained from the WorldClim database (v2.1), which was processed via the raster calculator in ArcMap 10.5 to generate solar radiation values for four seasons, to better align with the growth and development of *S. chinensis*. Since the root system of *S. chinensis* is distributed in shallow soil layers and its growth is affected by soil organic matter, pH and soil, 12 shallow soil variables including calcium carbonate or lime content (CaCO_3_) and pH of topsoil (0–30 cm) were downloaded from the Harmonized World Soil Database (HWSD). All environmental variables have a spatial resolution of 2.5 arc-minutes.

Subsequently, we incorporated these environmental variables and species distribution coordinates into the Maximum Entropy (MaxEnt) model, which can accurately identify important variables by generating a relative contribution table [35]. Based on Table S2, we discarded environmental variables with a contribution of less than 0. Next, we performed Pearson’s rank correlation analysis on the remaining environmental variables using the ENMtools v 1.0.4 software [36], and retained the environmental variables with high contribution and low correlation coefficients (r, |r| < 0.8), as shown in Table S3. Based on the Pearson correlation analysis, this study further calculated the variance inflation factor (VIF) of each environmental factor to eliminate the interference of potential multicollinearity among environmental factors on model estimation [37]. With VIF < 10 set as the tolerance threshold for multicollinearity, the results show that the VIF values of all factors range from 1.1 to 6.2 (see Table 1), which are far below the critical value. This indicates that there is no severe multicollinearity among the selected environmental factors, and all factors can be included simultaneously in subsequent analyses. Finally, 17 environmental variables were retained (Table 1).

To evaluate the impact of climate change on the potential suitable distribution area of *S. chinensis* and reduce prediction uncertainty caused by a single general circulation model, this study selected three widely adopted general circulation models (CNRM-CM6-1 (Centre National de Recherches Météorologiques—Climate Model Version 6), MIROC-ES2L (Model for Interdisciplinary Research on Climate, Earth System version 2 for Long-term simulations) and MRI-ESM2-0 (Meteorological Research Institute Earth System Model version 2.0)) from the WorldClim climate database, and applied their equally weighted averaged outputs to subsequent analyses. The selected models are commonly used in species distribution modeling and exhibit high applicability [38,39,40]. Each of the aforementioned general circulation models incorporates four Shared Socioeconomic Pathways (SSP1-2.6 (Shared Socioeconomic Pathway 1-2.6 (Sustainability)), SSP2-4.5 (Shared Socioeconomic Pathway 2-4.5 (Middle of the Road)), SSP3-7.0 (Shared Socioeconomic Pathway 3-7.0 (Regional Rivalry)) and SSP5-8.5 (Shared Socioeconomic Pathway 5-8.5 (Fossil-fueled Development))), and for each pathway, four time periods are extracted: 2021–2040 (2030s), 2041–2060 (2050s), 2061–2080 (2070s), and 2081–2100 (2090s). Due to the absence of available corresponding future data for solar radiation and soil properties, this study treats solar radiation and soil factors as static variables in species distribution modeling. The spatial resolution of all the aforementioned environmental variables is 2.5 arc-minutes.

To elucidate the potential impacts of land use change on the habitat suitability of *S. chinensis*, this study integrated land use data when constructing suitable habitat. The current (2020) land use data were obtained from the MODIS dataset provided by the National Aeronautics and Space Administration (NASA) Earth Data Platform (https://search.earthdata.nasa.gov/search/, accessed on 20 September 2025); the future land use data were sourced from the research findings of Hou et al. (https://doi.org/10.6084/m9.figshare.20088368.v1, accessed on 20 September 2025), and four sets of scenario data corresponding to different climate scenarios (SSP1-2.6, SSP2-4.5, SSP3-7.0, SSP5-8.5) were selected. All land use data were resampled to a resolution of 2.5 arc-minutes using ArcMap 10.5 software.

Maps of China’s administrative divisions and nature reserves were obtained from the National Geomatics Center of China (https://www.webmap.cn/, accessed on 20 September 2025). MaxEnt version 3.4.4 was downloaded from the following website: http://biodiversityinformatics.amnh.org/open_source/maxent/, accessed on 20 September 2025. This study employed ArcGIS 10.5 and RStudio 4.0.4 for data analysis and processing.

#### 2.1.3. Simulation of Suitable Habitats for *S. chinensis* Under Current and Future Climate Scenarios

Biomod2 R Package (v3.5.1) in R 4.0.4 was used to simulate the potential suitable distribution areas of *S. chinensis* under climate change. Biomod2 is an integrated R package specifically designed for species distribution modeling, which supports multiple algorithms and their integrated ensembles [41]. In this study, ten modeling algorithms, namely GLM (General Linear Model), GBM (Gradient Boosting Machine), GAM (Generalized Additive Model), CTA (Classification and Regression Tree Analysis), ANN (Artificial Neural Network), SRE (Site Reliability Engineering), FD (Functional Data), MARS (Multivariate Adaptive Regression Splines), RF (Random Forest) and MaxEnt (Maximum Entropy Model), are employed and have been proven to have good predictive performance [41,42]. Research findings indicate that the prediction performance of the MaxEnt model is closely correlated with feature combination (FC) and regularization multiplier (RM). The MaxEnt model constructed with default parameters is prone to overfitting, which results in low transferability of the model, and the derived prediction results are not optimal and may even be unreliable [43,44]. Accordingly, we tuned the feature combination (FC) and regularization multiplier (RM) parameters of the model using the Kuenm package to obtain the parameter combination with the optimal goodness of fit for the input data. First, we constructed a large set of candidate models, then evaluated and selected the optimal model. All candidate models were generated by combining 17 regularization multiplier values (0.1 to 1 at 0.1 intervals, 2 to 6 at 1 intervals, plus 8 and 10) with all 29 possible feature class combinations (linear-L, quadratic-Q, hinge-H, product-P and threshold-T). Second, candidate models were screened by measuring statistical significance with the partial ROC (Receiver Operating Characteristic) value, assessing model prediction capability with the omission rate, and evaluating model complexity with the AICc (Akaike Information Criterion, corrected) value. The model parameter combination with the lowest AICc value (ΔAICc = 0) was selected as the optimal model [45]. After optimization, the parameters of MaxEnt model were set as RM = 0.2 and FC = LQP.

To improve prediction accuracy, 75% of the occurrence records were designated as training data, and the remaining portion was used as the final evaluation dataset for the model. The segmentation of training data and test data was repeated three times. For the configuration of pseudo-absence points, we select four candidate size gradients of 727 (consistent with the number of real existing points), 1000, 5000, and 10,000, and calculate the average True Skill Statistic (TSS) value across ten candidate models (see Table S4). The results show that when the number of pseudo-absence points is 5000, the average TSS of all models reaches the maximum, so this parameter is uniformly adopted for subsequent calculations [46,47]. The value of TSS ranges from −1 to 1. The closer this indicator is to 1, the better the simulation performance of the model [48,49]. The mean TSS value in this study is 0.882, indicating that our models achieve relatively high accuracy. For simulation studies, it is generally agreed that models with a TSS value greater than 0.8 exhibit excellent predictive performance; therefore, we only retain models with TSS scores greater than 0.8 for ensemble modeling [50,51]. In this study, we first used the Biomod2 package to simulate the suitable distribution of *S. chinensis* under current climate scenarios, and output continuous suitability raster layers (where each grid corresponds to a suitability probability value) and binary threshold layers (where 0 and 1 denote unsuitable and suitable areas, respectively). Next, we performed reclassification processing on the threshold layers in ArcMap 10.5, extracted the raster data of suitable habitats, and superimposed the continuous suitability raster layer using the extracted raster layer as a mask. Finally, we calculated the optimal threshold for distinguishing suitable areas from unsuitable areas, which is 0.471. Specifically, areas with a species occurrence probability equal to or greater than P (*p* ≥ P) are defined as suitable areas, while the remaining areas are classified as non-suitable areas. The study area was further divided into four categories: regions with *p* < 0.471 were classified as unsuitable areas; regions with 0.471 ≤ *p* < 0.55 were classified as low-suitability areas; regions with 0.55 ≤ *p* < 0.75 were classified as moderate-suitability areas; regions with 0.75 ≤ *p* < 0.986 were classified as high-suitability areas.

The above analysis only identifies climatically suitable areas for *S. chinensis*, and has not yet excluded areas unfavorable for the growth of *S. chinensis* such as water areas and cultivated land. Therefore, when formulating the planning for suitable habitats of *S. chinensis*, this study further integrates current and future land use data, and conducts spatial overlay between climatically suitable areas and natural forests (i.e., natural habitat types suitable for the growth of *S. chinensis*). The overlapping area obtained is defined as the suitable habitat of *S. chinensis*.

### 2.2. Correlation Analysis Between Environmental Variables and the Chemical Constituents of S. chinensis

To facilitate the application of research findings to practical production, it is necessary to identify which environmental factors may induce changes in the content of chemical constituents in medicinal plants. Accordingly, this study established a correlation analysis between environmental factors and active constituents.

#### 2.2.1. Ranking of the Importance of Chemical Component Determination of *S. chinensis* in Heilongjiang Province and Related Environmental Factors

In this study, high-performance liquid chromatography was employed to determine 46 batches of wild *S. chinensis* samples collected across Heilongjiang Province, and the data were analyzed using the Similarity Evaluation System for Chromatographic Fingerprint of Traditional Chinese Medicine. For detailed methodological procedures, please refer to the Supporting Information of the chromatographic fingerprint. It is generally recognized that the determination of any single active component or index constituent cannot fully characterize the intrinsic quality of traditional Chinese medicine [52]. Principal Component Analysis (PCA) can extract several independent composite variables based on practical requirements, with the aim of retaining the maximum amount of information from the original variables. This method constitutes a scientifically valid dimensionality reduction technique [53]. Principal Component Analysis (PCA) was performed on the normalized peak areas of 9 common peaks from 46 samples using the R v4.1.2 software package. The eigenvalues, contribution rates and cumulative contribution rates were calculated, and the screening plot (Figure S1) was generated. To clarify the extent to which environmental variables influence the chemical constituents of *S. chinensis*, this study is based on the XGBoost-SHAP (eXtreme Gradient Boosting-SHapley Additive exPlanations analysis. Taking the response variable FD (peak area of each chemical component) as the stratification basis, the training set is extracted from the entire sample at a ratio of 70%, and model fitting is performed [54]. Model fitting adopts a pre-defined hyperparameter configuration, and the objective function is set as mean squared error. On the training set, by comparing the model prediction results with the measured values, the root mean squared error (RMSE), mean absolute error (MAE) and coefficient of determination (R^2^) are calculated to evaluate the in-sample fitting performance of the model. Furthermore, to analyze the influence mechanism of various environmental factors on FD, the SHAP value of each feature was calculated sample-by-sample by combining the TreeExplainer algorithm based on the trained model. Based on the mean absolute SHAP value corresponding to each feature, the global importance of environmental factors is obtained through sorting [55] (Table S5). To eliminate the interference from correlations among environmental variables on the subsequent calculation of the model, Pearson’s rank correlation analysis implemented in ENMTools v 1.0.4 was performed to retain environmental variables with high feature importance and low correlation coefficients (|r| < 0.8). The environmental factors retained for modeling in each component are shown in Table S6.

#### 2.2.2. Construction of the Spatial Distribution Pattern of Chemical Constituents of *S. chinensis* in Heilongjiang Province

All-subsets regression analysis traverses all possible models and screens out the optimal model based on statistical criteria such as adjusted R^2^ and Mallows Cp. On this basis, the relative weight analysis method can be further applied to quantify the contribution degree of each variable to the explanatory power of the model [56]. Based on the sampling sites of *S. chinensis* in Heilongjiang Province and the calculation results of fingerprints, the environmental factor data corresponding to the coordinates of 46 samples and the peak area data of fingerprints were input into RStudio (2023.06.1+524). Using the MuMIn v 1.47.5 package, with corresponding environmental factors as independent variables (x), the comprehensive principal component (pc) with significant correlation with environmental variables and the peak areas of five chemical components (Schisandrol A, Gomisin A, Angeloylgomisin H, Schisantherin B, Schizandrin A and Schizandrin B) were taken as dependent variables (y) respectively, to construct the correlation between the chemical components of *S. chinensis* and environmental factors. The specific formula is as follows:

y = *β*_1_x_1_ + *β*_2_x_2_ + … + *β*_i_x_n_ (1)

where y represents the content of chemical components, x_n_ denotes environmental variables, and *β*_i_ are regression coefficients, with results evaluated using the coefficient of determination (R^2^) and F-test [57]. Based on these results, the comprehensive principal component (pc) of *S. chinensis*’s comprehensive components and the spatial distribution of individual components were constructed using ArcGIS 10.5.

### 2.3. Planning of S. chinensis Protected Areas and Targeted Wild Tending in Heilongjiang Province

#### 2.3.1. Protected Areas Planning

In protected area planning, to obtain climatically relatively stable suitable habitats for *S. chinensis* throughout the 21st century, we conducted spatial overlay of suitable distribution areas under 17 current and future climate scenarios, took the overlapping area as a template, uniformly masked the suitable habitat layers of all scenarios to the extent of this template, and finally imported the processed data into the Zonation 4.0 software platform to perform priority area ranking calculation. All layers are uniformly assigned a weight of 1, and the remaining parameters retain the model’s default settings. By setting the warp factor to “1” and adopting the cell-based removal strategy, the conservation planning results are further optimized [58]. Existing research shows that protecting 5–20% of habitats can enable the conservation of over 50% of species [59]. For practical considerations, the top 20% of ranked areas are delineated as protected reserves in accordance with the set objectives, and existing conservation gaps are identified synchronously. The output results are ultimately classified as follows: the top 5% is delineated as priority protection areas, 5% to 10% as general protection areas, and 10% to 20% as partial protection areas [60].

#### 2.3.2. Targeted Wild Tending Planning

Wild tending of traditional Chinese medicinal materials is an ecological cultivation approach that closely mimics natural conditions. Rooted in the original habitat of the targeted species, this approach fully adheres to the biological laws of traditional Chinese medicinal materials on the premise of maintaining ecosystem stability. It primarily relies on natural ecological processes with minimal artificial intervention, thereby achieving the sustainable enhancement of the population productivity of geo-authentic crude drugs [6]. The core scientific objective of wild tending is to accurately quantify and elucidate the formation mechanism of the geo-authenticity of medicinal materials, thereby providing solid theoretical and data support for site selection decision-making of tending bases [61]. Meanwhile, Huang et al. proposed the concept of directional cultivation of Chinese medicinal materials, and highlighted that the selection of cultivation regions for Chinese medicinal materials is one of the most critical factors affecting the quality and yield of Chinese medicinal materials, and the selection of geo-authentic production regions is the most fundamental and critical principle for the cultivation of geo-authentic Chinese medicinal materials [62].

Based on the above analysis, the targeted wild tending area planned in this study aims to identify high-quality districts and counties for each chemical component of *S. chinensis* in Heilongjiang Province under various climate scenarios. Both the habitat suitability and medicinal quality of *S. chinensis* are taken into consideration for delineating this area. Accordingly, taking the suitable habitats of *S. chinensis* under each climate scenario as a mask, we conducted masking processing on the spatial distribution layers of each component content obtained in Section 2.2 respectively, and then input both datasets into Zonation for calculation. The parameter settings are completely consistent with those adopted in the priority protected area planning. Given that development and construction activities are prohibited within nature reserves, both the established nature reserves in Heilongjiang Province and the protected areas planned in this study are excluded from the output results of the Zonation software. The natural break method is based on the inherent natural grouping characteristics of data, realizes the optimal aggregation of homogeneous values by identifying classification intervals, and maximizes the differences between different categories [63]. Therefore, this study adopts the natural break method to divide the results into high-quality regions and general-quality regions. In China, local governments act as major participants in biodiversity conservation actions; accordingly, we conducted a county-scale hotspot analysis on the high-quality regions of wild *S. chinensis* populations in Heilongjiang Province, and finally obtained the hotspot districts and counties for targeted wild tending of each component of *S. chinensis* in Heilongjiang Province [64].

## 3. Results

### 3.1. Key Environmental Factors Driving S. chinensis Distribution and the Pattern of Its Potential Suitable Areas Under Current Climate Scenarios

Based on the calculation results from Biomod2, environmental factors with an average importance of >15% in model evaluation are defined as the primary environmental factors affecting the growth of *S. chinensis*. Ranked from highest to lowest importance, these factors are srad_winter, bio18, srad_summer, and bio13. The importance assessment of environmental factors used for modeling across each model is presented in Figure S2. By extracting 5000 random points from the current suitable habitat distribution for the four primary environmental variables, we obtained the following value ranges: srad_winter (Solar radiation from DEC to FEB) spans 4374–10,109 kJ·m^−2^·day^−1^, bio18 (Precipitation of warmest quarter) spans 218.819–1032.41 mm, srad_summer (Solar radiation from JUN to AUG) spans 14,746–19,921 kJ·m^−2^·day^−1^, and bio13 (Precipitation of wettest month) spans 118.458–611.194 mm (Table S7).

**Figure 2:**
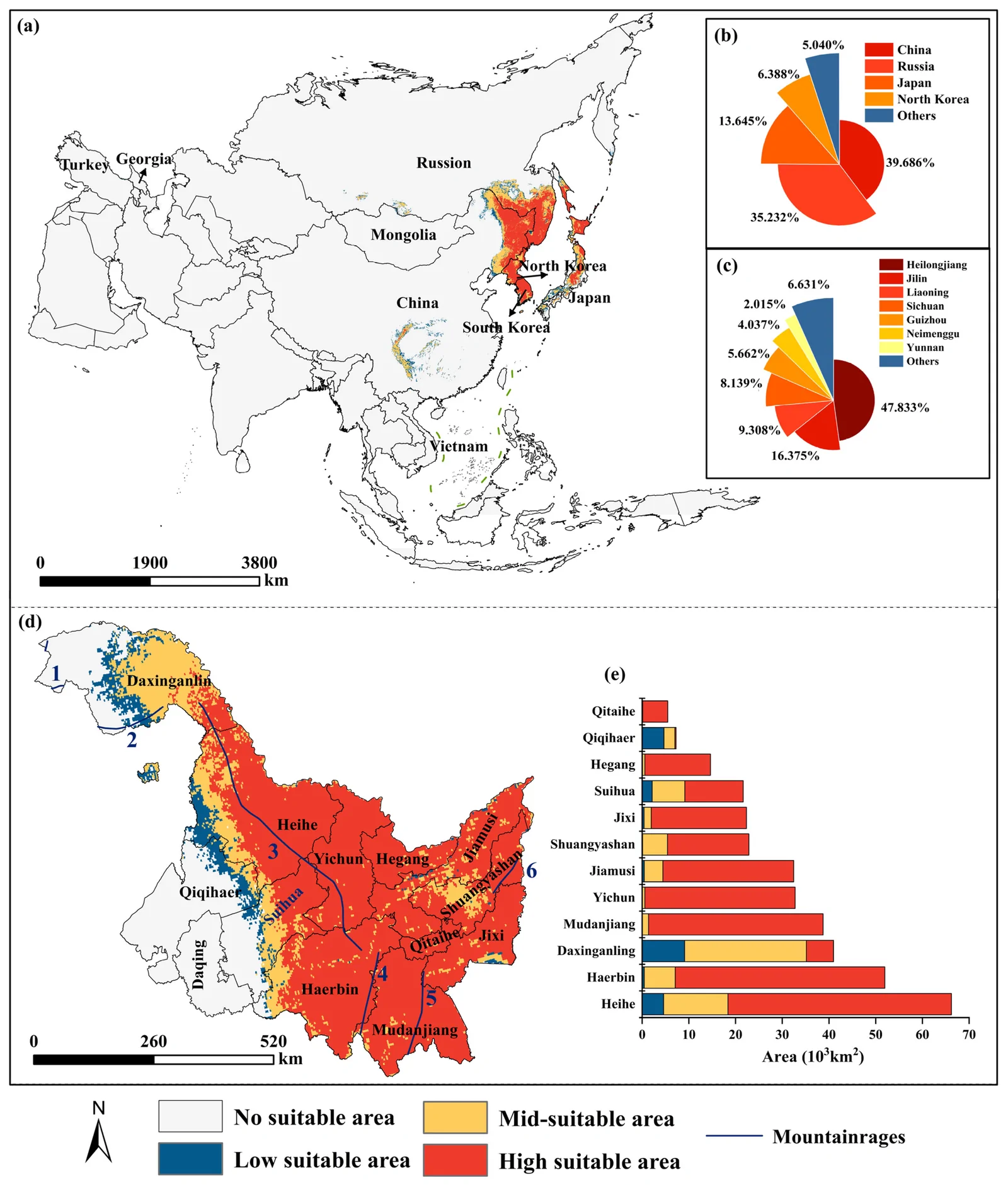
(**a**) Distribution of the current suitable areas for *S. chinensis* across the entire ecological niche in Asia. (**b**) Proportion of current suitable areas in each country relative to the total suitable areas, where other countries include the Republic of Korea, Mongolia, Vietnam, Turkey and Georgia. (**c**) Proportion of current suitable areas in each province of China relative to the total current suitable areas of China, where other provinces include Hebei Province, Shanxi Province, Gansu Province, Chongqing Municipality, Guangxi Zhuang Autonomous Region, Hunan Province, Henan Province, Guangdong Province, Anhui Province, Jiangxi Province, Zhejiang Province and Fujian Province. (**d**) Distribution of current suitable areas for *S. chinensis* in Heilongjiang Province (1: The Greater Khingan Range; 2: Yilehuli Mountain; 3: The Lesser Khingan Range; 4: Zhangguangcai Range; 5: Laoye Range; 6: Wanda Mountain). (**e**) Area distribution of current low-, medium- and high-suitability areas for *S. chinensis* across all cities in Heilongjiang Province.

As shown in Figure 2, the simulation results obtained via Biomod2 indicate that the total suitable area of *S. chinensis* is 18.780 × 10^5^ km^2^. Among the three suitability grades, the highly suitable habitat has the largest area at 9.947 × 10^5^ km^2^, accounting for 52.964% of the total suitable habitat area; it is followed by the moderately suitable habitat, with an area of 6.233 × 10^5^ km^2^, accounting for 33.188% of the total suitable habitat area; the lowly suitable habitat has the smallest area of 2.601 × 10^5^ km^2^, accounting for 13.848% of the total suitable habitat area, as shown in Figure S3a. Within the entire ecological niche (Asia) of *S. chinensis*, the suitable habitat area in China is the largest at 7.453 × 10^5^ km^2^, accounting for 39.686% of the total suitable habitat area, as shown in Figure S3b.

Within the territory of China, the suitable distribution areas of *S. chinensis* are concentrated in Northeast China, including Heilongjiang Province, Jilin Province, Liaoning Province and eastern Inner Mongolia. Among these regions, Heilongjiang Province has the largest total suitable area for *S. chinensis*, which is 3.562 × 10^5^ km^2^, accounting for 47.833% of the total suitable area in China, as shown in Figure 2c. The area of highly suitable habitats for *S. chinensis* in Heilongjiang Province is 2.660 × 10^5^ km^2^, the area of moderately suitable habitats is 0.687 × 10^5^ km^2^, and the area of lowly suitable habitats is 0.215 × 10^5^ km^2^. The suitable distribution range of *S. chinensis* in Heilongjiang Province is relatively broad. Except for the western grasslands and the northernmost regions, all other areas are suitable for its distribution, and the highly suitable habitats are concentrated in mountainous areas, as shown in Figure 2d,e.

### 3.2. Key Environmental Factors Driving the Distribution of S. chinensis and Patterns of Its Potential Suitable Areas in Heilongjiang Province Under Future Climate Scenarios

Under future climate scenarios, among the four key environmental factors driving the distribution of *S. chinensis*, bio13 and bio18 will vary with carbon emission scenarios and the passage of time. Compared with current conditions, the average value of bio13 (precipitation of the wettest month) decreases across all future climate scenarios. Among different carbon emission scenarios, all but the SSP1-2.6 climate scenario show an increasing trend over time; the SSP1-2.6 scenario exhibits a trend of increasing first, followed by decreasing, then increasing again. Compared with current conditions, the average value of bio18 (precipitation of the warmest quarter) increases across all future climate scenarios. Among different carbon emission scenarios, all but the SSP1-2.6 climate scenario show a continuous increasing trend over time, while the SSP1-2.6 scenario presents a trend of increasing first and decreasing afterward (Figure S4).

**Figure 3:**
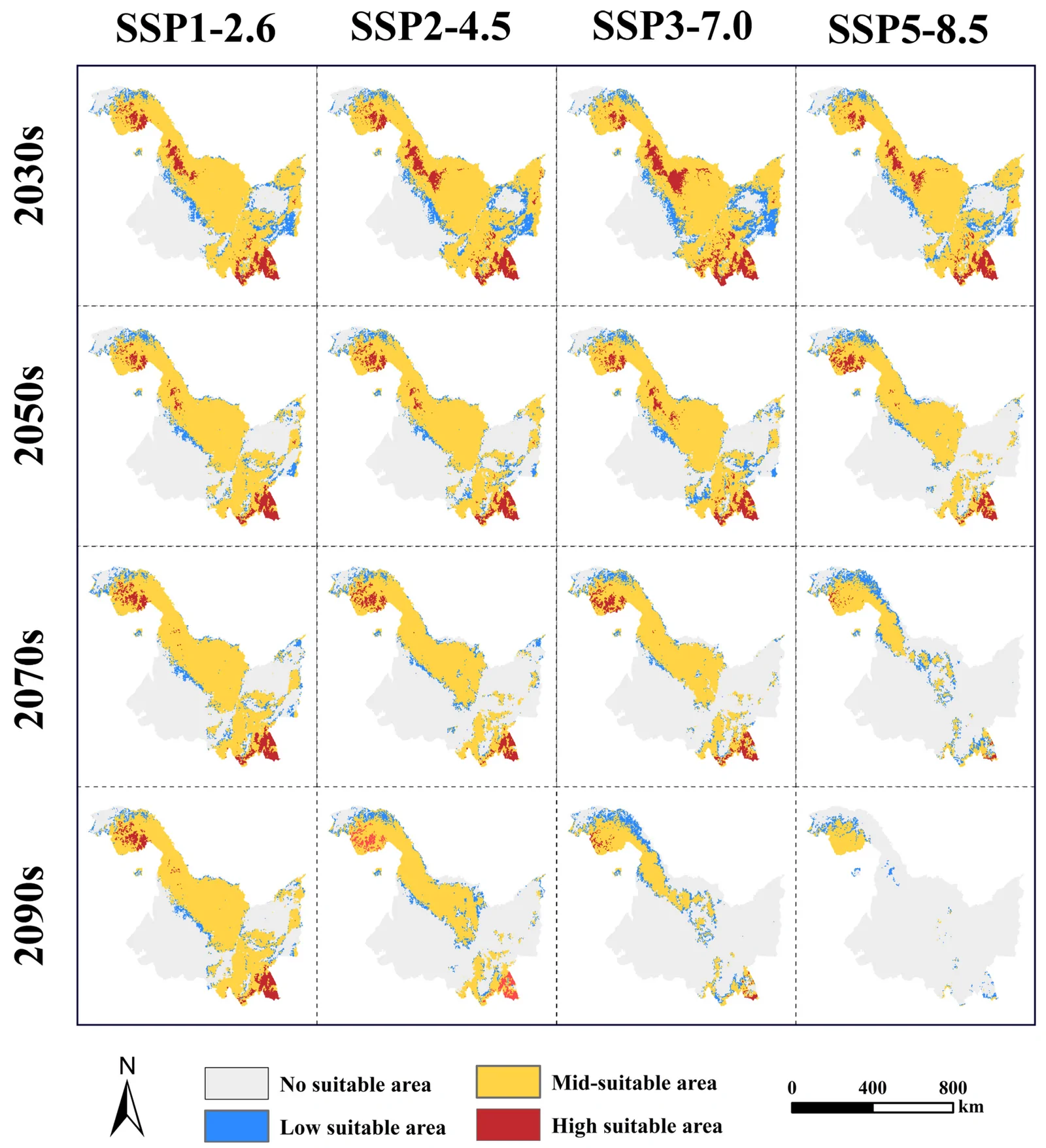
Changes in the distribution pattern of suitable growth areas of *S. chinensis* in Heilongjiang Province under the background of future global climate change.

**Figure 4:**
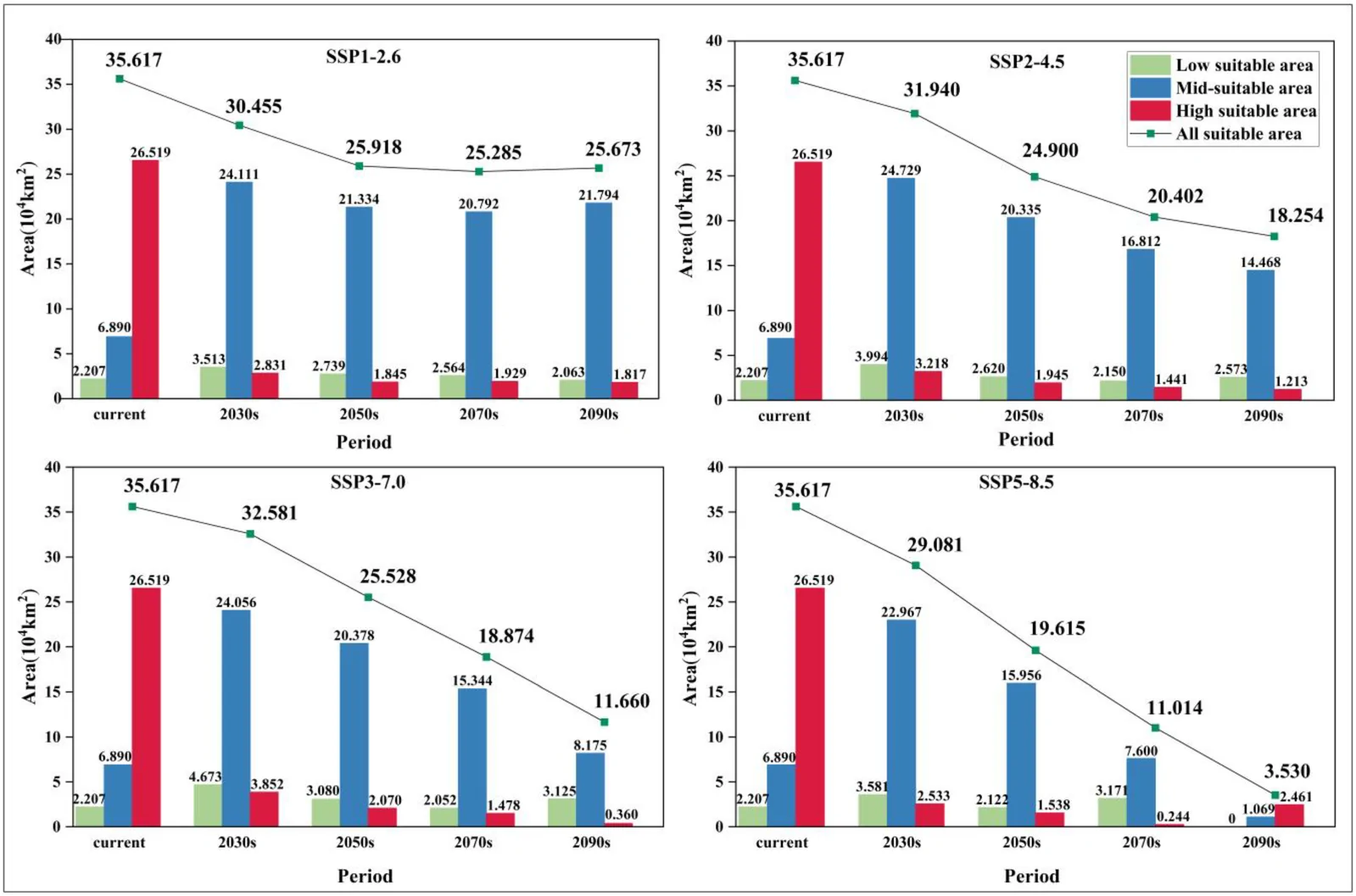
Changes in the area of suitable areas for *S. chinensis* at each suitability level in Heilongjiang Province under future global climate change scenarios.

Under future climate change scenarios, the total area of suitable areas for *S. chinensis* in Heilongjiang Province will decrease year by year toward the northwest with the increase in years and carbon emissions (Figure 3). It is projected that under the SSP5-8.5 climate scenario for the 2090s, the area of suitable habitats for *S. chinensis* will shrink to the minimum, approximately 3.530 × 10^4^ km^2^, accounting for only 9.912% of the current area of suitable areas. Moreover, the low-suitability areas for *S. chinensis* will disappear completely under this scenario. Except that the current suitable areas are dominated by high-suitability areas, the pattern of suitable areas under various future climate scenarios will shift to being dominated by medium-suitability areas (Figure 4).

### 3.3. Dynamic Prediction of the Area of Suitable Habitats for S. chinensis in Heilongjiang Province Under Different Climate Scenarios

**Figure 5:**
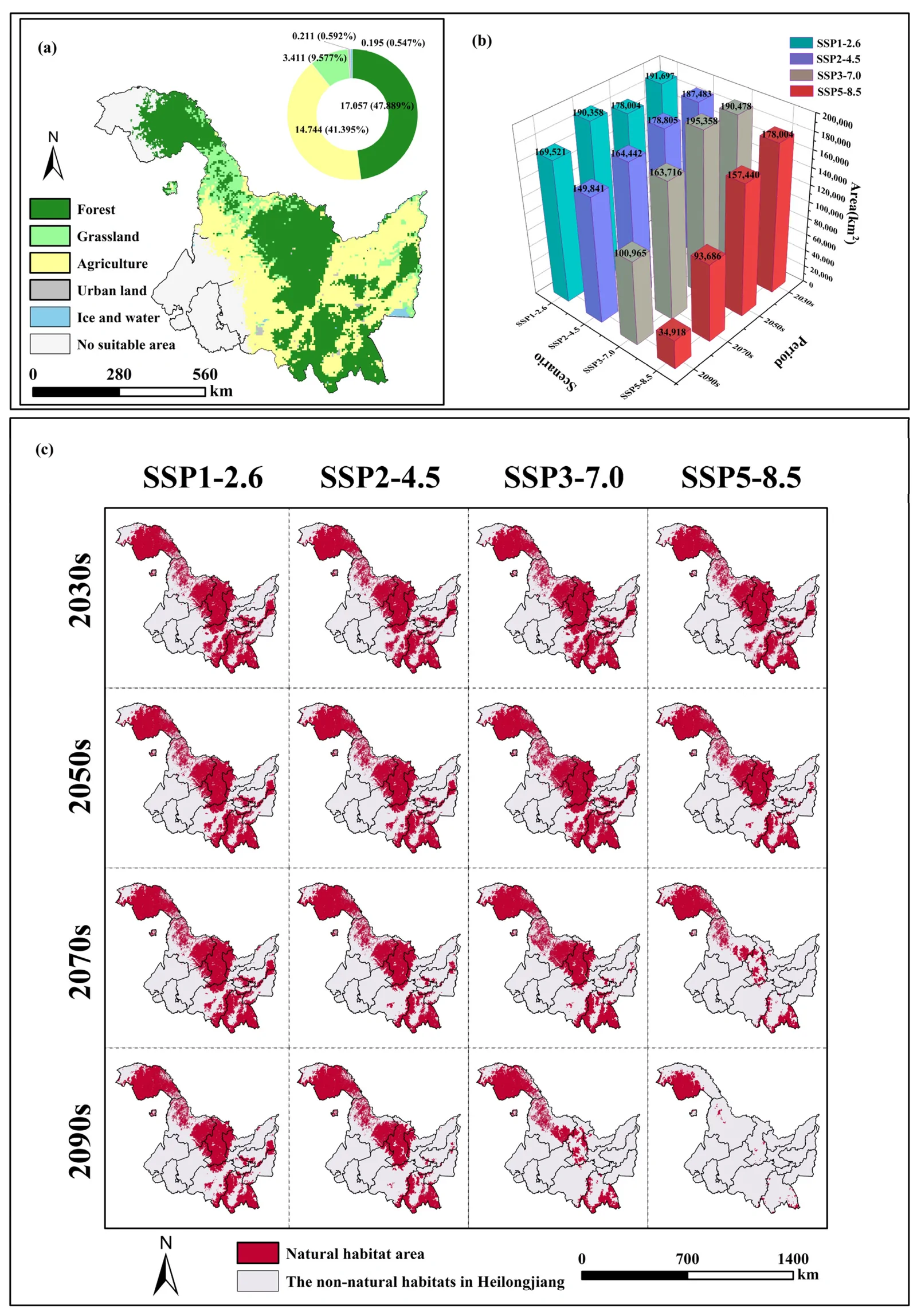
(**a**) The current land use situation within the suitable habitat area of *S. chinensis* in Heilongjiang Province. (**b**) The changes in the suitable habitat area of *S. chinensis* in Heilongjiang Province under the influence of global change. (**c**) Spatial pattern of suitable habitats for *S. chinensis* in Heilongjiang Province under global change.

Forest land constitutes the primary habitat for *S. chinensis* under natural conditions. Under the current climate scenario, the forest land area within the suitable distribution range of *S. chinensis* in Heilongjiang Province is approximately 1.706 × 10^5^ km^2^, accounting for 47.891% of the total suitable distribution area (Figure 5a). In this study, the woodland located within the suitable distribution range is defined as the suitable habitat of *S. chinensis*. Based on the distribution of suitable distribution range of *S. chinensis* and the changes in woodland area in Heilongjiang Province under future climate scenarios, the future suitable habitat of *S. chinensis* in Heilongjiang Province can be obtained. Under the SSP1-2.6 and SSP2-4.5 carbon emission scenarios, the area of suitable habitats for *S. chinensis* in Heilongjiang Province will remain basically stable from the 2030s to the 2090s compared with that under the current climate scenario. However, under the SSP3-7.0 carbon emission scenario, the area of suitable habitats for *S. chinensis* in Heilongjiang Province shows a significant trend of increasing first and then decreasing. Under the SSP5-8.5 carbon emission scenario, the area of suitable habitats presents a gradual decreasing trend with the increase in years. It is projected that under the SSP5-8.5 climate scenario in the 2090s, only 3.492 × 10^4^ km^2^ of suitable habitats for *S. chinensis* will remain within Heilongjiang Province, accounting for only 20.471% of the current area of suitable habitats for *S. chinensis* in Heilongjiang Province (Figure 5b,c) (Table S8).

### 3.4. Establishment of HPLC Fingerprint of S. chinensis Medicinal Material, Identification of Characteristic Peaks and Similarity Evaluation of 46 Batches of Samples

**Figure 6:**
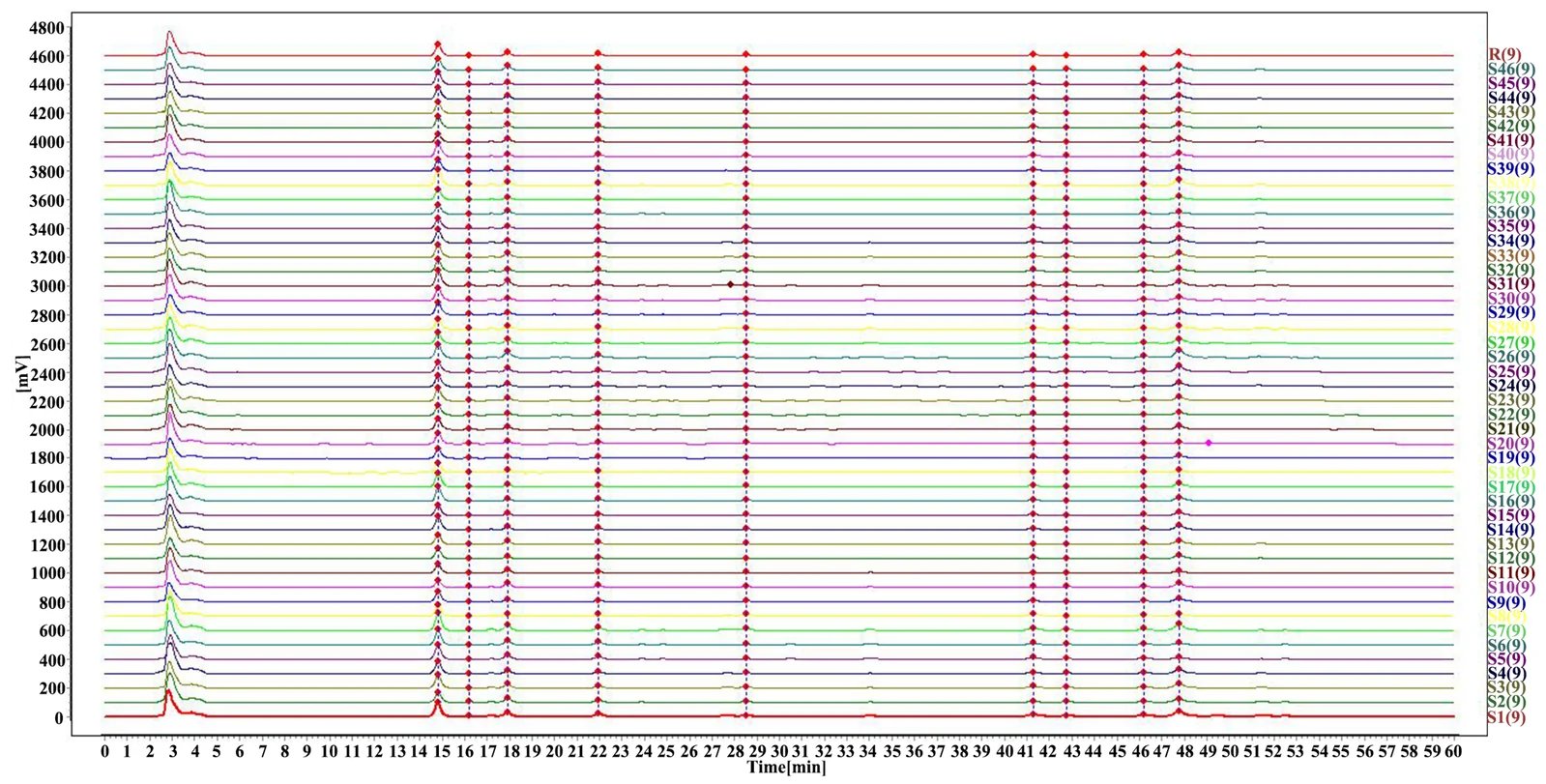
Fingerprint patterns of 46 batches of *S. chinensis* and the control group(s) (The red dots indicate the apex positions of common peaks identified based on the reference spectrum.).

The Similarity Evaluation System for Chromatographic Fingerprint of Traditional Chinese Medicine (Version 2012) was adopted. Taking the chromatogram of the reference substance as the reference, the control reference fingerprint was generated by fitting the chromatograms of 15 batches of test samples (see Supporting Information of The Chromatographic Fingerprint). The fitting result is shown in Supporting Information of The Chromatographic Fingerprint. After fitting, a total of 18 common peaks were obtained. According to the chromatographic profiles of each sample, in addition to six components (Schisandrol A, Gomisin A, Angeloylgomisin H, Schizandrin B, Schisantherin B, Schizandrin A) whose chromatographic peaks have been located with reference standards, another three chromatographic peaks with good resolution, large peak area and stable retention were selected as characteristic peaks, and identification was performed by calculating the relative retention time of each characteristic peak with respect to the peak of Schisandrol A. A total of nine characteristic peaks are presented in Supporting Information of The Chromatographic Fingerprint. The baseline of the chromatogram is stable, with a large number of peaks, favorable response performance, good peak symmetry, and high resolution. Based on the optimal experimental conditions obtained in the Supporting Information of the fingerprint, the fingerprint construction was performed for 46 batches of *S. chinensis*, and the result is shown in Figure 6. The similarity evaluation results are presented in Figure S5. The similarity between the 46 batches of *S. chinensis* samples and the reference chromatogram ranged from 0.838 to 1. The similarity values for *S. chinensis* from Tangyuan County, Bayan County, and Wuchang City were only 0.838 and 0.840, indicating relatively low similarity, whereas those from Youhao District, Jinlin District, Suifenhe City, Hailin City, Sunwu County, Bei’an City, and Aihui District reached 1, showing extremely high similarity. These results indicate that the quality of *S. chinensis* medicinal materials varies among different regions.

### 3.5. Spatial Prediction Analysis of Chemical Constituents in S. chinensis from Heilongjiang Province

After XGBoost-SHAP analysis, the importance ranking of 34 environmental factors meeting the criteria was obtained. After removing environmental factors with a correlation ≥ 0.80 between any two factors and that had lower importance, the significant environmental factors associated with the comprehensive principal component (pc) of *S. chinensis* constituents and each individual constituent were identified (Table S6).

**Figure 7:**
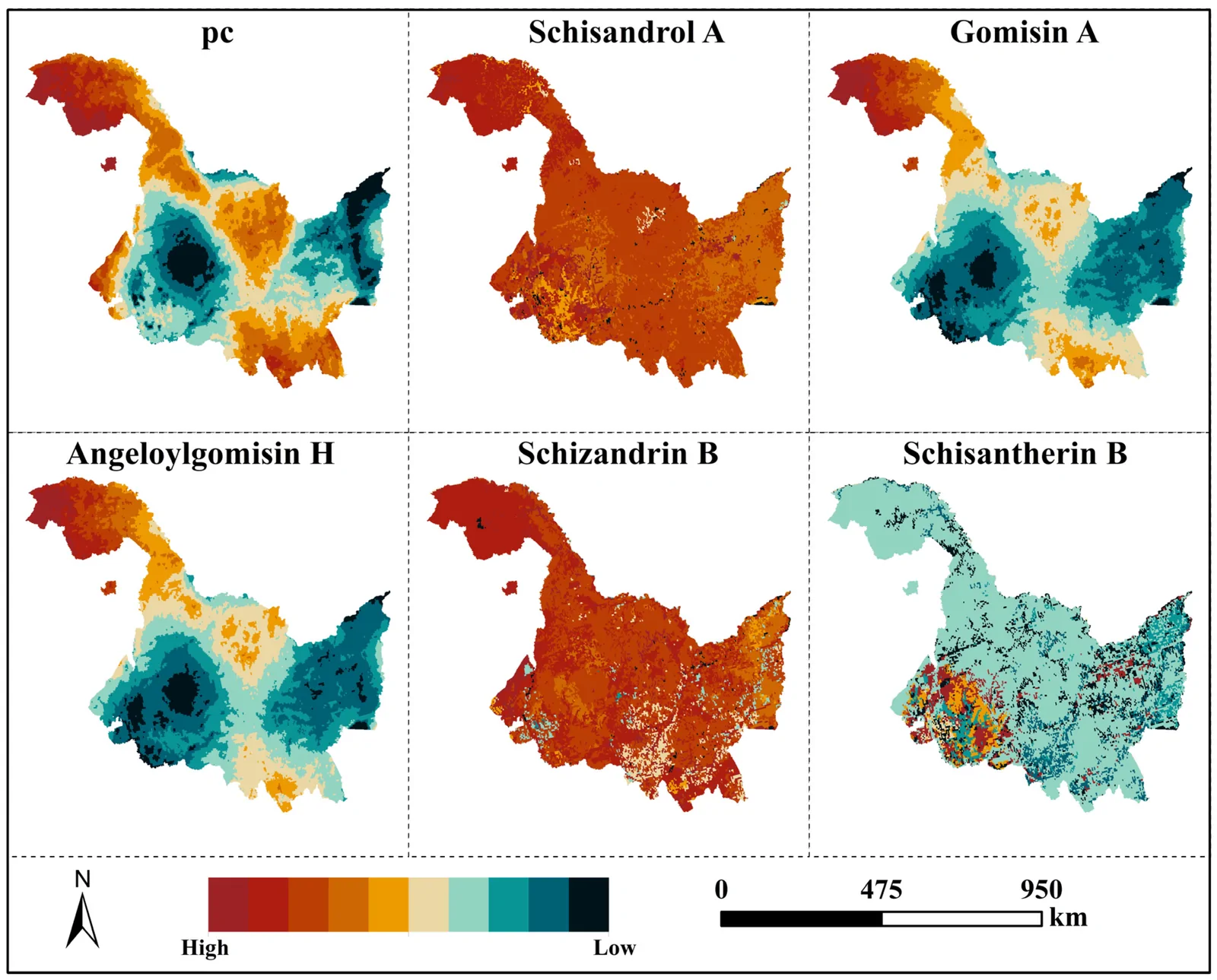
Distribution pattern of various chemical components of *S. chinensis* in Heilongjiang Province under current climate scenarios.

Within Heilongjiang Province, China, the MuMIn package was employed to construct the optimal mathematical correlation model between the fingerprint analysis results and environmental factors. To elucidate the correlation between the spatial distribution of medicinal component contents and environmental factors, the correlation model selects the optimal regression model based on the F-test *p*-value and adjusted R^2^; its formula, adjusted R^2^, and *p*-value are given in Table 2. The derived formula was employed to construct the spatial distribution patterns of the comprehensive components and individual components of *S. chinensis* in Heilongjiang Province. Based on the obtained spatial distribution patterns, the current high-quality distribution of the comprehensive components of *S. chinensis* is concentrated in the Greater Khingan Range and Lesser Khingan Range, and exhibits a patchy distribution in the Zhangguangcai Range and Laoye Range; the current high-quality distribution areas of Schisandrol A in Heilongjiang Province are widely distributed across various regions of the province; the high-quality distribution areas of Gomisin A are mainly concentrated in the Greater Khingan Range, with sporadic distribution in partial areas of the Lesser Khingan Range, Zhangguangcai Range and Laoye Range; the high-quality distribution areas of Angeloylgomisin H are mainly concentrated in the Greater Khingan Range, with sporadic distribution in partial areas of the Lesser Khingan Range, Zhangguangcai Range and Laoye Range; the high-quality distribution areas of Schizandrin B are widely distributed across various regions of Heilongjiang Province; the high-quality distribution areas of Schisantherin B are concentrated in southwestern Heilongjiang, presenting a patchy distribution (Figure 7).

**Figure 8:**
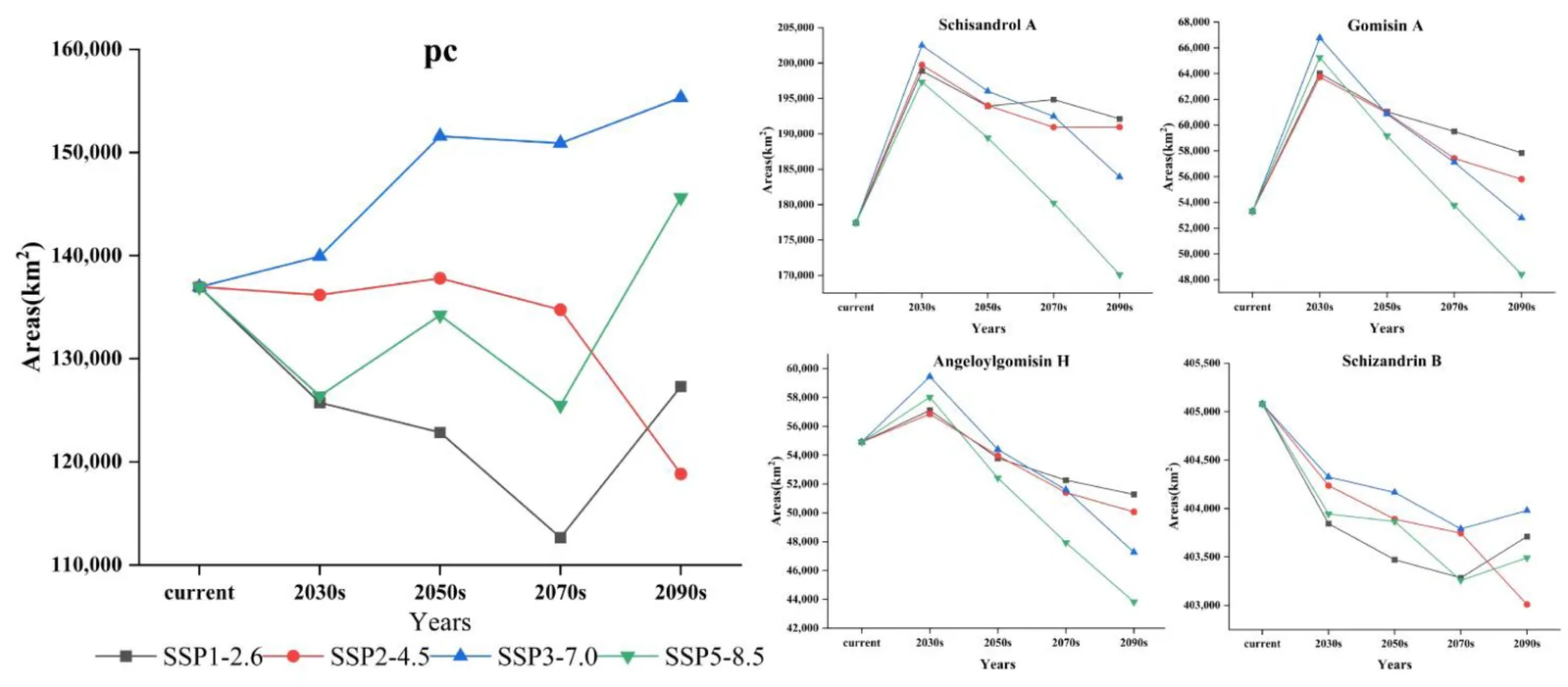
Changes in the areas of high-quality spatial distribution of various components of *S. chinensis* in Heilongjiang Province.

Under future climate change scenarios, the changes in the areas of high-quality regions for the comprehensive components and individual components of *S. chinensis* in Heilongjiang Province are shown in Figure 8. It can be observed that the future change trends in the areas of high-quality regions for the comprehensive components and individual components of *S. chinensis* in Heilongjiang Province differ from one another. Except for the climate scenario SSP3-7.0, the area of high-quality distribution regions of the comprehensive quality of *S. chinensis* shows an overall shrinking trend compared with the current climate scenario across all other scenarios; the area of high-quality distribution regions of Schisandrol A and Gomisin A shows an overall expanding trend compared with the current climate scenario under future climate scenarios; except for the 2030s period, the area of high-quality distribution regions of Angeloylgomisin H shows an overall shrinking trend compared with the current climate scenario under future climate scenarios; the area of high-quality distribution regions of Schizandrin B shows an overall shrinking trend compared with the current climate scenario under future climate scenarios. In addition, the content of Schisantherin B is only significantly correlated with the soil factor t_ph_H_2_O, and this soil factor is not projected to undergo substantial changes in the future [65]. Therefore, it can be concluded that the high-quality distribution area of Schisantherin B in *S. chinensis* in Heilongjiang Province will remain stable.

### 3.6. Planning for S. chinensis Protected Areas and Targeted Wild Tending Areas in Heilongjiang Province Under Global Change

#### 3.6.1. Planning for *S. chinensis* Protected Areas in Heilongjiang Province Under Global Change

**Figure 9:**
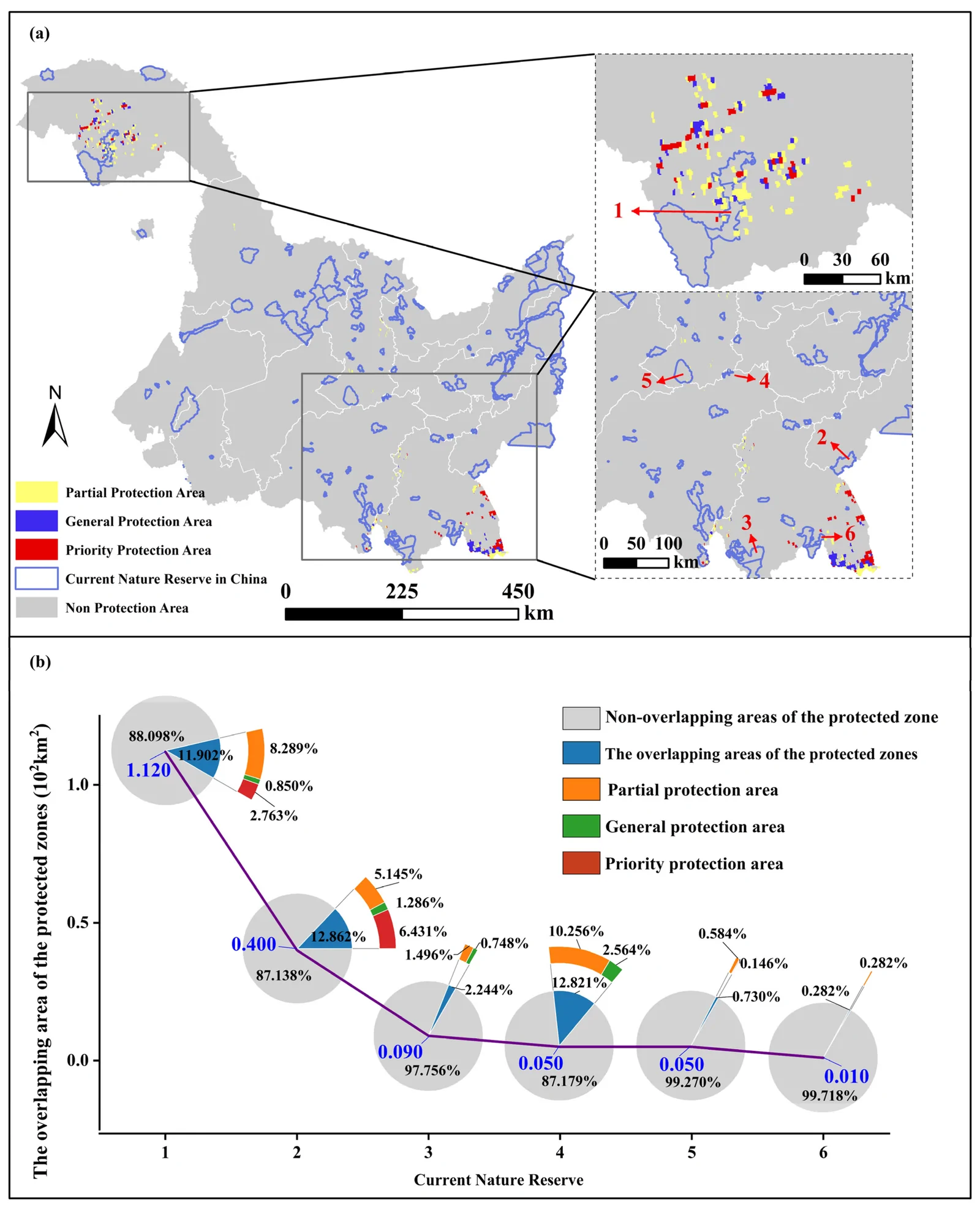
(**a**) The planning of three grades of protection areas for *S. chinensis* in Heilongjiang Province and their overlap with the current protected areas (the red numbers in (**a**) represent respectively: 1: Huzhong National Forest Park; 2: Fenghuangshan National Forest Park; 3: Heilongjiang Xiaobeihu Nature Reserve; 4: Danqinghe National Forest Park; and 5: Xiaoxing’anling National Geopark in Yichun City, Heilongjiang Province; 6: Muling *Taxus cuspidata* National Nature Reserve). (**b**) The overlapping area between the planned *S. chinensis* conservation reserves in Heilongjiang Province and existing conservation reserves, as well as the proportion of conservation reserves at all levels (the blue text in (**b**) indicates the overlapping area between the planned protected areas and the current protected areas).

Against the background of future global climate change, the total area of the protected areas planned in Heilongjiang Province is 4.392 × 10^3^ km^2^, accounting for 20.00% of the stable naturally suitable habitat area, and is mainly distributed in the Greater Khingan Range and Laoyeling Range, with a small portion distributed in the Lesser Khingan Range. Among them, the area of priority protection zones is 1.098 × 10^3^ km^2^, the area of general protection zones is 1.098 × 10^3^ km^2^, and the area of partial protection zones is 2.196 × 10^3^ km^2^. Among the existing protected areas in Heilongjiang Province, there are six protected areas that overlap in area with the overall planned *S. chinensis* protection zones, which are Huzhong National Forest Park, Fenghuangshan National Forest Park, Heilongjiang Xiaobeihu Nature Reserve, Danqinghe National Forest Park, Xiaoxing’anling National Geopark in Yichun City, Heilongjiang Province, and Muling *Taxus cuspidata* National Nature Reserve. Among these overlapping protected areas, the overlapping area with the planned priority protected regions is 0.470 × 10^2^ km^2^, accounting for only 4.281% of the total area of all priority protected areas. Specifically, the planned *S. chinensis* protected area within Huzhong National Forest Park is the largest, at approximately 1.120 × 10^2^ km^2^, which constitutes 11.902% of the total area of Huzhong National Forest Park. Within this protected area, the priority protected zone covers 0.260 × 10^2^ km^2^, the general protected zone covers 0.800 × 10^2^ km^2^, and the partial protected zone covers 0.780 × 10^2^ km^2^ (Figure 9).

#### 3.6.2. Planning of Targeted Wild Tending Areas for *S. chinensis* in Heilongjiang Province Under Global Change

**Figure 10:**
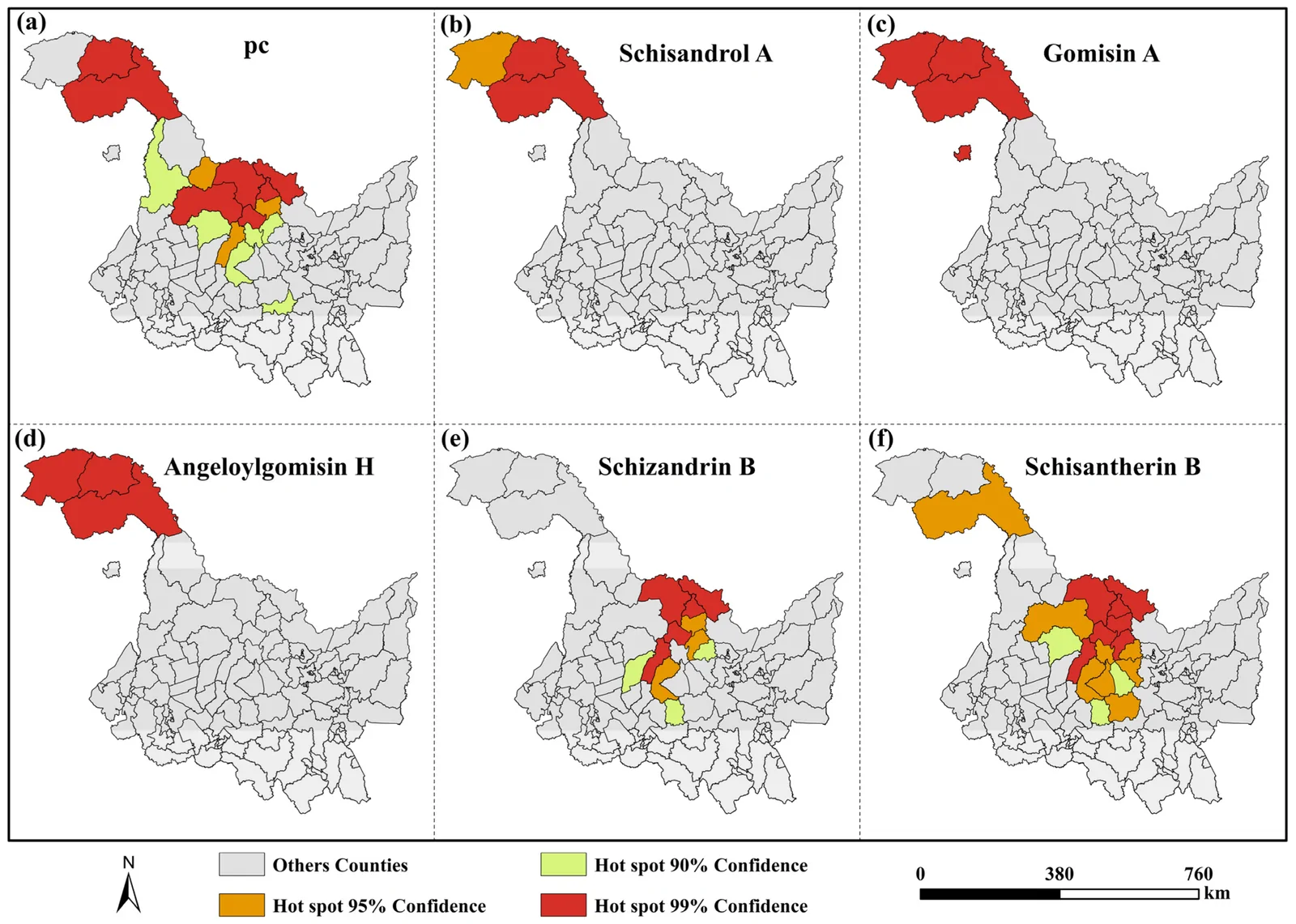
(**a**) Hotspot counties for wild tending of *S. chinensis* with comprehensive active components in Heilongjiang Province under current climate scenarios. (**b**) Hotspot counties for wild tending of *S. chinensis* with high Schisandrol A content in Heilongjiang Province under current climate scenarios. (**c**) Hotspot counties for wild tending of *S. chinensis* with high Gomisin A content in Heilongjiang Province under current climate scenarios. (**d**) Hotspot counties for wild tending of *S. chinensis* with high Angeloylgomisin H content in Heilongjiang Province under current climate scenarios. (**e**) Hotspot counties for wild tending of *S. chinensis* with high Schizandrin B content in Heilongjiang Province under current climate scenarios. (**f**) Hotspot counties for wild tending of *S. chinensis* with high Schisantherin B content in Heilongjiang Province under current climate scenarios.

Under current climatic conditions, the Greater Khingan Range and Lesser Khingan Range are the most suitable regions for the development of wild tending of *S. chinensis* in Heilongjiang Province. Specifically, when targeting high-quality comprehensive components, Huma County and Tahe County in the Greater Khingan Range, multiple counties and districts in the Lesser Khingan Range, and Fangzheng County are hotspot regions for wild tending of *S. chinensis*; when targeting high content of high-quality Schisandrol A, Huma County, Tahe County and Mohe City in the Greater Khingan Range are hotspot regions for wild tending of *S. chinensis*; when targeting high content of high-quality Gomisin A, all four counties and districts (Huma County, Tahe County, Mohe City and Jiagedaqi District) in the Greater Khingan Range are hotspot regions for wild tending of *S. chinensis*; when targeting high content of high-quality Angeloylgomisin H, Huma County, Tahe County and Mohe City in the Greater Khingan Range are hotspot regions for wild tending of *S. chinensis*; when targeting high content of high-quality Schizandrin B, most counties and districts in the Lesser Khingan Range are hotspot regions for wild tending of *S. chinensis*; when targeting high content of high-quality Schisantherin B, most counties and districts in the Lesser Khingan Range are hotspot regions for wild tending of *S. chinensis* (Figure 10).

Under future global change scenarios, the hotspot counties suitable for wild tending of *S. chinensis* with high contents of the comprehensive components Schizandrin B and Schisantherin B show a trend of shifting toward the northwest, accompanied by a decrease in both quantity and hotspot intensity. With the passage of time and increasing carbon emissions, by the 2090s under the SSP5-8.5 scenario, it is projected that only Tahe County and Huma County in the Greater Khingan Range region of Heilongjiang Province will remain as hotspot areas. The hotspot counties suitable for wild tending of *S. chinensis* with high contents of Schisandrol A, Gomisin A and Angeloylgomisin H remain stable and are concentrated in the Greater Khingan Range region. However, with the passage of time and increasing carbon emissions, both the number of hotspot counties and hotspot intensity will also decrease by the 2090s under the SSP5-8.5 scenario (Figures S6–S11).

## 4. Discussion

### 4.1. Improvement and Application of Species Distribution Models in the Prediction of Suitable Areas for S. chinensis

Species distribution models (also known as ecological niche models or habitat suitability models) have become a common tool for addressing fundamental and applied issues related to biodiversity. Nevertheless, limitations of the core principles and standardized operational protocols of species distribution models are commonly observed in empirical research. These limitations are not only reflected in confusion about niche concepts (e.g., fundamental vs. realized niche), but also in the neglect of data quality issues, including species misidentification and collector bias. These factors significantly undermine the ecological validity of model outputs [66,67]. Furthermore, existing studies rely solely on occurrence records provided by open online databases such as GBIF, and fail to incorporate standardized field-collected survey data, which often results in biased prediction outputs [68]. Meanwhile, most regional-scale species distribution modeling still adopts a single algorithm, and does not effectively utilize ensemble modeling approaches such as Biomod2, which have been demonstrated to deliver higher robustness and prediction accuracy in comparative studies [69,70]. Most existing studies employ network-based data sources to simulate species distribution models at regional scales, typically using a single model for analysis [26,27,28,29,30]. Based on this, this study optimizes the prediction of the potential suitable areas for *S. chinensis* (Table 3). Firstly, relying on field investigations, basic databases and the plantR program package, this study constructed a relatively complete dataset of *S. chinensis* species distribution. Through data cleaning and quality control processes, erroneous and abnormal distribution points were eliminated, effectively improving the spatial coverage and prediction stability of the model. Secondly, based on the cleaned and screened valid occurrence points, this study delimits the distribution range of *S. chinensis* to the continental land area of Asia. According to the complete niche characteristics of the species at the Asian scale, this study constructs the potential suitable distribution area covering its entire climate domain, and then clips the prediction results to the study area (Heilongjiang Province). This strategy can effectively prevent the model from conducting unreasonable extrapolation beyond the range of training data when simulating future climate scenarios [71]. Finally, in terms of model algorithms, this study adopts the optimized ensemble model Biomod2, which effectively reduces the instability and systematic bias of a single model through multi-algorithm ensemble. Meanwhile, the Kuenm package is used to optimize the parameter configuration of the MaxEnt model, which avoids model overfitting and enables more accurate prediction of the dynamic changes in the suitable distribution areas of *S. chinensis* under different future climate scenarios [45,72].

### 4.2. Regulatory Effects of Key Environmental Factors on S. chinensis Growth

Based on the suitability area prediction results, the current suitable areas for *S. chinensis* are concentrated in the temperate regions of Northeast Asia, covering northeastern China, the Russian Far East, the Korean Peninsula, and parts of Japan. This region is dominated by a temperate monsoon climate, with climatic variations primarily influenced by meteorological factors such as the East Asian monsoon and tropical cyclones. It also exhibits transitional characteristics of a temperate continental climate. These characteristics include distinct seasons, concurrent rainfall and warmth, cold winters and warm summers, and uneven spatiotemporal distribution of precipitation. Such features align closely with *S. chinensis*’s ecological adaptability to light, moisture, and cold tolerance [34]. Wild *S. chinensis* naturally occurs in habitats such as forest edges and shrublands in mountain valleys, thriving at elevations between 1200 and 1700 m. Moreover, the complex and diverse topography of Northeast Asia, combined with high vegetation cover and rich plant communities, provides a wide range of altitudes, from sea level to 4880 m. This offers extensive and varied ecological niches for the survival and reproduction of *S. chinensis* [73,74,75].

Based on the simulation outputs from the Biomod2 model, the key environmental factors driving the growth of *S. chinensis*, ranked by the magnitude of their influence, are winter solar radiation (srad_winter), precipitation of the warmest quarter (bio18), summer solar radiation (srad_summer) and precipitation of the wettest month (bio13). This indicates that light availability and precipitation are the core environmental factors regulating the growth of *S. chinensis*. In this study, the influences of temperature and soil factors on the geographical distribution of *S. chinensis* were incorporated into model calculation. The results show that their contribution rates are significantly lower than those of precipitation climatic factors and solar radiation factors. It is inferred that although temperature is a critical environmental factor affecting plant distribution, it may have a high collinearity with solar radiation, and its contribution rate is lower than that of solar radiation; therefore, the model does not identify temperature as the dominant factor affecting the distribution of *S. chinensis* [76]. Meanwhile, although soil conditions are the necessary foundation for the growth of *S. chinensis*, they are not the dominant factor restricting its growth and distribution. This conclusion is consistent with the research findings reported by L [77].

For individual plants, the accumulation of appropriate light radiation is critical for initiating their life cycle, namely seed germination, seedling growth and flowering [78]. *S. chinensis* is a climbing vine plant. Climbing plants feature a high light saturation point, which is reached when the light level is close to the higher light intensity of the habitat. This trait ensures that a relatively high photosynthetic rate can be maintained throughout the entire lifespan of the leaves. In addition to a high light saturation point, climbing plants also possess a low light compensation point, and many species growing under high light conditions exhibit a low light compensation point [79]. Since plants cannot accumulate dry matter at the light compensation point, the level of this point is significant for determining whether plants can thrive under low light intensity [80]. *S. chinensis*, as a sun-loving yet shade-tolerant plant, relies heavily on light for its growth. Being a monoecious species, light plays a crucial regulatory role in the differentiation of male and female flowers in *S. chinensis*. Li et al. found that *S. chinensis* growing in open areas and forest edges flower earlier than those in the forest interior, with fruit ripening occurring 4–7 days earlier as well; furthermore, the proportion of female flowers in wild northern *S. chinensis* shows an increasing trend from forest interior to forest edge to open areas [81]. The differentiation of male and female flowers in *S. chinensis* begins in late July, reaching its peak during mid- to late August, indicating that suitable light conditions in summer significantly influence the plant’s fruiting capacity [82]. In mid- to high-latitude regions of the Northern Hemisphere (40–90° N), solar radiation accumulates over a period of 1–3 months without lag, so winter solar radiation can promote shoot regreening and bud break in *S. chinensis* [78,82]. Under global warming, the combination of rising winter temperatures and abnormal short photoperiods can lead to premature deacclimation of trees and reduced frost resistance. This, in turn, limits plant distribution to some extent, because subsequent cold snaps or late frosts can cause frost damage [83].

Precipitation regulates plant growth by influencing physiological processes, soil moisture, and nutrient availability [84]. *S. chinensis* is a climbing plant with well-developed root systems that can efficiently utilize soil water [85]. When precipitation decreases, plants allocate more biomass to roots to enhance water uptake [86]. Under such conditions, *S. chinensis* may adopt a differential response strategy, characterized by increased root biomass while aboveground growth is correspondingly suppressed [87]. In contrast, high precipitation can enhance soil nitrogen mineralization and plant nitrogen use efficiency, thereby increasing total plant biomass [88]. However, excessive rainfall leads to soil saturation, which reduces the partial pressure of oxygen around plant roots. This typically decreases root permeability, limiting water uptake. Thus, despite ample moisture in the soil, plants may still experience water deficit [89]. As a monoecious plant, the differentiation of male and female flowers in *S. chinensis* is also influenced by precipitation. Zhao et al. hypothesized that, according to resource allocation theory, drought in the previous year could lead to adjustments in sex expression of *S. chinensis*, specifically a decrease in the proportion of female flowers [90]. This study shows that the suitable growing regions for *S. chinensis* are concentrated in temperate northeastern Asia, where the wettest month and warmest season coincide with summer. Concentrated summer rainfall can cause abnormal physiological metabolism in *S. chinensis*, often resulting in flower and fruit drop [91]. Moreover, Seo et al.’s research indicated that excessive precipitation increases soil moisture content, leading to reduced photosynthetic and transpiration rates in *S. chinensis* [92].

### 4.3. Spatiotemporal Migration Patterns and Survival Risk Assessment of S. chinensis Habitats in Heilongjiang Province Under Global Change

The coupled mechanisms of climate change, species’ ecological adaptability, and land use changes jointly determine the future spatiotemporal evolution patterns and dynamic distribution characteristics of *S. chinensis* habitats in Heilongjiang Province [18]. Under future climate change scenarios, *S. chinensis* responds to climatic stress by shifting its habitat spatially and temporally. This response is driven by its ecological niche characteristics. These include temperature and humidity adaptability, soil preferences, and phenological rhythms. Meanwhile, land use exerts an additive disturbance effect on this habitat migration process through landscape pattern restructuring and changes in habitat connectivity. The results show that under the low-emission SSP1-2.6 scenario, the habitat area of *S. chinensis* remains dynamically stable overall. This is closely related to the fact that regional temperature rise in this scenario is kept below 2.0 °C and precipitation patterns remain relatively stable, resulting in no significant shift in the species’ habitat range. The species can gradually adapt to mild climate change through its own ecological plasticity [93,94]. Under the SSP2-4.5, SSP3-7.0 and SSP5-8.5 carbon emission scenarios, the area of *S. chinensis* habitats in Heilongjiang Province shows a gradual decreasing trend over time, and the suitable habitats are gradually shifting to the Greater Khingan Range region in the northwest direction. As a typical temperate deciduous woody vine, the growth and development of *S. chinensis* has a strict adaptive requirement for humidity thresholds [82]. Under the medium-high emission scenario, alterations in precipitation patterns have intensified the aridity degree in Heilongjiang Province. *S. chinensis* features shallow root systems and prefers moist, fertile soils with a pH value ranging from 5.5 to 7.0. Only sufficient water supply during the growing season can promote its growth, flowering and fruit set, while soil water deficit will further exacerbate the unsuitability of habitats [34,95]. In contrast, the Greater Khingan Range region, influenced by monsoon circulation and topographic lifting, has more uniform precipitation distribution. Furthermore, the humus layer formed by the decomposition of coniferous forest litter can effectively maintain soil moisture, which meets the soil ecological requirements of *S. chinensis* [96,97].

Land use changes may damage species habitats and the connectivity between them, preventing species from dispersing from their current habitats to future suitable habitats, which in turn increases the risk of species extinction [98]. It is projected that by 2050, 10% to 20% of natural grasslands and forests will be replaced by agricultural and urban infrastructure [99]. The reduction in forest area will result in a lack of climbing attachment substrates for wild *S. chinensis*, exerting adverse impacts on the growth of this species. Putz et al. noted that the population density of climbing plants will decline over time when they lack adequate attachment substrates [100]. The flowering and fruiting rate of *S. chinensis* is closely correlated with light conditions, thus this species generally exhibits a higher fruiting rate in forest edge habitats [81]. However, land use change inevitably leads to the reduction in forest area in the natural habitats of *S. chinensis*, and the forest edge zones suitable for its flowering and fruiting are the first to be damaged, which severely threatens the survival of *S. chinensis* populations [101]. In conclusion, under the combined pressure of future climate change and land use change, the area of suitable habitats for *S. chinensis* in Heilongjiang Province will inevitably shrink, and its spatial distribution centroid is projected to migrate to the Greater Khingan Range forest area in the northwest direction. This result indicates that the current conservation system located in southeastern Heilongjiang will face the risk of failure, and future conservation planning should be expanded to the Greater Khingan Range region, while strengthening the management and control of land use along the migration corridors.

### 4.4. Spatial Response Characteristics and Environmental Driving Mechanisms of the Chemical Constituents of S. chinensis in Heilongjiang Province

In this study, the main secondary metabolites of *S. chinensis* were determined, including Schisandrol A, Gomisin A, Angeloylgomisin H, Schizandrin B, Schisantherin B and Schizandrin A. All of these metabolites are lignans, which belong to phenylpropanoids, and their biosynthesis is initiated via the phenylpropanoid metabolic pathway [102]. With the exception of Schizandrin A, the contents of all other components exhibited significant responses to environmental factors, and the spatial response patterns of different components showed obvious differentiation. This difference may be closely associated with their respective metabolic branches and regulatory mechanisms.

The results of all-subsets regression show that the lignin content of *S. chinensis* in this study is correlated with temperature, precipitation, soil properties, solar radiation and other environmental factors. Among these factors, lignin content exhibits a significant positive correlation with temperature factors. Existing studies have demonstrated that high temperature can activate the glycolysis pathway of *S. chinensis*, thereby increasing the content of phenylalanine. As a precursor substrate for lignin biosynthesis in *S. chinensis*, the accumulation of phenylalanine will further promote lignin synthesis in *S. chinensis* [103]. Excessive precipitation inhibits lignin accumulation in *S. chinensis*. Under normal circumstances, drought stress suppresses the physiological processes of numerous plant species, thereby hindering their growth and development [104]. Nevertheless, plants have evolved drought adaptation mechanisms mediated by the phenylpropanoid biosynthetic pathway [105]. Earlier studies have demonstrated that up-regulated expression of flavanone 3-hydroxylase (F3H), phenylalanine ammonia-lyase (PAL), 4-coumarate-CoA ligase (4CL) and flavonol synthase (FLS) can enhance plant drought tolerance; furthermore, elevated activity of PAL and 4CL also promotes lignin biosynthesis [102,106]. This study reveals that the content of Gomisin A is negatively correlated with bio14 (precipitation of the driest month), indicating that moderate drought stress facilitates the accumulation of Gomisin A. Studies conducted by Ri et al. demonstrate that moderate UV-B radiation can induce *S. chinensis* to activate stress resistance mechanisms and promote the accumulation of stress-resistant secondary metabolites such as lignin; however, excessive UV-B radiation inhibits the accumulation of certain key stress-resistant secondary metabolites, such as the phenolic compound 4-hydroxycinnamic acid. 4-hydroxycinnamic acid, as a precursor for lignin biosynthesis in *S. chinensis*, leads to a reduction in lignin synthesis when its content decreases [102,107,108]. This result is consistent with the conclusion obtained in this study that excessive summer solar radiation inhibits the accumulation of partial lignin in *S. chinensis*. Different from the monotonic effects of temperature, precipitation and solar radiation on the lignin accumulation in *S. chinensis*, the regulatory effect of soil factors is more complex. Since it involves multiple aspects including pH, cation exchange capacity and soil texture, its impact on lignin accumulation exhibits multi-directionality and heterogeneity. The stronger the cation exchange capacity (CEC) of clay soil, the more effectively it can retain and supply cation nutrients required by plants (e.g., K^+^). An increase in K^+^ content can raise the content of lignin monomers, thereby providing a material basis for the synthesis of *S. chinensis* lignin [109]. In contrast, across a broader range of soil types, CEC exhibits a negative correlation with the lignin content of *S. chinensis*. We hypothesize that this phenomenon is primarily associated with soil texture. Loam and sand show a negative correlation with CEC. They generally have favorable drainage without waterlogging, but are prone to intermittent drought, and this drought promotes secondary metabolism. On the other hand, an excessive increase in CEC, especially when induced by very high clay content, tends to worsen soil aeration and hydraulic conductivity [110,111,112,113,114]. This extreme physical edaphic environment restricts root activity, and is thus unfavorable for the accumulation of plant secondary metabolites [115]. Soil pH influences the lignin content in *S. chinensis*. It is hypothesized that soil pH indirectly mediates the regulation of lignin biosynthesis in *S. chinensis* via rhizosphere microorganisms, such as *Arthrobacter* and *Paenibacillus* [116]. This study demonstrates that the volume percentage of gravel in topsoil (t_gravel) is negatively correlated with lignin accumulation in *S. chinensis*. An increase in soil gravel content reduces the available water and nutrient contents in topsoil [117]. To adapt to such resource limitations, plants typically promote root growth into deeper soil layers to acquire water and nutrients [118]. During this process, water stress can induce increased lignin synthesis and deposition in roots, which is manifested as enhanced root lignification [119]. Based on the theoretical hypothesis of carbon allocation trade-off [120], this process may lead to a reduction in lignin allocated to other organs, such as fruits. However, there is currently no direct experimental evidence to support this inference, and further research is required to verify it.

### 4.5. Conservation and Utilization of S. chinensis in Heilongjiang Province Under Global Change: Challenges and Countermeasures

The ability of a population to adapt to climate change depends, to a certain extent, on the average lifespan of individuals and their age at reproductive maturity [121]. As a tree species with long generation time and lifespan, *S. chinensis* has a weaker capacity than annual plants to establish new genotypes and adapt to rapid climate change. Furthermore, field investigations reveal that although *S. chinensis* is widely distributed across forests, it almost fails to flower and fruit in environments with high canopy closure, and relies primarily on subterranean rhizomes for asexual reproduction. This trait weakens the ability of *S. chinensis* to generate new genotypes, and in turn reduces its adaptability to rapid climate change. Meanwhile, due to anthropogenic activities including agricultural expansion, timber harvesting, infrastructure construction and urbanization, the forest habitats that *S. chinensis* depends on for survival will be subjected to tremendous pressure [122]. Furthermore, as a significant genuine medicinal material in Heilongjiang Province, *S. chinensis* has extremely high medicinal value. However, driven by economic interests, some pickers adopt destructive harvesting methods such as cutting vines and felling trees to obtain temporary convenience during fruit collection, engaging in predatory exploitation [123]. This behavior has caused severe damage to wild *S. chinensis* resources, resulting in a sharp decline that is of great concern and urgently requires high-level attention. Accordingly, this study comprehensively incorporates two core driving factors, namely climate change and land use change, and systematically delineates priority conservation areas for *S. chinensis* in Heilongjiang Province, with the aim of providing urgently needed theoretical basis for government departments to formulate resource conservation strategies for *S. chinensis*. All protected areas planned in this study are forest habitats that can maintain climate suitability by the end of this century, interference zones such as residential areas and cultivated land were excluded. On the basis of Zonation prioritization ranking, we set the conservation target as the top 20% of high-priority grid cells. Existing literature shows that protecting 5–20% of high-quality habitats can cover the core populations of more than half of the target species. Therefore, this threshold has sufficient basis for biodiversity conservation, while avoiding land conflicts caused by blind boundary expansion, and is feasible in practical operation [124]. In addition, we applied Zonation to protected area planning in this study. The algorithm itself embeds spatial proximity weights during the iterative removal process, so that the preferentially retained grid cells naturally tend to be aggregated in distribution rather than forming scattered patches. This ensures that the selected sites can support the migration and gene flow of *S. chinensis* populations under the background of climate change [125].

The results show that although the current suitable habitats of *S. chinensis* already cover some national and provincial nature reserves, under future climate change scenarios, existing nature reserves only cover 4.28% of the total area of priority conservation areas. A large number of high-conservation-value areas have not yet been incorporated into the conservation system, resulting in a significant conservation gap. Among existing protected areas, Huzhong National Forest Park has the largest area of priority conservation zones. Its spatial units provide highly suitable climatic conditions for *S. chinensis* growth, and the region’s climate is expected to remain relatively stable over the next century, minimizing potential impacts of climate change on *S. chinensis* populations. Additionally, Fenghuangshan National Forest Park, Xiaobeihu Nature Reserve in Heilongjiang, Danqinghe National Forest Park, Xiao Xing’anling National Geopark in Yichun City, Heilongjiang Province, and Muling *Taxus cuspidata* National Nature Reserve can also contribute to the conservation of *S. chinensis* in Heilongjiang Province. Based on the aforementioned characteristics, Huzhong National Forest Park is expected to become the core region for the conservation of *S. chinensis* within this century, providing critical support for the population maintenance and long-term persistence of this species. Meanwhile, we recommend establishing monitoring sites in areas with dense *S. chinensis* populations within the six protected areas that exhibit a large overlapping area with the planned protected areas, and conducting regular assessments of the population distribution dynamics. This will ensure that the population size in climatically stable regions remains unaffected by other external disturbances, and facilitate the timely development of adaptive management measures. In addition, we recommend that district- and county-level governments guide the establishment of provincial-level germplasm resource protection bases for S. chinensis. This recommendation specifically targets protection gap areas, such as the Greater Khingan Range region and the Laoye Range region, where such gaps are large. Such bases should implement in situ conservation for native populations to stabilize community structure and maintain genetic diversity. In addition, they should systematically collect excellent local germplasm resources and conduct experiments on seedling propagation, population scale expansion, and wild tending. The ultimate goal is to gradually expand population size and restore degraded habitats.

In addition to protecting wild *S. chinensis* resources, artificial cultivation has become an important approach to supply *S. chinensis* medicinal materials to meet the growing demand of the medical market. Against the background of the policies of “non-grain cultivation” and “non-agricultural utilization” of cultivated land, the understory wild tending mode, which does not occupy cultivated land resources, has become an effective way to alleviate the supply pressure of *S. chinensis* medicinal materials. In this study, when planning the wild tending areas for *S. chinensis* in Heilongjiang Province, global change and the demand for different active components of *S. chinensis* were comprehensively considered, and targeted wild tending areas that balance adaptability to global change and meet the demand for multiple components were constructed. The planned areas can not only ensure that the climatic conditions remain continuously suitable for the growth of *S. chinensis* throughout this century, but also directionally provide a variety of high-quality *S. chinensis* components in response to different market demands. Meanwhile, this study provides a reference for the commercial cultivation of *S. chinensis*, thereby alleviating the shortage of wild *S. chinensis* resources to the maximum extent. According to the planning, Tahe County and Huma County in the Greater Khingan Range region are delimited as the most suitable areas for wild tending of *S. chinensis*. This region can guarantee a continuous and stable supply of high-quality *S. chinensis* medicinal materials throughout this century. Based on the planning results, we put forward the following recommendations: (1) Wild tending of *S. chinensis* is concentrated in hotspots such as Tahe County and Huma County. In these areas, local governments should be guided to formulate targeted policies and provide supportive measures including technical guidance for tending, so as to encourage enterprises and individuals to conduct commercial simulated wild cultivation. This measure can not only reduce the consumption of wild resources through artificial field cultivation and alleviate the pressure on the supply of medicinal materials, but also promote the population proliferation and habitat restoration of *S. chinensis* through human intervention. (2) Priority should be given to the systematic collection and ex situ conservation of high-quality *S. chinensis* germplasm resources in regions such as the Greater Khingan Range. Excellent germplasm resources from different geographical origins with significant differences in active ingredient content shall be extensively collected. Meanwhile, efforts shall be made to promote the breeding and large-scale propagation of improved varieties, so as to provide high-quality germplasm foundation for the recovery of wild populations and the development of industrial cultivation.

## 5. Conclusions

Against the background of global change, this study constructs suitable habitats for *S. chinensis* in Heilongjiang Province under climate stabilization scenarios based on climate change and land use change. Combined with the scope of current protected areas, this research systematically identifies existing conservation gaps and scientifically plans priority protected areas for *S. chinensis* in Heilongjiang Province. On this basis, combined with high-performance liquid chromatography and principal component analysis, this study accurately identifies the high-quality spatial distribution of various components of *S. chinensis* through all-subsets regression analysis, and ultimately identifies the core hotspot counties for wild tending of *S. chinensis* in Heilongjiang Province. Although some scholars currently question the prediction results of species distribution models, this study holds that as long as we build on the basis of accurate and comprehensive species distribution data, systematically integrate multi-dimensional influencing factors such as climate change and land use pattern change, and further optimize the screening scheme for model parameters and environmental variables, the prediction results of the model still have important academic reference value and practical guiding significance. In conclusion, this study lays a solid scientific theoretical foundation for the conservation and directed cultivation planning of wild *S. chinensis* populations in Heilongjiang Province, and can provide accurate and scientific technical guidance for wild tending practices conducted by local forestry authorities and relevant industrial entities. Accordingly, it can effectively improve the quality and efficiency of *S. chinensis* resource cultivation and facilitate the high-quality development of the traditional Chinese medicine characteristic industry. In addition, the optimized approach proposed in this study for integrating multi-dimensional impact factors into species distribution models also provides a practical methodological reference for subsequent habitat suitability assessment and sustainable resource utilization planning of similar endangered medicinal plants.

## 6. Limitations and Future Research Directions

This study has several limitations. (1) When constructing the assessment of future habitat suitability, this study only adopts forest distribution change as a surrogate indicator of land use change, and does not incorporate such ecological factors as forest quality, habitat fragmentation, canopy structure, disturbance intensity and the differences between natural forests and planted forests. This may introduce certain biases into the estimation of the future habitat availability of *S. chinensis*. (2) It is noteworthy that since the present study aims to elucidate the correlation between the spatial distribution of medicinal component contents and environmental factors, the regression model of chemical components constructed for spatial prediction has not been validated. Accordingly, the research results only provide reference value for the selection of the optimal regression model in this study, and there are certain limitations regarding the validity of the prediction. (3) This study only addresses the correlation between the content of medicinal components in *S. chinensis* and external abiotic environment; however, the medicinal quality of *S. chinensis* may also be affected by factors such as genetic variation and soil microorganisms. In response to the above limitations, future research will be conducted from the following perspectives: (1) At the level of habitat suitability assessment, although the current practice of using forest distribution as a substitute for land use change is feasible, it obviously simplifies the heterogeneity of forest habitats. In the next step, we plan to integrate multi-source remote sensing and ground plot data, incorporate stand canopy density, edge effect index, vertical canopy structure parameters and disturbance history into the model, and distinguish the functional differences between natural secondary forests and pure planted forests in providing habitat resources. (2) In response to the limitations of the present study regarding the validity validation of regression models, future research will address these deficiencies from the following aspects: strategies including cross-validation, external validation with independent samples, and leave-one-out cross-validation will be introduced to systematically evaluate the predictive performance of the selected model on sample points that are not involved in model construction, so as to improve the accuracy and credibility of model prediction. In addition, future research will continue to conduct supplementary field sampling in other regions, further enrich the sample collection from different regions, and enhance the practical application significance of the model in large-scale research. Through the establishment of the above multi-layer validation system, this study expects to ensure the rigor of prediction while providing more solid theoretical support for the correlation analysis between the spatial distribution of medicinal components and environmental factors. (3) Regarding the quality formation mechanism, the current research only focuses on the statistical correlation of abiotic environmental factors, which is still insufficient to reveal the regulatory network of the accumulation of medicinal components. Future research should clarify the genetic differentiation level of populations in different distribution areas, synchronously collect rhizosphere soil samples for metagenomic sequencing, and analyze the coupling relationship between core microbial groups (e.g., mycorrhizal fungi, nitrogen-fixing bacteria) and the lignan biosynthesis pathway. On this basis, we will distinguish the relative contributions of genetic background, soil biota and abiotic environment to quality traits, so as to provide a more causal-based discrimination framework for the zoning of high-quality medicinal materials.

## Figures and Tables

**Table 1:** VIF values of environmental factors.

Factors	VIF	Factors	VIF
bio02	2.581	t_caco3	1.658
bio08	2.538	t_caso4	1.347
bio13	6.192	t_clay	1.805
bio14	4.774	t_esp	1.289
bio15	3.099	t_gravel	1.258
bio18	4.295	t_oc	1.190
bio19	4.843	t_sand	1.948
srad_summer	2.843	t_silt	1.932
srad_winter	3.672		

**Table 2:** Optimal regression models for the contents of 6 chemical components.

Response Variable	Optimal Linear Model	*R* ^2^_adj_	*p*-Value
y_pc_	y_pc_ = 0.3364 × bio03	0.09298	0.02228 *
y_Schisandrol A_	y_Schisandrol A_ = 0.3326 × bio02 + 0.7590 × t_cec_clay − 0.5768 × t_cec_soil + 0.3517 × t_ph_H_2_O	0.15	0.02982 *
y_Gomisin A_	y_Gomisin A_ = 0.2690 × bio02 − 0.2949 × bio14 − 0.3111 × srad_summer	0.2057	0.005298 **
y_Angeloylgomisin H_	y_Angeloylgomisin H_ = 0.4163 × bio02 − 0.3522 × srad_summer	0.1755	0.005934 **
y_Schizandrin B_	y_Schizandrin B_ = 0.3117 × bio03 − 0.2791 × t_gravel	0.1054	0.03435 *
y_Schisantherin B_	y_Schisantherin B_ = 0.3294 × t_ph_H_2_O	0.08823	0.0254 *

*: *p* < 0.05; **: *p* < 0.01.

**Table 3:** Comparison of data sources, models, and climate scenarios used in the simulation study of *S. chinensis* potential suitable zones.

Serial Number	Modeling Area	Climate Scenarios	Distribution Data Source	Model	Source
1	China	Current and future 5 climate scenarios	Mainly derived from field surveys, online species distribution databases, and literature searches	MaxEnt	[26]
2	Republic of Korea	Current and future 5 climate scenarios	Primarily from field surveys	MaxEnt	[27]
3	Northeastern China’s three provinces and northeastern Inner Mongolia	Current climate scenario	Primarily sourced from the Species Distribution Online Database and literature searches	Fuzzy matter-element model	[28]
4	China	Current climate scenario	Primarily sourced from the Species Distribution Online Database	MaxEnt	[29]
5	China	Current and future 10 climate scenarios	Primarily sourced from the Species Distribution Online Database	MaxEnt	[30]
6	Asia (full ecological niche)	Current and future 17 climate scenarios	Primarily sourced from field surveys, online species distribution databases, literature searches, and the plantR package	Biomod2 model	This Study

## Data Availability

The original contributions presented in this study are included in the article/Supplementary Materials. Further inquiries can be directed to the corresponding authors.

## Supplementary Materials

The following supporting information can be downloaded at: https://www.mdpi.com/article/10.3390/biology15171501/s1, Figure S1: Results of principal component analysis (PCA); Figure S2: Model assessment results of the main environmental factors; Figure S3: (a) The area of each suitable habitat for *S. chinensis* in the full ecological zone (Asia); (b) The suitable habitat areas for various grades of *S. chinensis* in Asian countries; (c) The suitable habitat areas for various grades of *S. chinensis* in Chinese provinces; (d) The suitable habitat areas for various grades of *S. chinensis* in cities of Heilongjiang Province; Figure S4: Future changes in key environmental factors; Figure S5: Similarity of *S. Chinensis* from 46 batches in Heilongjiang Province; Figure S6: Distribution of high-quality comprehensive constituents (pc) hotspot counties for wild tending of *S. chinensis* in Heilongjiang Province under future global change; Figure S7: Distribution of high-quality Schisandrol A hotspot counties for wild tending of *S. chinensis* in Heilongjiang Province under future global change; Figure S8: Distribution of high-quality Gomisin A hotspot counties for wild tending of *S. chinensis* in Heilongjiang Province under future global change; Figure S9: Distribution of high-quality Angeloylgomisin H hotspot counties for wild tending of *S. chinensis* in Heilongjiang Province under future global change; Figure S10: Distribution of high-quality Schizandrin B hotspot counties for wild tending of *S. chinensis* in Heilongjiang Province under future global change; Figure S11: Distribution of high-quality Schisantherin B hotspot counties for wild tending of *S. chinensis* in Heilongjiang Province under future global change; Table S1: Environment variables used in MaxEnt model; Table S2: Initial result of MaxEnt model; Table S3: Correlation analysis of environmental factors (Percent contribution > 0%,Correlation coefficient |r| ≤ 0.8); Table S4: TSS values of individual models in Biomod2; Table S5: Ranking of environmental factors based on XGBoost-SHAP analysis; Table S6: Environmental factors in full subset regression; Table S7: Average values of main environmental factors, 95th percentile, minimum value and maximum value; Table S8: Changes in the suitable habitat of *S. chinensis* in Heilongjiang Province under different future shared socioeconomic pathway scenarios; The materials and methods employed for fingerprinting are also presented in the Supplementary materials (Supporting Information of The Chromatographic Fingerprint) [126,127,128].
