# Supplementary material for: Conservation and Targeted Wild Tending of Schisandra chinensis Under Global Change: Informed by SDMs and Fingerprint Analysis of Climate–Land–Composition Responses
Source: Biology (Basel). 2026 Sep 2;15(17):1501. doi: 10.3390/biology15171501 (PMC13564547; doi:10.3390/biology15171501)
Supplement: Supplementary file 1 [file biology-15-01501-s001.zip › biology-4484810-supplementary.pdf]

Supporting Document:

Table S1 Environment variables used in MaxEnt model.

Category	Variable	Description	Unit
	bio01	Annual mean temperature	℃
	bio02	Annual mean diurnal range	℃
	bio03	Isothermality	%
	bio04	Temperature seasonality	-
	bio05	Max temperature of warmest month	℃
	bio06	Min temperature of coldest month	℃
	bio07	Annual temperature range	℃
	bio08	Mean temperature of wettest quarter	℃
	bio09	Mean temperature of driest quarter	℃
**climate**	bio10	Mean temperature of warmest quarter	℃
	bio11	Mean temperature of coldest quarter	℃
	bio12	Annual precipitation	mm
	bio13	Precipitation of wettest month	mm
	bio14	Precipitation of driest month	mm
	bio15	Precipitation seasonality (CV)	mm
	bio16	Precipitation of wettest quarter	mm
	bio17	Precipitation of driest quarter	mm
	bio18	Precipitation of warmest quarter	mm
	bio19	Precipitation of coldest quarter	mm
	t_ph_H_2O	pH Value (H_2O)	-log(H^+)
	t_oc	Soil Organic Matter	g/100 g
	t_esp	Exchangeable sodium in the upper - layer soil	%
	t_clay	Clay content in the upper - layer soil	%wt.
	t_gravel	Gravel content in the upper - layer soil	%vol.
	t_silt	Silt content in the upper - layer soil	%wt.
**soil**	t_sand	Sand content in the upper - layer soil	%wt.
	t_caso4	Sulfate content in the upper - layer soil	%weight
	t_caco3	Carbonate or lime content in the upper - layer soil	%weight
	t_cec_soil	Cation exchange capacity of the upper - layer soil	cmol/kg
	t_cec_clay	Cation exchange capacity of the clay layer in the upper - layer soil	cmol/kg
	t_ece	Electrical conductivity of the upper - layer soil	dS/m
	Srad_spring	Solar radiation from MAR to MAY	$kJ \cdot m^{-2} \cdot day^{-1}$
**Solar**	Srad_summer	Solar radiation from JUN to AUG	$kJ \cdot m^{-2} \cdot day^{-1}$
**radiation**	Srad_autumn	Solar radiation from SEP to NOV	$kJ \cdot m^{-2} \cdot day^{-1}$
	Srad_winter	Solar radiation from DEC to FEB	$kJ \cdot m^{-2} \cdot day^{-1}$

Table S2 Initial result of MaxEnt model.

Variable	Percent contribution	Permutation importance
**bio18**	40.9	0.1
**srad_winter**	13.5	2.3
**bio11**	13.2	0
**bio14**	8.5	3.8
**bio04**	4.9	3.3
**bio06**	4.4	17
**bio13**	3.6	22.9
**bio01**	3.4	8
**bio16**	0.8	0.6
**srad_summer**	0.8	14.4
**bio09**	0.7	1.6
**bio10**	0.7	2.9
**srad_spring**	0.7	3.9
**bio02**	0.6	1
**t_caso4**	0.5	0
**bio12**	0.4	2.5
**bio17**	0.4	2.8
**bio07**	0.4	0.3
**t_sand**	0.2	0.4
**bio08**	0.2	0.4
**t_gravel**	0.2	0.1
**srad_autumn**	0.2	6.6
**t_caco3**	0.1	0.1
**t_oc**	0.1	0.3
**bio03**	0.1	0.9
**bio19**	0.1	1.5
**t_silt**	0.1	0.2
**t_cec_soil**	0.1	0.2
**t_esp**	0.1	0
**t_clay**	0.1	0.1
**bio15**	0.1	0.3
**t_ph_H_2O**	0	0.3
**t_cec_clay**	0	0.1
**t_ece**	0	0.6
**bio05**	0	0.6

Table S3 Correlation analysis of environmental factors (Percent contribution > 0%,Correlation coefficient |r| ≤ 0.8).

FACTORS	t_silt	bio02	bio08	bio13	bio14	bio15	bio18	bio19	srad_summer	srad_winter	t_caco3	t_caso4	t_clay	t_esp	t_gravel	t_oc	t_sand
**t_silt**	0.000	-0.180	-0.150	-0.020	0.009	-0.120	0.019	-0.020	-0.150	-0.180	0.044	0.061	0.350	0.026	0.197	0.240	-0.540
**bio02**	0.000	0.000	0.182	-0.270	-0.320	0.570	-0.340	-0.290	0.590	0.287	0.304	0.119	0.006	0.159	-0.020	-0.120	0.191
**bio08**	0.000	0.000	0.000	0.505	0.184	0.401	0.414	0.199	0.021	0.695	0.125	0.038	0.289	0.074	-0.100	-0.040	0.079
**bio13**	0.000	0.000	0.000	0.000	0.408	0.155	0.783	0.557	-0.350	0.505	-0.190	-0.080	0.281	-0.080	0.041	0.009	-0.060
**bio14**	0.000	0.000	0.000	0.000	0.000	-0.440	0.524	0.790	-0.290	0.165	-0.140	-0.050	0.152	-0.070	0.005	0.116	-0.050
**bio15**	0.000	0.000	0.000	0.000	0.000	0.000	-0.070	-0.300	0.288	0.507	0.176	0.053	0.078	0.111	0.056	-0.170	0.091
**bio18**	0.000	0.000	0.000	0.000	0.000	0.000	0.000	0.417	-0.480	0.274	-0.240	-0.110	0.200	-0.140	0.013	0.064	-0.030
**bio19**	0.000	0.000	0.000	0.000	0.000	0.000	0.000	0.000	-0.150	0.281	-0.100	-0.030	0.183	-0.040	0.043	0.070	-0.060
**srad_summer**	0.000	0.000	0.000	0.000	0.000	0.000	0.000	0.000	0.000	0.281	0.483	0.198	0.105	0.292	0.022	-0.160	-0.030
**srad_winter**	0.000	0.000	0.000	0.000	0.000	0.000	0.000	0.000	0.000	0.000	0.203	0.081	0.307	0.158	0.096	-0.150	0.005
**t_caco3**	0.000	0.000	0.000	0.000	0.000	0.000	0.000	0.000	0.000	0.000	0.000	0.427	0.144	0.303	0.074	-0.130	-0.020
**t_caso4**	0.000	0.000	0.000	0.000	0.000	0.000	0.000	0.000	0.000	0.000	0.000	0.000	0.057	0.374	0.019	-0.040	-0.050
**t_clay**	0.000	0.000	0.000	0.000	0.000	0.000	0.000	0.000	0.000	0.000	0.000	0.000	0.000	0.201	-0.050	0.185	-0.470
**t_esp**	0.000	0.000	0.000	0.000	0.000	0.000	0.000	0.000	0.000	0.000	0.000	0.000	0.000	0.000	0.030	-0.080	-0.040
**t_gravel**	0.000	0.000	0.000	0.000	0.000	0.000	0.000	0.000	0.000	0.000	0.000	0.000	0.000	0.000	0.000	-0.130	0.078
**t_oc**	0.000	0.000	0.000	0.000	0.000	0.000	0.000	0.000	0.000	0.000	0.000	0.000	0.000	0.000	0.000	0.000	-0.190
**t_sand**	0.000	0.000	0.000	0.000	0.000	0.000	0.000	0.000	0.000	0.000	0.000	0.000	0.000	0.000	0.000	0.000	0.000

Table S4 TSS values of individual models in Biomod2

Models	727 pseudo-absence points	1000 pseudo-absence points	5000 pseudo-absence points	10000 pseudo-absence points
**GLM**	0.903	0.894	0.898	0.906
**GBM**	0.931	0.919	0.925	0.933
**GAM**	0.933	0.911	0.940	0.941
**CTA**	0.902	0.897	0.908	0.913
**ANN**	0.874	0.855	0.881	0.805
**SRE**	0.622	0.611	0.627	0.619
**FDA**	0.917	0.901	0.907	0.907
**MARS**	0.928	0.914	0.910	0.934
**RF**	0.944	0.937	0.942	0.946
**MAXENT**	0.475	0.617	0.886	0.904
**Average value of TSS**	0.843	0.845	0.882	0.881

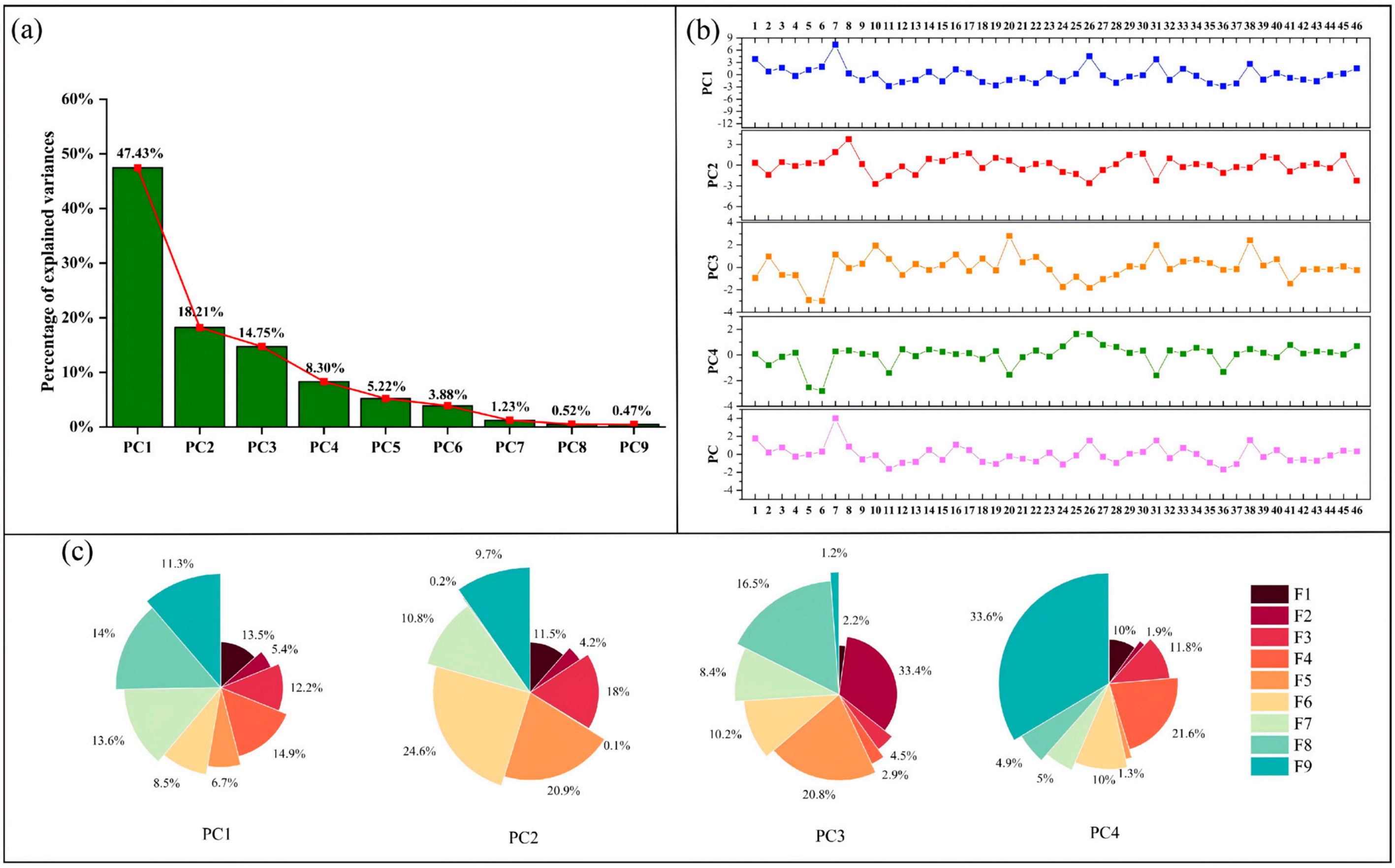

Figure S1 Results of principal component analysis (PCA).

Table S5 Ranking of environmental factors based on XGBoost-SHAP analysis.

pc		Schisandrol A		Gomisin A		Angeloylgomisin H		Schizandrin B		Schisantherin B		Schizandrin A	
**feature**	**importance**	**feature**	**importance**	**feature**	**importance**	**feature**	**importance**	**feature**	**importance**	**feature**	**importance**	**feature**	**importance**
**bio03**	38.083	**bio10**	96.689	**bio03**	49.201	**t_ph_H_2O**	31.665	**srad_winter**	87.338	**bio08**	23.226	**bio01**	52.442
**bio08**	21.474	**srad_winter**	59.598	**bio05**	29.181	**bio12**	17.618	**t_sand**	69.733	**bio17**	8.728	**srad_summer**	28.753
**srad_summer**	15.166	**srad_summer**	54.023	**bio13**	17.165	**t_cec_clay**	12.969	**bio03**	58.009	**bio18**	7.138	**bio08**	22.142
**bio09**	11.421	**bio14**	53.857	**bio04**	16.408	**bio04**	11.083	**bio02**	55.486	**bio05**	5.797	**t_oc**	20.030
**bio02**	9.618	**bio03**	51.084	**bio02**	15.164	**bio02**	7.699	**bio04**	23.757	**bio14**	4.375	**bio05**	12.257
**bio14**	9.467	**bio13**	45.667	**bio14**	11.813	**bio15**	7.479	**bio10**	22.686	**bio02**	2.588	**bio10**	11.082
**bio13**	9.221	**srad_autumn**	43.775	**bio17**	9.968	**srad_summer**	4.810	**bio05**	21.795	**bio01**	2.514	**bio09**	8.390
**bio07**	8.496	**bio01**	36.163	**bio01**	9.647	**bio14**	3.865	**bio17**	16.875	**bio10**	2.486	**bio07**	8.335
**bio04**	8.071	**t_gravel**	14.634	**srad_spring**	6.432	**bio01**	3.534	**bio12**	15.752	**bio19**	1.561	**bio13**	7.254
**bio12**	6.578	**bio02**	13.067	**srad_winter**	5.861	**bio03**	3.431	**bio08**	14.729	**bio15**	1.356	**t_ph_H_2O**	5.781
**bio06**	5.513	**bio06**	11.897	**bio09**	5.354	**srad_winter**	3.417	**srad_autumn**	14.357	**bio13**	1.343	**bio14**	4.613
**bio05**	4.684	**bio16**	10.697	**bio07**	3.570	**srad_spring**	2.400	**srad_summer**	10.619	**bio03**	1.151	**bio02**	4.580
**bio01**	3.877	**bio19**	10.345	**bio10**	3.020	**t_sand**	2.381	**t_cec_soil**	10.143	**bio06**	0.910	**bio03**	2.794
**bio16**	1.927	**bio04**	9.390	**bio15**	1.928	**bio05**	1.743	**bio01**	8.759	**srad_autumn**	0.689	**bio19**	2.335
**bio17**	1.522	**bio17**	8.532	**t_ph_H_2O**	1.516	**srad_autumn**	1.570	**bio16**	6.805	**t_cec_clay**	0.538	**bio17**	0.814
**bio19**	1.082	**bio15**	6.555	**t_silt**	0.990	**bio08**	1.183	**bio15**	6.282	**bio12**	0.436	**srad_spring**	0.522
**bio15**	0.689	**bio05**	4.315	**bio16**	0.336	**bio09**	1.142	**bio13**	4.661	**bio16**	0.296	**t_cec_clay**	0.260

**srad_winter**	0.363	**srad_spring**	2.262	**t_clay**	0.121	**bio06**	0.565	**bio14**	0.527	**srad_summer**	0.148	**bio16**	0.048
**srad_spring**	0.346	**bio12**	1.370	**bio12**	0.081	**bio07**	0.317	**bio06**	0.411	**bio09**	0.064	**bio04**	0.028
**bio10**	0.095	**bio09**	0.624	**bio08**	0.066	**t_esp**	0.290	**srad_spring**	0.350	**t_ph_H_2O**	0.006	**bio18**	0.006
**srad_autumn**	0.013	**t_clay**	0.535	**srad_autumn**	0.034	**bio17**	0.162	**bio18**	0.039	**srad_winter**	0.004	**bio15**	0.002
**t_esp**	0.008	**bio08**	0.203	**t_gravel**	0.018	**bio13**	0.017	**bio07**	0.000	**srad_spring**	0.004	**bio06**	0.000
**bio11**	0.000	**t_ph_H_2O**	0.195	**t_cec_soil**	0.014	**t_oc**	0.009	**bio09**	0.000	**bio04**	0.003	**bio11**	0.000
**bio18**	0.000	**bio07**	0.087	**t_cec_clay**	0.013	**t_silt**	0.007	**bio11**	0.000	**bio07**	0.000	**bio12**	0.000
**t_caco3**	0.000	**bio18**	0.002	**bio18**	0.008	**bio16**	0.001	**bio19**	0.000	**bio11**	0.000	**srad_autumn**	0.000
**t_cec_clay**	0.000	**t_cec_soil**	0.001	**bio19**	0.008	**bio10**	0.000	**t_caco3**	0.000	**t_caco3**	0.000	**srad_winter**	0.000
**t_cec_soil**	0.000	**t_cec_clay**	0.000	**bio06**	0.000	**bio11**	0.000	**t_cec_clay**	0.000	**t_cec_soil**	0.000	**t_caco3**	0.000
**t_clay**	0.000	**bio11**	0.000	**bio11**	0.000	**bio18**	0.000	**t_clay**	0.000	**t_clay**	0.000	**t_cec_soil**	0.000
**t_ece**	0.000	**t_caco3**	0.000	**srad_summer**	0.000	**bio19**	0.000	**t_ece**	0.000	**t_ece**	0.000	**t_clay**	0.000
**t_gravel**	0.000	**t_ece**	0.000	**t_caco3**	0.000	**t_caco3**	0.000	**t_esp**	0.000	**t_esp**	0.000	**t_ece**	0.000
**t_oc**	0.000	**t_esp**	0.000	**t_ece**	0.000	**t_cec_soil**	0.000	**t_gravel**	0.000	**t_gravel**	0.000	**t_esp**	0.000
**t_ph_H_2O**	0.000	**t_oc**	0.000	**t_esp**	0.000	**t_clay**	0.000	**t_oc**	0.000	**t_oc**	0.000	**t_gravel**	0.000
**t_sand**	0.000	**t_sand**	0.000	**t_oc**	0.000	**t_ece**	0.000	**t_ph_H_2O**	0.000	**t_sand**	0.000	**t_sand**	0.000
**t_silt**	0.000	**t_silt**	0.000	**t_sand**	0.000	**t_gravel**	0.000	**t_silt**	0.000	**t_silt**	0.000	**t_silt**	0.000

Table S6 Environmental factors in full subset regression.

pc	Schisandrol A	Gomisin A	Angeloylgomisin H	Schisandrin B	Schisantherin B	Schizandrin A
bio03	bio10	bio03	t_ph_H_2O	srad_winter	bio08	bio01
bio08	srad_winter	bio05	bio12	t_sand	bio17	srad_summer
srad_summer	srad_summer	bio13	t_cec_clay	bio02	bio18	bio08
bio09	bio14	bio02	bio04	bio10	bio05	t_oc
bio02	bio13	bio14	bio02	bio17	bio02	bio07
bio14	t_gravel	srad_spring	bio15	bio12	bio15	bio13
bio13	bio02	bio15	srad_summer	bio08	bio13	t_ph_H_2O
bio19	bio19	t_ph_H_2O	bio14	srad_summer	bio03	bio14
bio15	bio15	t_silt	srad_spring	t_cec_soil	srad_autumn	bio02
srad_spring	t_clay	t_clay	t_sand	bio15	t_cec_clay	bio19
t_esp	bio08	bio08	bio05		srad_summer	srad_spring
	t_ph_H_2O	t_gravel	bio08		t_ph_H_2O	t_cec_clay
	bio18	t_cec_soil	t_esp			bio18
	t_cec_soil	t_cec_clay	t_oc			bio15
	t_cec_clay	bio18	t_silt			
		bio19				

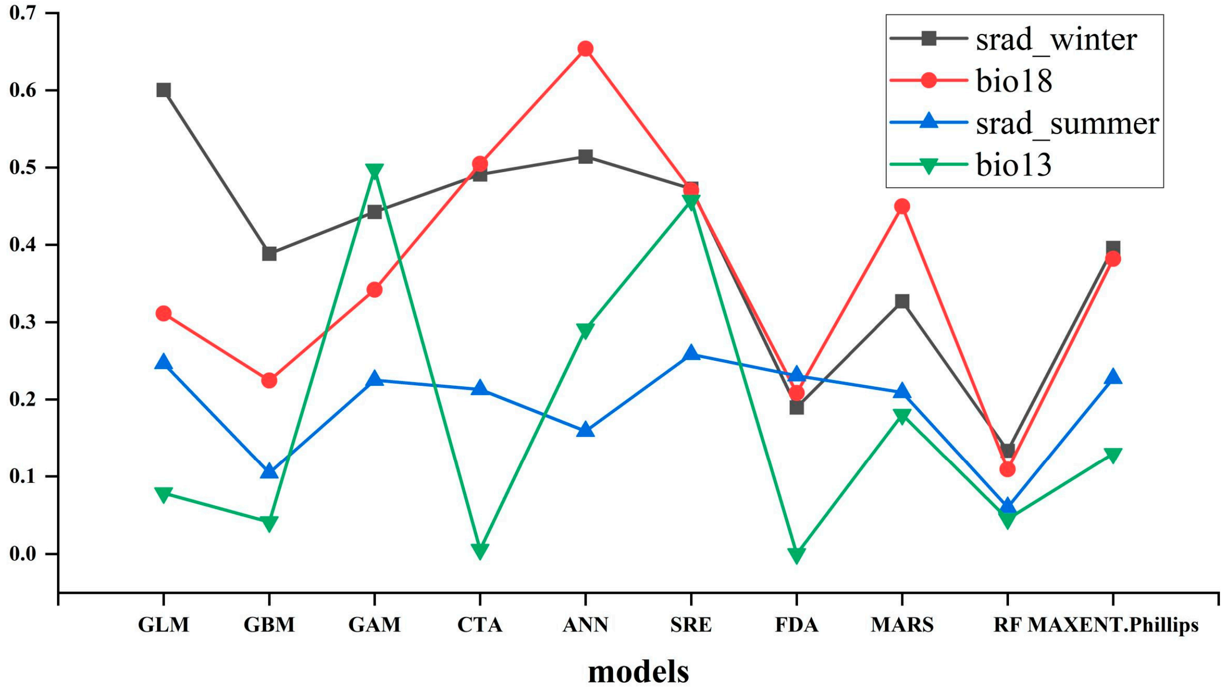

Figure S2 Model assessment results of the main environmental factors

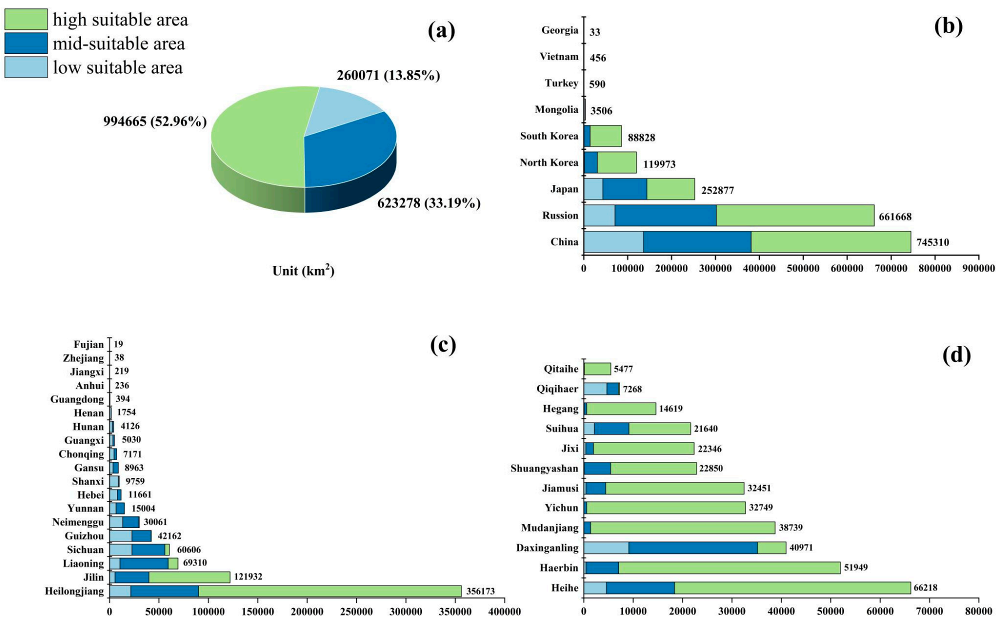

Figure S3 (a)The area of each suitable habitat for *S. chinensis* in the full ecological zone (Asia); (b)The suitable habitat areas for various grades of *S. chinensis* in Asian countries; (c)The suitable habitat areas for various grades of *S. chinensis* in Chinese provinces; (d)The suitable habitat areas for various grades of *S.*

chinensis in cities of Heilongjiang Province.

Table S7 Average values of main environmental factors, 95th percentile, minimum value and maximum value.

**environmental factors**	**bio13**	**bio18**	**srad_summer**	**srad_winter**
**unit**	mm	mm	$kJ \cdot m^{-2} \cdot day^{-1}$	$kJ \cdot m^{-2} \cdot day^{-1}$
**mean value**	203.543	430.18	17986.449	7111.68
**95th percentile**	373.757	731.933	19529.05	9160
**minimum value**	118.458	218.819	14746	4374
**maximum value**	611.194	1032.41	19921	10109

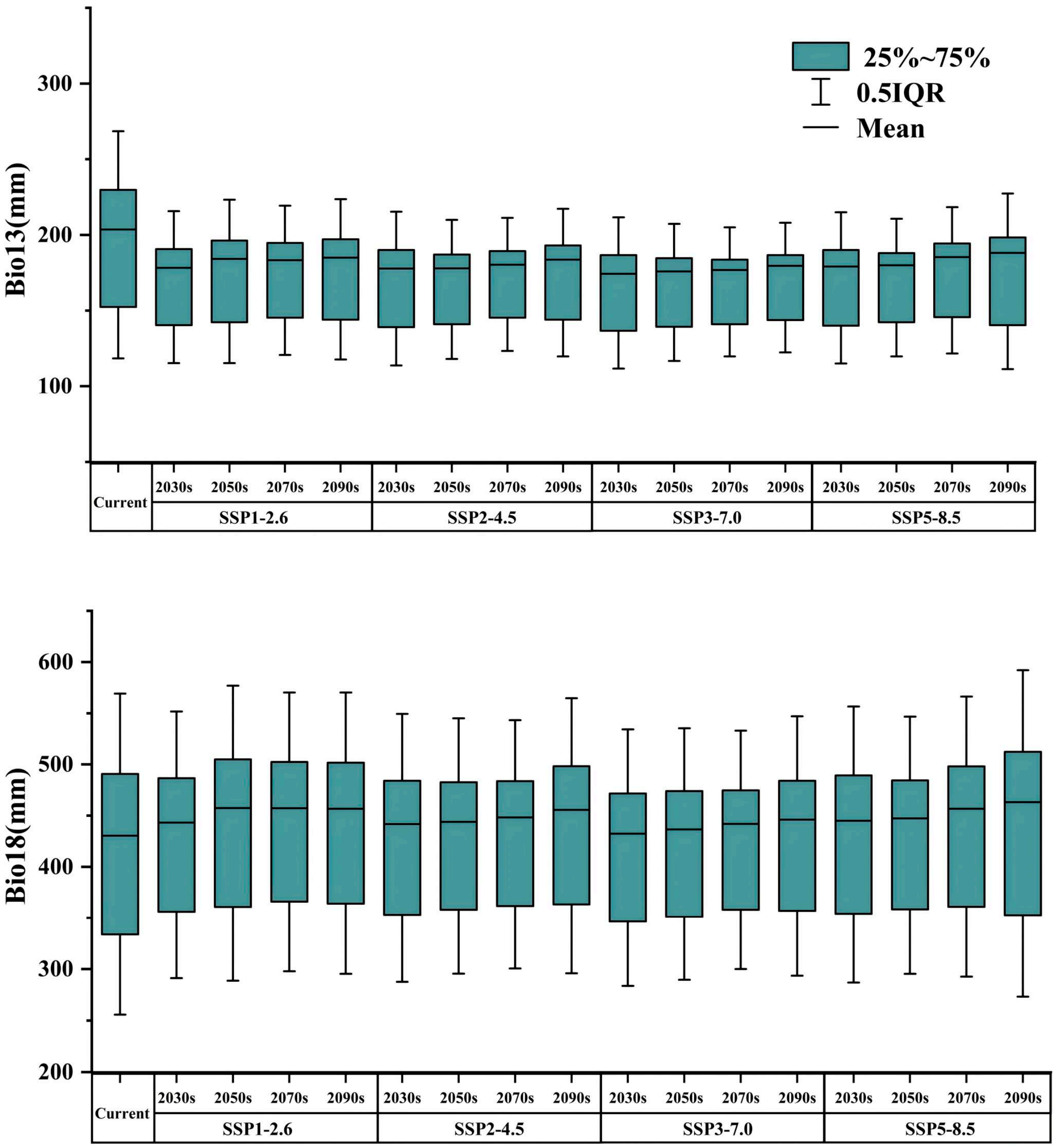

Figure S4 Future changes in key environmental factors.

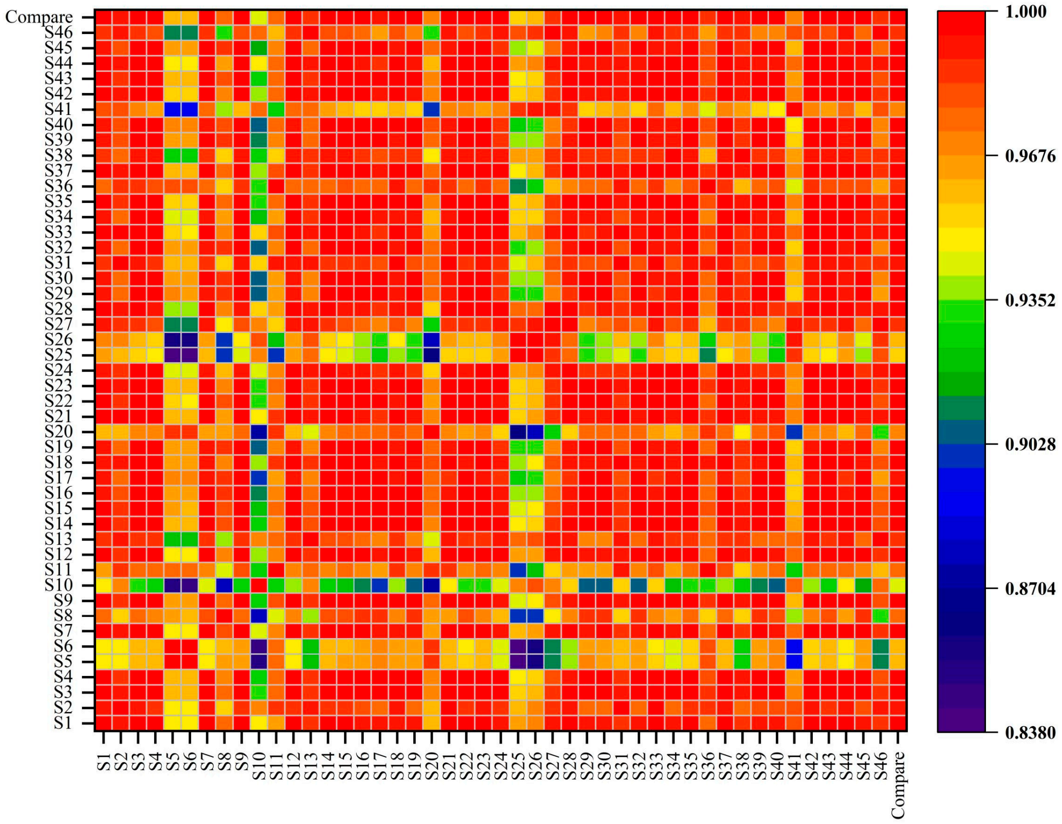

Figure S5 Similarity of *S. Chinensis* from 46 Batches in Heilongjiang Province

Table S8 Changes in the suitable habitat of *S. chinensis* in Heilongjiang Province under different future shared socioeconomic pathway scenarios

	**2030s**	**2050s**	**2070s**	**2090s**
**SSP1-2.6**	12.385%	4.357%	11.600%	-0.616%
**SSP2-4.5**	9.914%	4.827%	-3.594%	-12.154%
**SSP3-7.0**	11.670%	14.531%	-4.019%	-40.808%
**SSP5-8.5**	4.357%	-7.699%	-45.075%	-79.529%

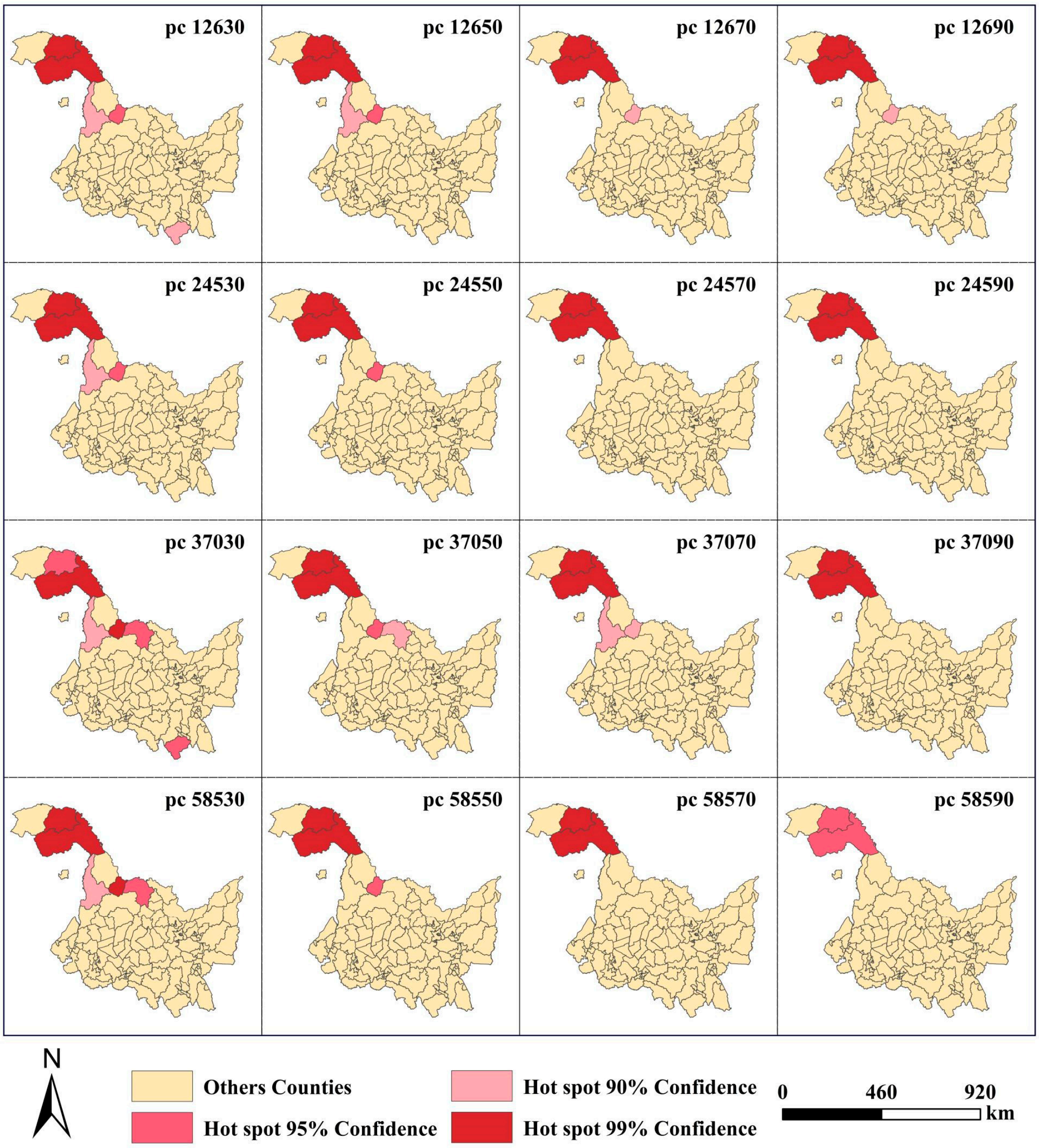

Figure S6 Distribution of high-quality comprehensive constituents (pc) hotspot counties for wild tending of *S. chinensis* in Heilongjiang Province under future global change.

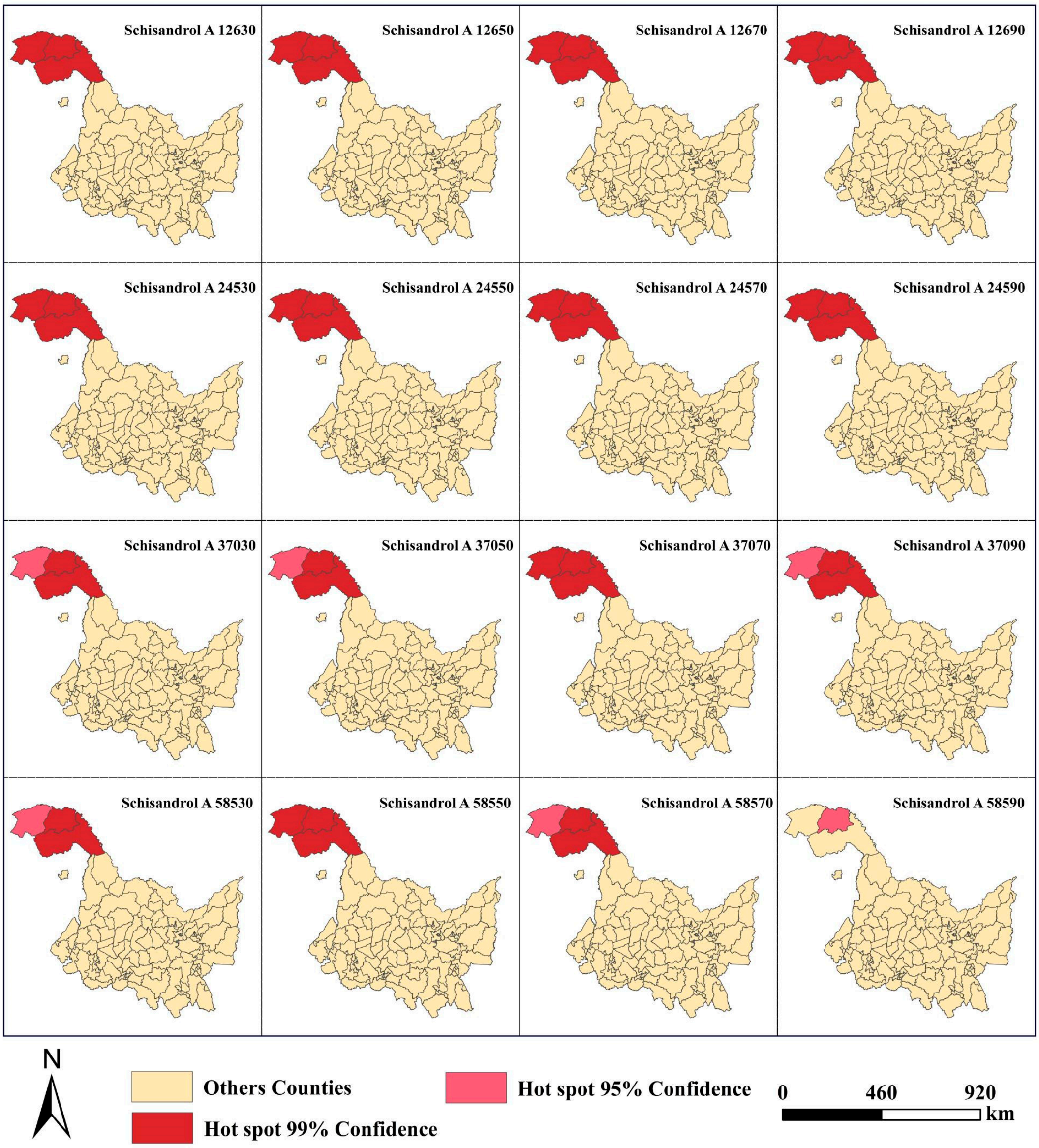

Figure S7 Distribution of high-quality Schisandrol A hotspot counties for wild tending of *S. chinensis* in Heilongjiang Province under future global change.

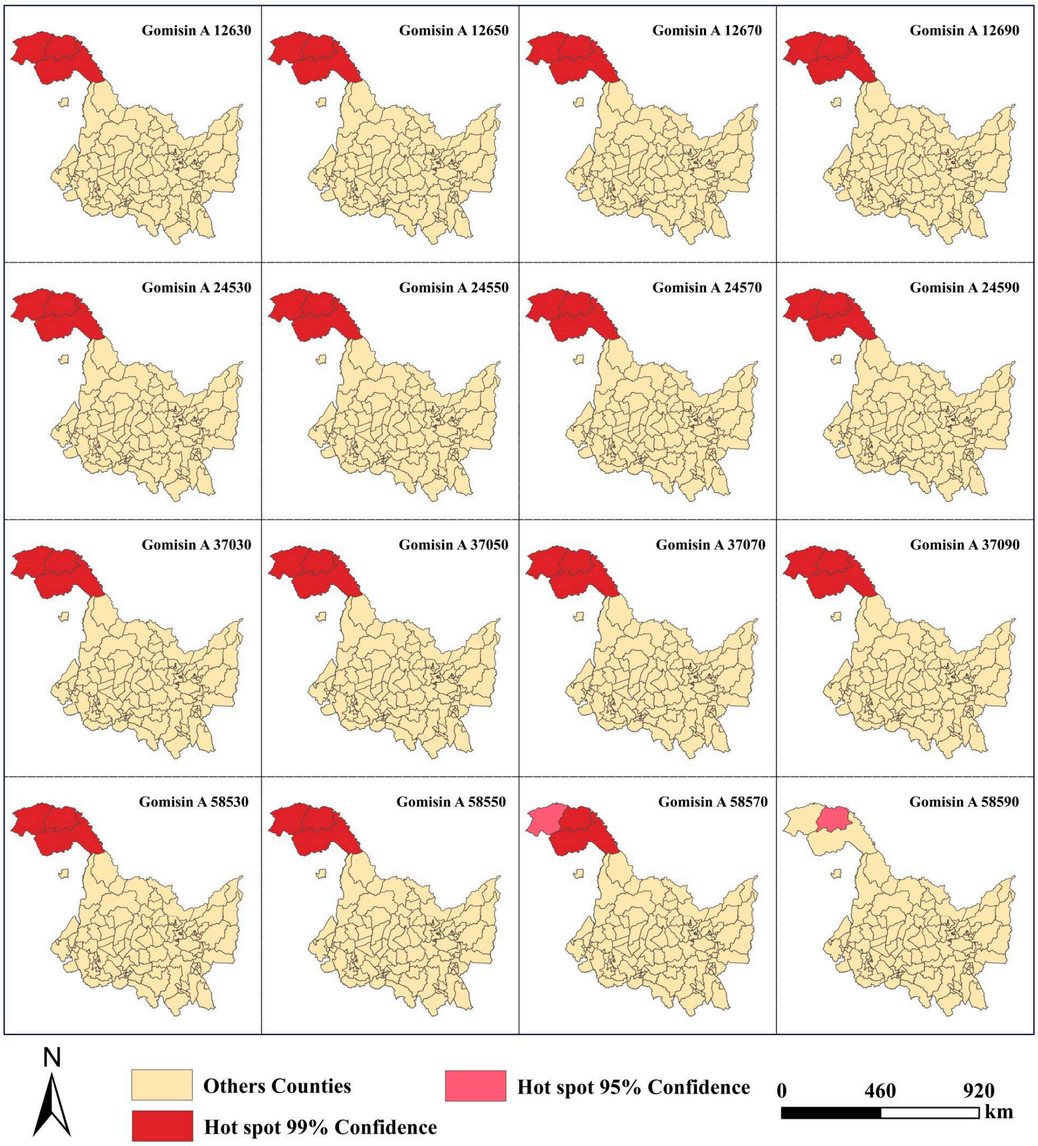

Figure S8 Distribution of high-quality Gomisin A hotspot counties for wild tending of *S. chinensis* in Heilongjiang Province under future global change.

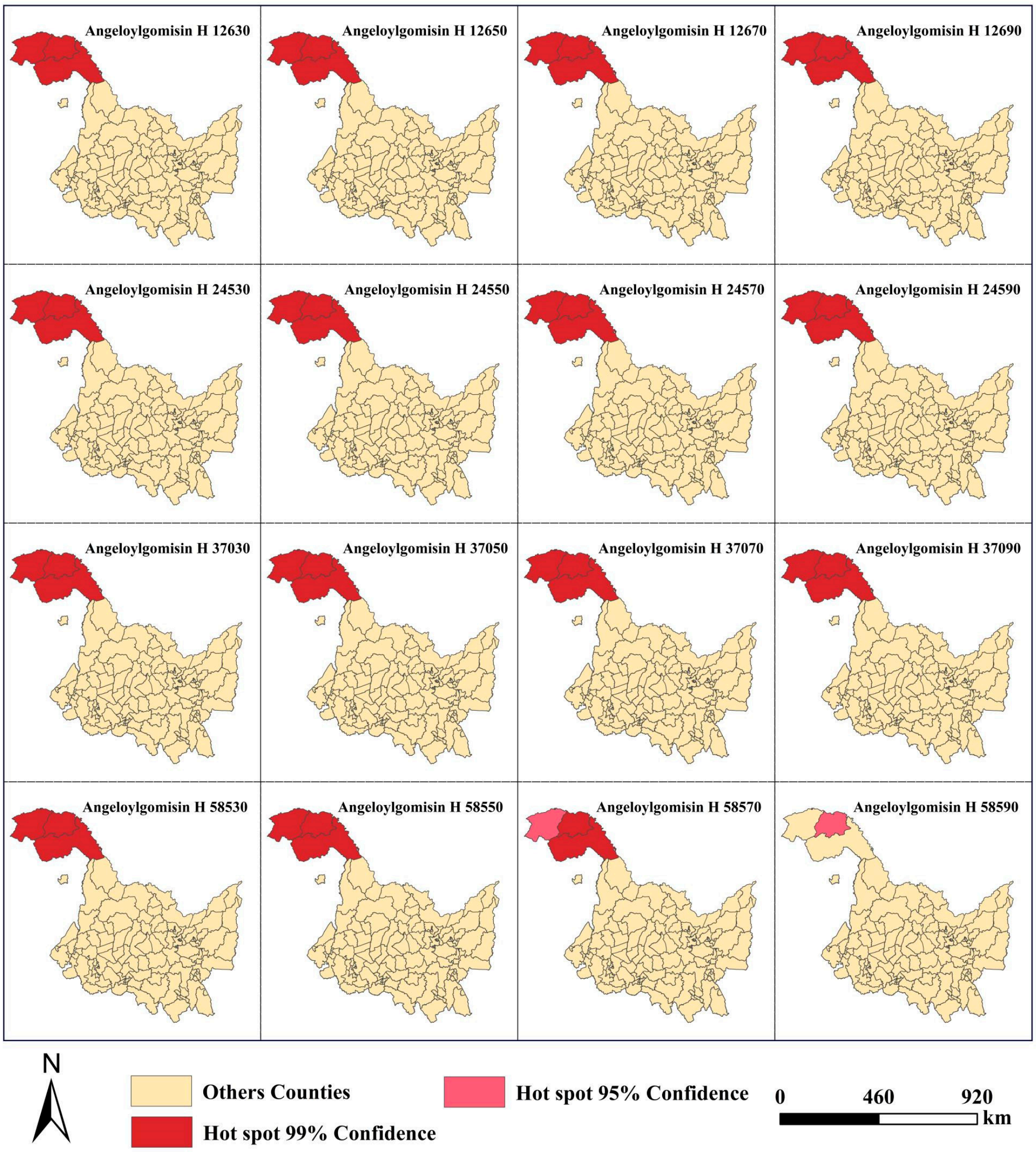

Figure S9 Distribution of high-quality Angeloylgomisin H hotspot counties for wild tending of *S. chinensis* in Heilongjiang Province under future global change.

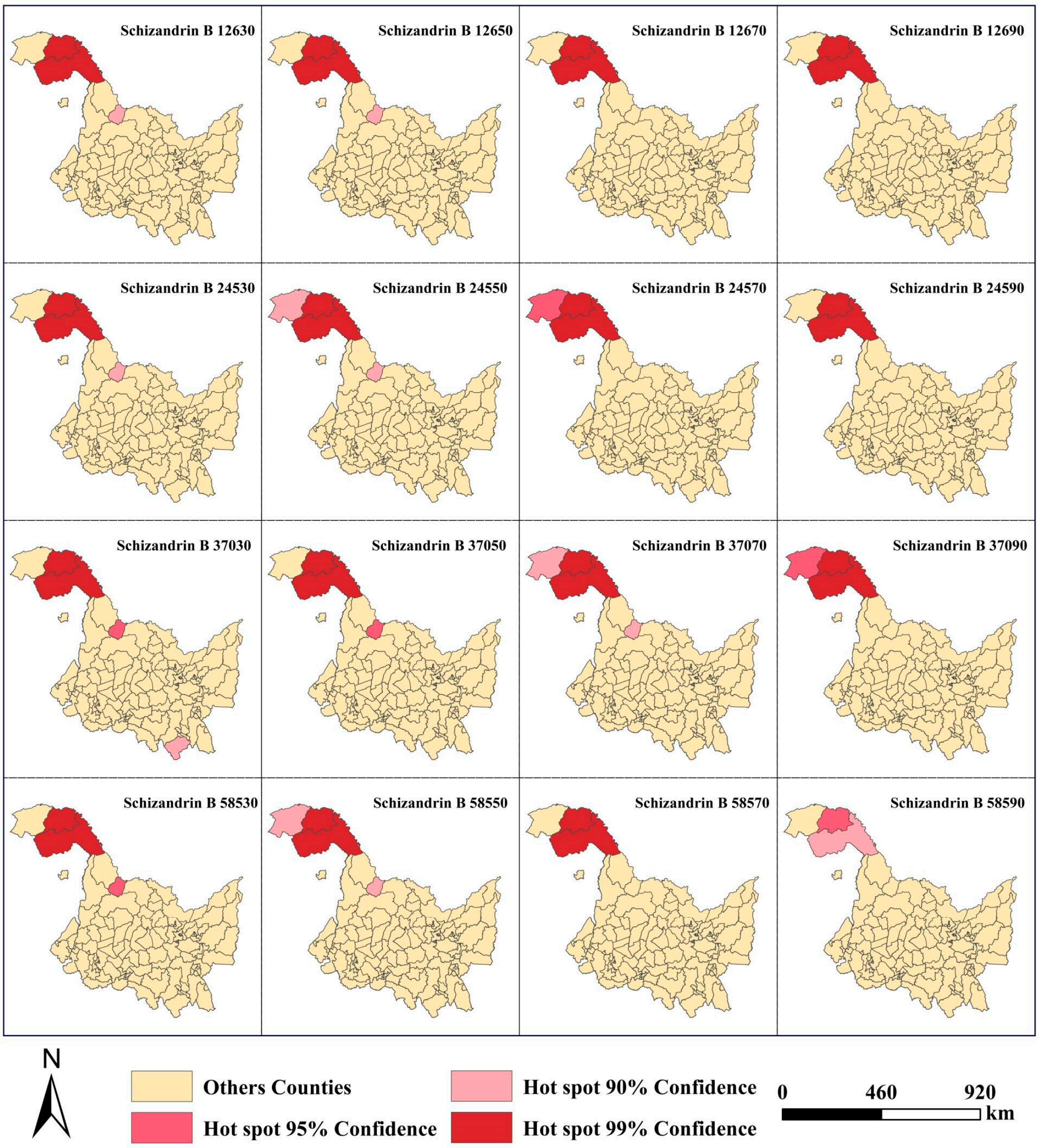

Figure S10 Distribution of high-quality Schizandrin B hotspot counties for wild tending of *S. chinensis* in Heilongjiang Province under future global change.

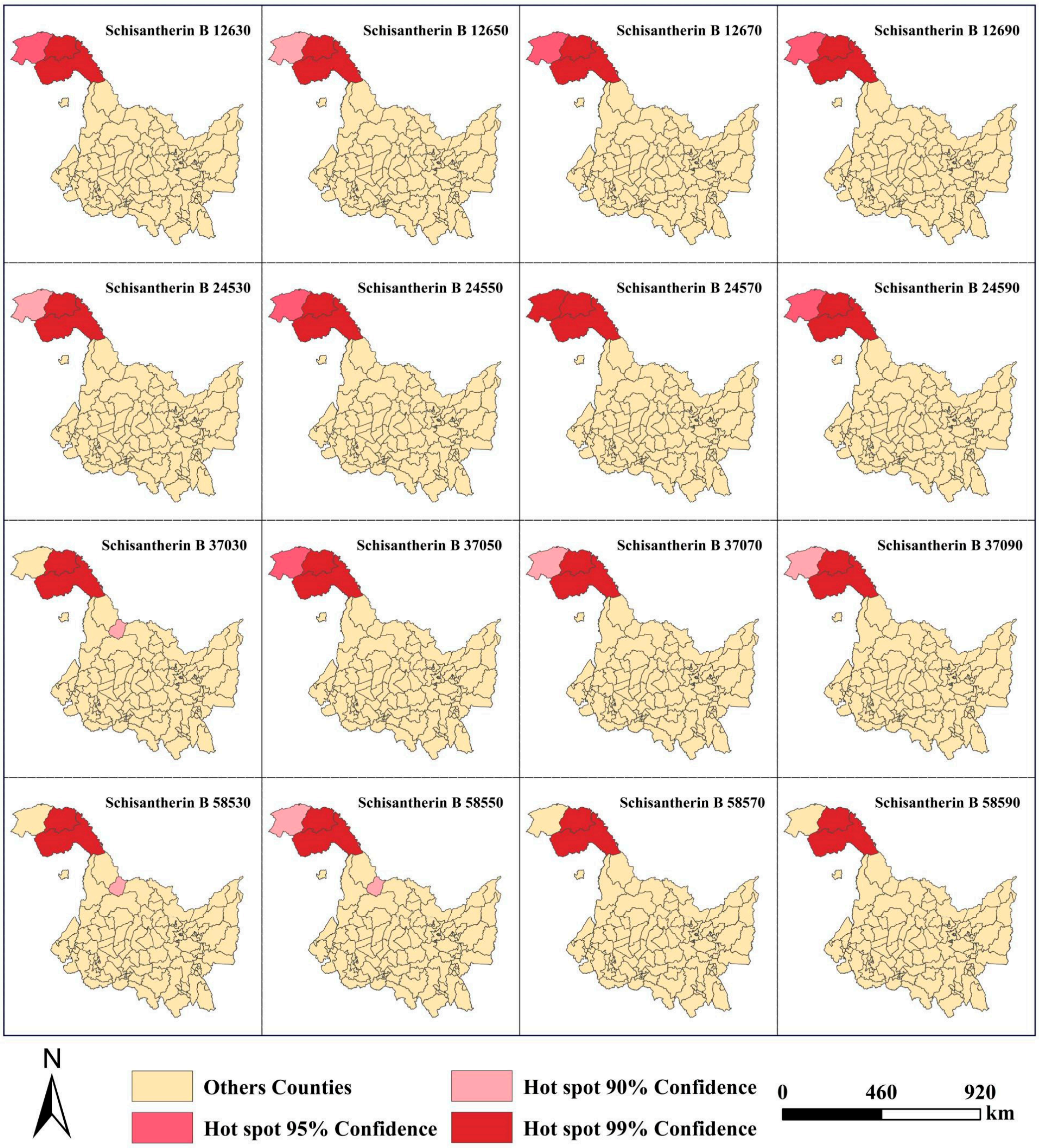

Figure S11 Distribution of high-quality Schisantherin B hotspot counties for wild tending of *S. chinensis* in Heilongjiang Province under future global change.

Supporting Information of The Chromatographic Fingerprint:

1. Methods

Sample: *Schisandra chinensis*: Information for 46 batches of samples is shown in Table M1-1.

Table M1-1 Sample information.

Serial Number	Batch Number	Collection Site	Latitude	Longtitude	collection periods	phenological stages
1	SC-TH-57	Tahe	52.666941	123.833463	2025.9.02	fructescence
2	SC-HM-58	Huma	51.806817	126.465251	2025.9.02	fructescence
3	SC-JGDQ-59	Jiagedaqi	52.526371	121.867551	2025.9.03	fructescence
4	SC-TH-20	Tonghe	46.428629	128.228839	2025.9.11	fructescence
5	SC-BY-23	Bayan	46.247914	127.540669	2025.9.11	fructescence
6	SC-WC-24	wuchang	44.365779	127.953739	2025.9.15	fructescence
7	SC-AC-25	Acheng	45.525182	127.281682	2025.9.15	fructescence
8	SC-YS-26	Yanshou	45.650288	128.278594	2025.9.11	fructescence
9	SC-FZ-28	Fangzheng	45.859092	129.16271	2025.9.12	fructescence
10	SC-WH-30	Weihe	45.133113	128.375818	2025.9.15	fructescence
11	SC-YBL-33	Yabuli	44.845971	128.006407	2025.9.15	fructescence
12	SC-YL-39	Yilan	46.479769	129.320533	2025.9.12	fructescence
13	SC-SZ-42	Shangzhi	45.162968	128.876707	2025.9.15	fructescence
14	SC-LB-46	Luobei	48.020979	130.673683	2025.9.12	fructescence
15	SC-XK-14	Xunke	49.211682	129.133429	2025.9.04	fructescence
16	SC-AH-49	Aihui	50.754072	127.196199	2025.9.04	fructescence
17	SC-SW-51	Sunwu	49.597245	127.56027	2025.9.04	fructescence
18	SC-WDLC-55	Wudalianchi	48.752487	126.470438	2025.9.05	fructescence
19	SC-BA-56	Beian	48.378588	127.364344	2025.9.05	fructescence
20	SC-HS-27	Hengshan	45.313746	130.831976	2025.9.14	fructescence
21	SC-HL-36	Hulin	45.829726	132.35811	2025.9.14	fructescence
22	SC-MS-43	Mishan	45.728367	131.963439	2025.9.14	fructescence
23	SC-LS-60	Lishu	45.013278	130.709146	2025.9.14	fructescence
24	SC-HN-22	Huanan	46.479445	130.77525	2025.9.12	fructescence
25	SC-TY-38	Tangyuan	47.109824	130.011942	2025.9.12	fructescence
26	SC-ML-34	Muling	44.450364	130.409818	2025.9.16	fructescence
27	SC-LK-37	Linkou	45.566837	129.598999	2025.9.16	fructescence
28	SC-DN-40	Dongning	44.04663	130.535778	2025.9.16	fructescence
29	SC-SFH-41	Suifenhe	44.305708	131.060922	2025.9.16	fructescence
30	SC-HL-48	Hailin	44.970344	129.511994	2025.9.16	fructescence
31	SC-NA-52	Ningan	44.034796	129.662667	2025.9.16	fructescence
32	SC-BL-31	Boli	45.66782	130.850629	2025.9.10	fructescence
33	SC-QTH-32	Qitaihe	45.647598	131.247449	2025.9.10	fructescence
34	SC-RH-21	Raohe	47.092622	134.041314	2025.9.13	fructescence

35	SC-BQ-35	Baoqing	46.318225	131.989514	2025.9.13	fructescence
36	SC-QA-9	Qingan	47.086509	127.915579	2025.9.06	fructescence
37	SC-BL-15	Beilin	46.933607	127.122353	2025.9.06	fructescence
38	SC-SL-16	Suiling	47.850691	128.23909	2025.9.06	fructescence
39	SC-YH-1	Youhao	48.264169	128.553343	2025.9.07	fructescence
40	SC-WY-3	Wuying	48.160475	129.175015	2025.9.07	fructescence
41	SC-WMH-4	Wumahe	47.750549	129.092531	2025.9.07	fructescence
42	SC-TL-6	Tieli	46.776604	128.519514	2025.9.09	fructescence
43	SC-TW-7	Tangwang	48.492329	129.68913	2025.9.08	fructescence
44	SC-NC-10	Nancha	47.276142	129.147125	2025.9.09	fructescence
45	SC-JL-13	Jinlin	47.611487	129.696307	2025.9.09	fructescence
46	SC-JY-19	jiayin	48.856103	130.225668	2025.9.08	fructescence

Reagents: Table M1-2,Table M1-3.

Table M1-2 Information on Instruments and Equipment.

Serial number	Name	Model	Manufacturer
1	High-performance liquid chromatograph	E2695/PDA2998	Waters
2	High-performance liquid chromatograph	E2695/UV2489	Waters
3	Chromatographic column	5HC-C18(2)	Agilent
4	Chromatographic column	Atlantis TM T3	Waters
5	Chromatographic column	Inertsil ODS-3 C18	Shimadzu

Table M1-3 Reagent test reagents and reference substance information.

Serial Number	Name	Manufacturer	Batch number	Level
1	Methanol	Dima	LOT:R142743	Chromatography grade
2	Acetonitrile	Dima	LOT:R142750	Chromatography grade
3	Methanol	Zhiyuan	20240701	Analytical Grade
4	Phosphoric Acid	Zhiyuan	20230801424	Analytical Grade
5	Formic acid	Zhiyuan	20230501327	Analytical Grade
6	Ultra-pure water	Self-made	——	——
7	S. *chinensis* Standard Reference Material	China Food and Drug Inspection Institute	120922-202311	——
8	Schisandrol A	China Food and Drug Inspection Institute	110857-202316	——

9	Gomisin A	China Food and Drug Inspection Institute	19055-P240701	——
10	Angeloylgomisin H	Shanghai Shidandai Standard Technical Service	040165-202309	——
11	Schisantherin B	China Food and Drug Inspection Institute	112109-202401	——
12	Schizandrin A	China Food and Drug Inspection Institute	110764-201915	——
13	Schizandrin B	China Food and Drug Inspection Institute	110765-201813	——

1.2 Study on chromatographic conditions

1.2.1 Chromatographic condition research plan

The test sample solution was prepared according to the pretreatment conditions of Scheme 1 to Scheme 5, and the chromatographic conditions of Scheme 1 to Scheme 5 were used for the test. The full wavelength scan was performed with a PDA detector and the chromatogram under specific wavelength conditions was recorded (Table M1-4).

Table M1-4 Initial chromatographic conditions comparison.

Scheme	Mobile phase composition	Elution program	Column temperature/℃	Wave length/nm	Reference
1	Acetonitrile (A) -0.1% phosphoric acid(B） 0.8 mL/min	see table M1-5	30	216	ZhangXia.Study on the Quality Evaluation of *Schisandra Chinensis* Based on HPLC Characteristic Chromatogram and Multi-Test Evaluation[J]. Journal of Chinese Medicinal Materials,2024,07(47):1733-1737.
2	Acetonitrile (A) - aqueous solution(B) 0.8 mL/min	see table M1-5	30	216	ZhangXia.Study on the Quality Evaluation of *Schisandra Chinensis* Based on HPLC Characteristic Chromatogram and Multi-Test Evaluation[J]. Journal of Chinese Medicinal Materials,2024,07(47):1733-1737.
3	Acetonitrile (A) - water(B) 1.0 mL/min	see table M1-6	30	220	Han Zhaofeng. Study on Quality Evaluation of Geo-authentic Traditional Chinese Drug in Liaoning Beiwuweizi(*Schisandrae*

					Chinensis Fructus)Based on Chemometrics and Fingerprint[J]. CHINESE ARCHIVES OF TRADITIONAL CHINESE MEDICINE,2024,09(42):69-73.
4	Acetonitrile (A) - water(B） 1.0 mL/min	see table M1-7	30	220	Liu Zizhen,Establishment of Characteristic Pattern of Vinegar-processed *Schisandra Chinensis* Formula Granules and Content Determination of 5 Components.[J].China Academic Journal Electronic Publishing House,2021,32(12):1453-1459
5	Acetonitrile (A) -0.2% formic acid(B) 0.8 mL/min	see table M1-5	30	216	ZhangXia.Study on the Quality Evaluation of *Schisandra Chinensis* Based on HPLC Characteristic Chromatogram and Multi-Test Evaluation[J]. Journal of Chinese Medicinal Materials,2024,07(47):1733-1737.

Table M1-5 Scheme 1, 2, and 5 gradient elution procedures.

Time/min	Mobile phase A/%	Mobile phase B/%
0	45	55
20	60	40
26	60	40
50	75	25
51	45	55
60	45	55

Table M1-6 Scheme 3 gradient elution procedures.

Time/min	Mobile phase A/%	Mobile phase B/%
0-10	50-45	50-55
10-15	45-42	55-58
15-20	42-45	58-55
20-30	45-50	55-50
30-40	50-60	50-40
40-55	60-70	40-30

Table M1-7 Scheme 4 gradient elution procedures.

Time/min	Mobile phase A/%	Mobile phase B/%
0-20	45-55	55-45
25-30	55-65	45-35
30-45	65-100	35-0

2 Results

2.1 Selection of Chromatographic Protocol

The chromatographic scheme was evaluated and selected from dimensions including peak shape, baseline stability, peak separation efficiency and peak number. As shown in Figure M2-1, for Scheme 2 and Scheme 4, where acetonitrile-water solution was used as the mobile phase, chromatographic peaks were observed, but the number of peaks was small and the peak shape was poor. For Scheme 5, where acetonitrile-0.2% formic acid aqueous solution was used as the mobile phase, chromatographic peaks were detected, but the baseline was unstable and the number of peaks was small. Among all five schemes, Scheme 1 provided more peaks with relatively complete peak information, featured an appropriate gradient elution time, and achieved superior overall resolution.

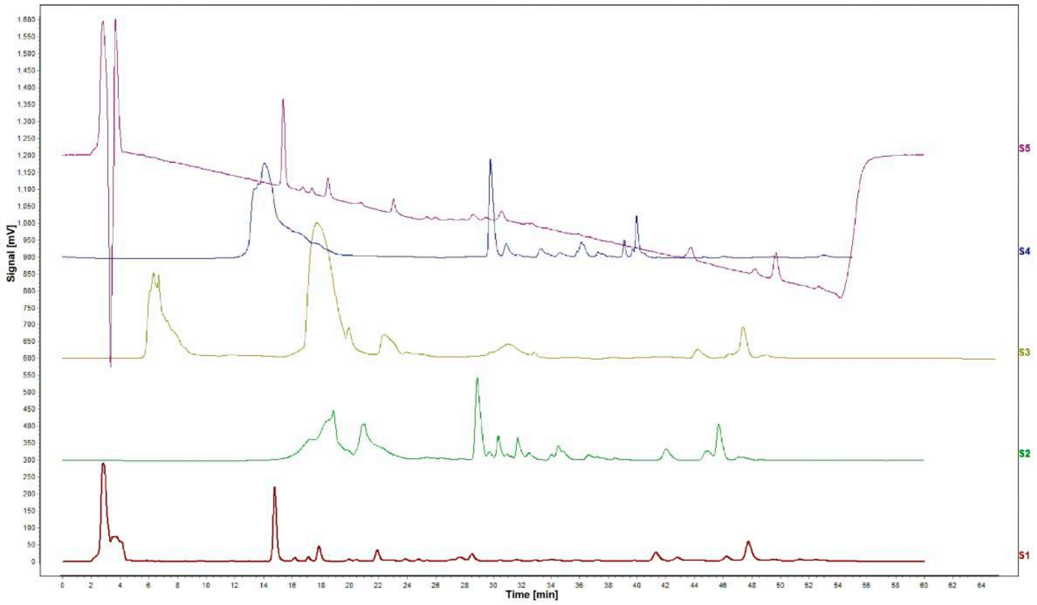

Figure M2-1 Plots of Pre-Experiment Results for Schemes 1 to 5. (S1–S5 correspond to Schemes 1 to 5, respectively)

2.2 Wavelength Selection

According to the full-wavelength scanning results of Scheme 1, the peak information is relatively abundant within the wavelength range of 220 nm ~ 260 nm, and the peak information at 220 nm is completely manifested. Therefore, Scheme 1 is selected as the elution condition for method development.

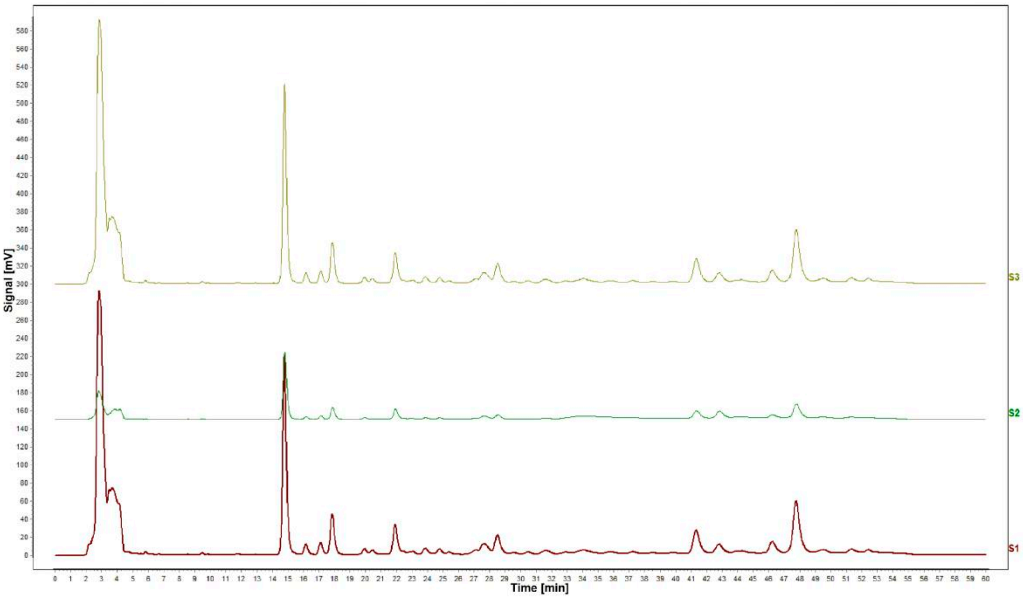

Figure M2-2 Pre-experiment results of Scheme 1 at different wavelengths. (S1–S3 correspond to Scheme 1 at 220 nm, Scheme 1 at 250 nm, and Scheme 1 at 260 nm, respectively)

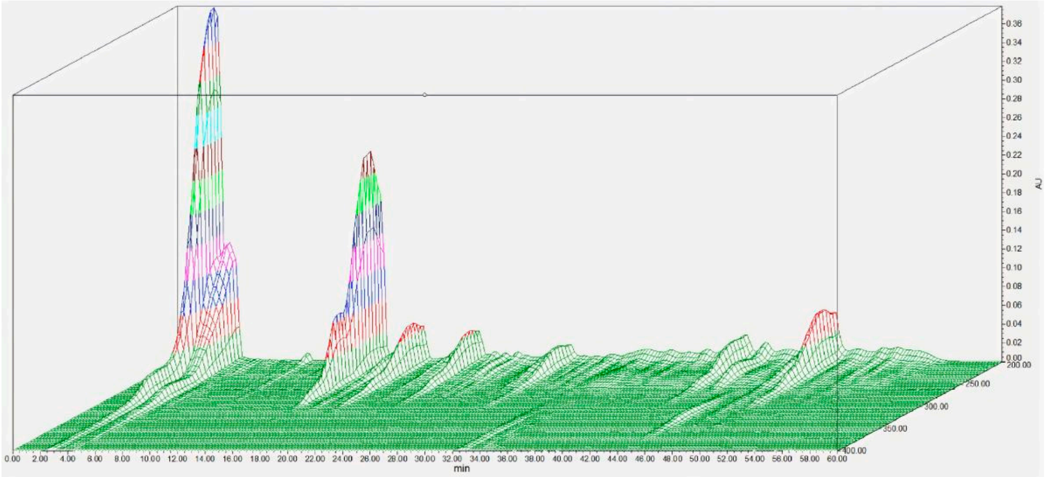

Figure M2-3 Full-wavelength scanning spectrum based on Scheme 1.

2.3 Conclusion

Scheme 1 is selected as the basis for the chromatographic conditions in subsequent experiments, with the detection wavelength set at 220 nm.

3 Reference Substance Localization

3.1 Methodology

Accurately weigh an appropriate amount of the reference standards, namely Schisandrol A, Gomisin A, Angeloylgomisin H, Schisantherin B, Schizandrin A and Schizandrin B, place them in volumetric flasks, add methanol respectively to prepare reference standard solutions with a concentration of 0.1 mg per 1 mL for each component. Select the following 5 batches of test samples, and conduct the test with reference to the chromatographic conditions specified in Section 2.3 to verify the general applicability of the reference spectrum.

Table M3-1 Information of Test Samples for Comparison of Chromatographic Conditions.

Serial Number	Batch Number	Sample Origin
1	SC-TH-57	Heilongjiang Province
2	SC-BY-23	Heilongjiang Province
3	SC-HL-36	Heilongjiang Province
4	SC-TY-38	Heilongjiang Province
5	SC-TL-6	Heilongjiang Province

3.2 Result

According to the results presented in Figure M3-1, the peaks corresponding to Schisandrol A, Gomisin A, Angeloylgomisin H, Schisantherin B, Schizandrin A and Schizandrin B were successfully located and assigned in the test sample.

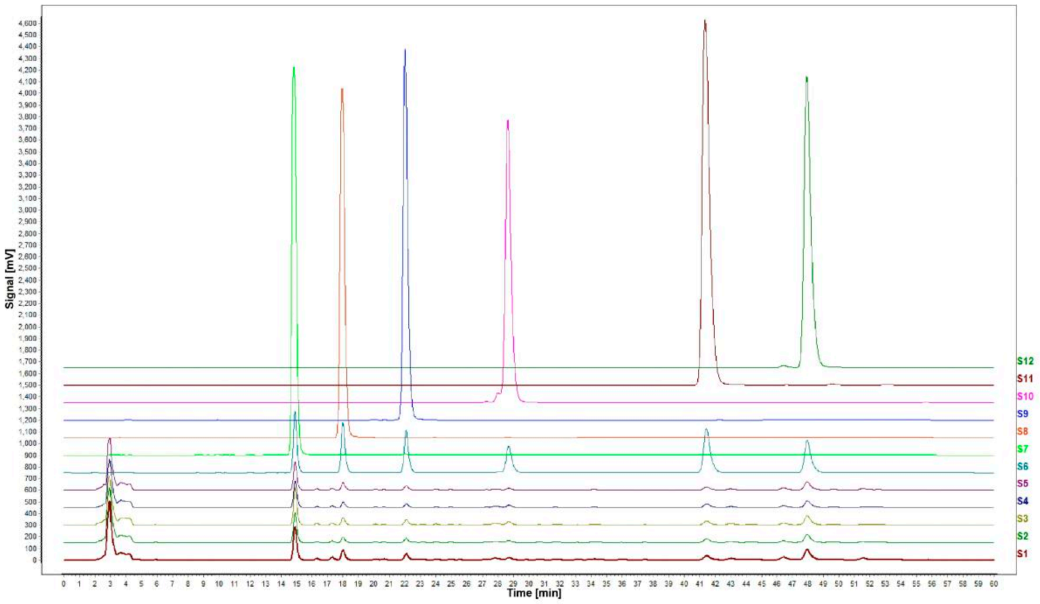

Figure M3-1 Spectra obtained by comparative positioning. (S1–S12 correspond to SC-TH-57, SC-BY-23, SC-HL-36, SC-TY-38, SC-TL-6, mixed reference, Schisandrol A, Gomisin A, Angeloylgomisin H, Schisantherin B, Schizandrin A and Schizandrin B, respectively)

Based on the comparison of peak area between the standard products and the test samples, the concentrations of Schisandrol A, Gomisin A, Angeloylgomisin H, Schisantherin B, Schizandrin A and Schizandrin B were optimized as follows: 40 µg of Schisandrol A, 10 µg each of Gomisin A, Angeloylgomisin H, Schisantherin B and Schizandrin A, and 20 µg of Schizandrin B per 1 mL, with methanol used as the dilution solvent.

As shown in Figure M3-2, the corresponding component chromatographic peaks can be identified at the retention positions corresponding to the standards of Schisandrol A, Gomisin A, Angeloylgomisin H, Schisantherin B, Schizandrin A and Schizandrin B.

The reference standard solution was prepared with methanol as the solvent, which exhibited symmetric peak shape and no solvent effect. The concentrations of Schisandrol A, Gomisin A, Angeloylgomisin H, Schisantherin B, Schizandrin A and Schizandrin B were optimized with reference to the peak area of the test solution.

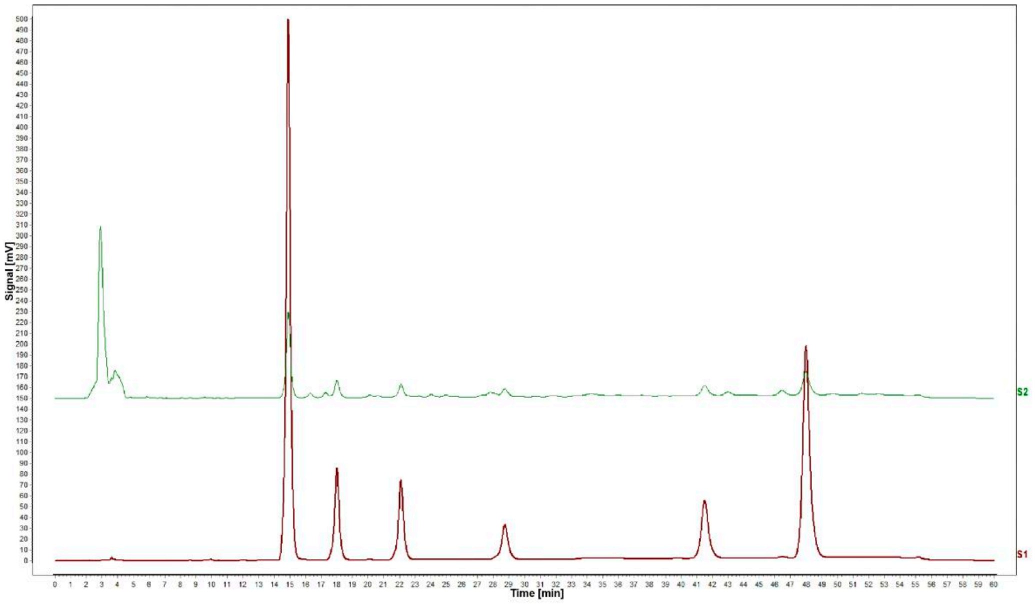

Figure M3-2 Optimization chromatogram of reference solution concentration. (S1-S2 correspond to mixed reference and SC-HL-36, respectively)

3.3 Conclusion

Under this chromatographic method, the chromatographic peaks of Schisandrol A, Gomisin A, Angeloylgomisin H, Schisantherin B, Schizandrin A and Schizandrin B can be successfully identified. The reference substance solution is a mixed solution with the following concentrations per 1 mL: 40 μg/mL of Schisandrol A, 10 μg/mL each of Gomisin A, Angeloylgomisin H, Schisantherin B and Schizandrin A, and 20 μg/mL of Schizandrin B.

4 Preparation of Test Samples

4.1 Investigation of Extraction Solvent

4.1.1 Methods

A single batch of *S. chinensis* (SC-HL-36) was selected as the test sample. Taking the number of chromatographic peaks and the degree of separation as evaluation indicators, the effects of different extraction solvents on the experimental results were compared. The investigated factors were 50% methanol, 80% methanol and methanol, respectively.

Preparation of Test Solution: Accurately weigh approximately 0.5 g of *S. chinensis* Fructus powder (passed through a No. 4 sieve), transfer it into a conical flask with a stopper, precisely add 50 mL of the corresponding solvent, stopper the flask tightly, weigh the assembly, heat under reflux for extraction for 30 minutes, allow it to cool to room temperature, weigh again, replenish the mass loss with the corresponding solvent, shake well, filter, collect the subsequent filtrate and pass it through a 0.45 μm microporous membrane. The resulting filtrate is the test solution.

4.1.2 Results

According to the spectral atlas analysis, the chromatographic separation resolution and the theoretical plate

number obtained when using 50% methanol, 80% methanol, and pure methanol as extraction solvents are comparable. A comparison of the peak area per unit mass shows that 80% methanol achieves a better extraction effect. Therefore, 80% methanol is selected as the extraction solvent.

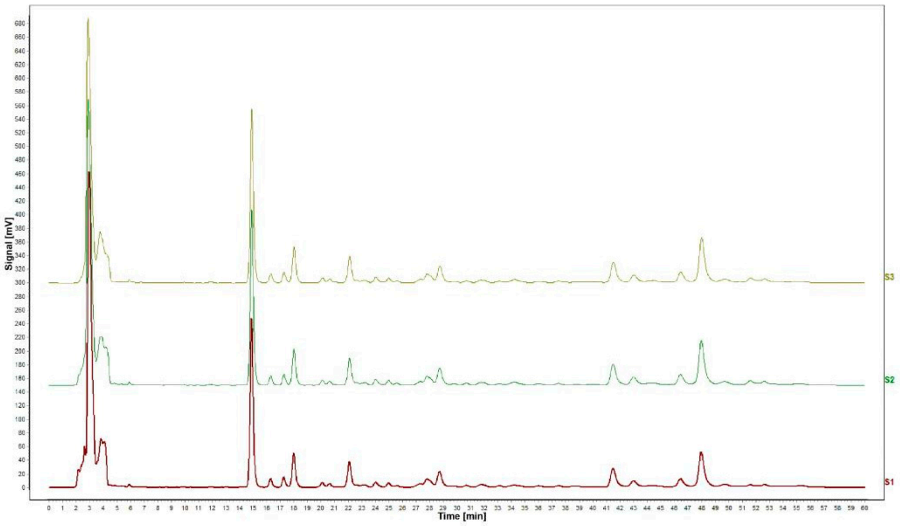

Figure M4-1 Chromatograms obtained from the investigation of extraction solvents. (S1-S3 correspond to 50% methanol, 80% methanol and methanol, respectively)

Table M4-1 Comparison of peak area and peak area per unit mass for different extraction solvents.

Comparison of Peak Area for Different Extraction Solvents				Comparison of Peak Area per Unit Mass for Different Extraction Solvents			
	50% Methanol	80% Methanol	Methanol		50% Methanol	80% Methanol	Methanol
Peak 1	3977284	4176235	4165585	Peak 1	7548461	7911034	7688418
Peak 2	863887	912747	909507	Peak 2	1639565	1729015	1678677
Peak 3	719512	759529	757584	Peak 3	1365557	1438774	1398272
Peak 4	449052	470849	466781	Peak 4	852253	891928	861537
Peak 5	760422	839033	836709	Peak 5	1443200	1589379	1544313
Peak 6	1466162	1873836	1884653	Peak 6	2782619	3549604	3478503

4.1.3 Experimental Conclusion

80% methanol is selected as the extraction solvent.

4.2.1 Methods

Based on the extraction solvent determined above, this section investigates the differences in extraction outcomes obtained via different methods and durations, and selects the optimal extraction method. The ultrasonic extraction durations were set to 20 min, 30 min and 45 min respectively, while the reflux extraction durations were set to 30 min, 45 min and 60 min respectively. The peak area per unit mass of the main chromatographic peak was adopted as the primary evaluation indicator to select the appropriate extraction method and duration.

Take approximately 0.5 g of *S. chinensis* powder (sieved through a No. 4 sieve), weigh it accurately, place it into a conical flask with a stopper, accurately add 50 mL of 80% methanol, stopper the flask tightly, weigh it,

extract with different extraction methods, allow it to cool to room temperature, weigh it again, compensate for the lost mass with 80% methanol, shake well, filter, take the subsequent filtrate and pass it through a 0.45 μm microporous filter membrane. The test solution is thus obtained.

4.2.2 Results

The six schemes exhibit comparable chromatographic resolution and theoretical plate numbers. Comparison of peak area per unit mass indicates that 30-minute ultrasonic extraction achieves better extraction performance. Therefore, 30-minute ultrasonic treatment is selected as the extraction method and duration.

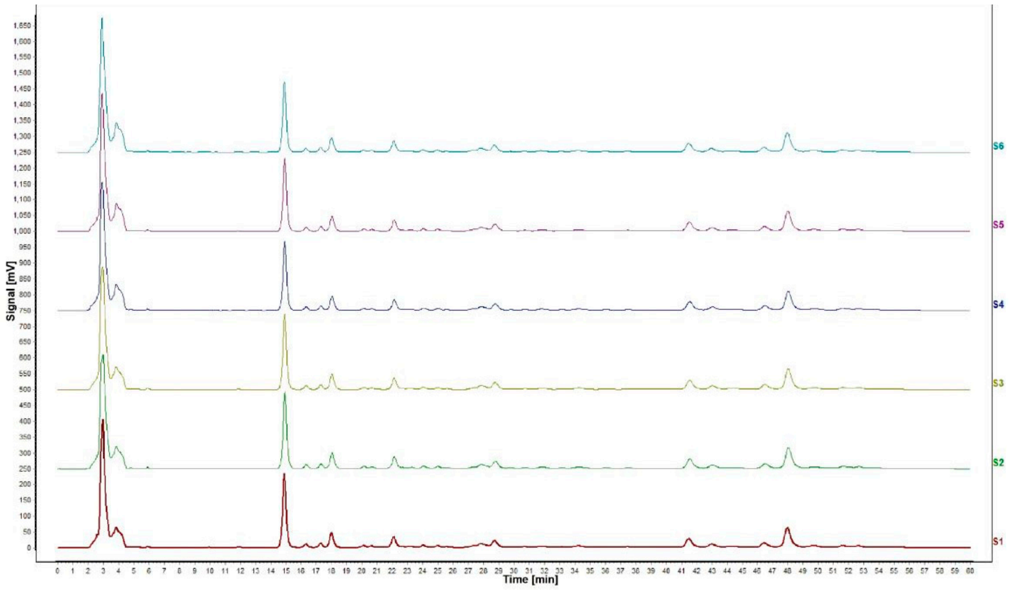

Figure M4-2 Chromatograms obtained under different extraction times. (S1 to S6 correspond to 20-min ultrasonication, 30-min ultrasonication, 45-min ultrasonication, 30-min reflux extraction, 45-min reflux extraction and 60-min reflux extraction, respectively)

Table M4-2 Comparison of Peak Area Obtained by Different Extraction Methods and Extraction Durations.

	Ultrasound 20 min	Ultrasound 30 min	Ultrasound 45 min	Reflux 30 min	Reflux 45 min	Reflux 60 min
Peak 1	4108653	4320520	4225083	3892738	4090750	3934161
Peak 2	867803	909369	891377	819688	865910	836132
Peak 3	663829	698150	680923	627319	658287	631607
Peak 4	427404	450265	438236	404694	430670	414845
Peak 5	823404	868212	847353	782743	818881	787571
Peak 6	1838878	1931594	1891225	1744099	1826544	1765532

Table M4-3 Comparison of Peak Area Per Unit Mass Under Different Extraction Methods and Extraction Durations.

	Ultrasound 20 min	Ultrasound 30 min	Ultrasound 45 min	Reflux 30 min	Reflux 45 min	Reflux 60 min
Peak 1	7944031	8038177	7941885	7702291	8043158	7822949
Peak 2	1677887	1691849	1675521	1621860	1702536	1662621

Peak 3	1283505	1298884	1279930	1241233	1294312	1255930
Peak 4	826381	837702	823752	800740	846775	824906
Peak 5	1592042	1615278	1592769	1548759	1610069	1566059
Peak 6	3555449	3593663	3554934	3450928	3591317	3510702

4.2.3 Conclusion

The ultrasonic extraction with an extraction duration of 30 min is selected.

4.3 Sample mass optimization

4.3.1 Method

Based on the extraction time determined above, this study investigates the extraction differences under varying solid-to-liquid ratios and selects the optimal sample mass. The tested sample masses are 0.1 g, 0.2 g, 0.3 g, 0.4 g, and 0.5 g respectively. An appropriate sample mass is selected with the number of chromatographic peaks, resolution, and the unit mass peak area of the main chromatographic peaks as the primary evaluation indicators.

Accurately weigh an appropriate amount of *S. chinensis* powder (passed through a No. 4 sieve), place it in a stoppered conical flask, precisely add 50 mL of 80% methanol, seal the flask with the stopper, weigh the mixture, perform ultrasonic extraction for 30 min, allow the mixture to cool to room temperature, weigh it again, compensate for the mass loss with the corresponding solvent, shake the mixture evenly, filter it, collect the subsequent filtrate, pass the filtrate through a 0.45 μm membrane filter, and the test solution is obtained.

4.3.2 Experimental Result

According to data analysis, the extraction is more complete when the sample mass is 0.2 g. Therefore, 0.2 g is selected as the optimal sampling mass.

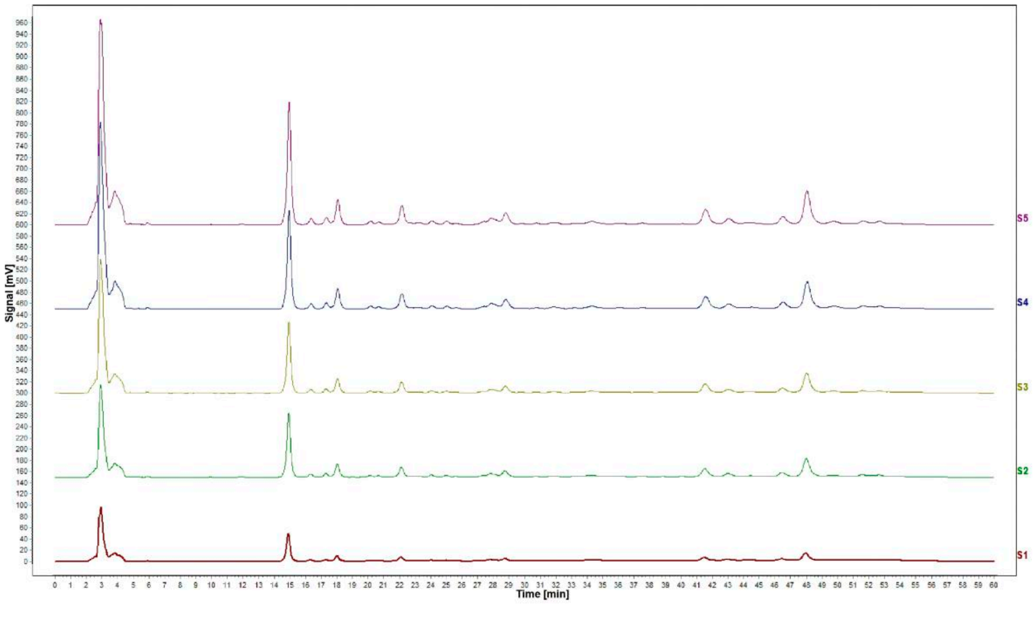

Figure M4-3 Chromatograms for investigation of different sample masses. (S1–S5 correspond to 0.1 g, 0.2 g, 0.3 g, 0.4 g and 0.5 g, respectively)

Table M4-4 Comparison of Peak Area at Different Solid-Liquid Ratios.

	0.1 g	0.2 g	0.3 g	0.4 g	0.5 g
Peak 1	894352	2078849	2301060	3198771	3967051

Peak 2	177188	414477	460080	639691	795139
Peak 3	135852	331530	365964	507680	633001
Peak 4	86720	204843	226010	312448	391537
Peak 5	153823	417277	455458	633591	789998
Peak 6	390980	959111	1040454	1432189	1780968

Table M4-5 Comparison of Peak Area Per Unit Mass at Different Solid-to-Liquid Ratios.

	0.1 g	0.2 g	0.3 g	0.4 g	0.5 g
Peak 1	7282997	9259906	7522262	7639768	7793813
Peak 2	1442899	1846223	1504021	1527803	1562159
Peak 3	1106287	1476748	1196352	1212515	1243617
Peak 4	706189	912441	738836	746234	769228
Peak 5	1252630	1858695	1488911	1513234	1552059
Peak 6	3183876	4272209	3401288	3420561	3498955

4.3.2 Conclusion

The sample mass selected is 0.2 g.

4.4 Summary

The preparation process of the test solution is as follows: take approximately 0.2 g of *S. chinensis* powder (passed through No. 4 sieve), weigh it accurately, place it in a conical flask with a stopper, add precisely 50 mL of 80% methanol, seal the flask with the stopper, weigh it, then perform ultrasonic extraction (250 W, 40 KHz) for 30 min. After cooling to room temperature, weigh it again, replenish the weight loss with 80% methanol, shake well, filter, collect the subsequent filtrate, pass it through a 0.45 μm filter membrane. The test solution is thus obtained.

5 Identification of Characteristic Chromatograms

5.1 Integrity of Characteristic Chromatograms

Record chromatograms over a duration of at least twice the measurement time specified for the fingerprint, and investigate whether all major components contained in the sample are presented in the chromatogram, as well as whether the principle of maximizing effective information is satisfied. The experimental results are presented in Figure M5-1. The results demonstrate that all peak information is completely acquired within 120 min.

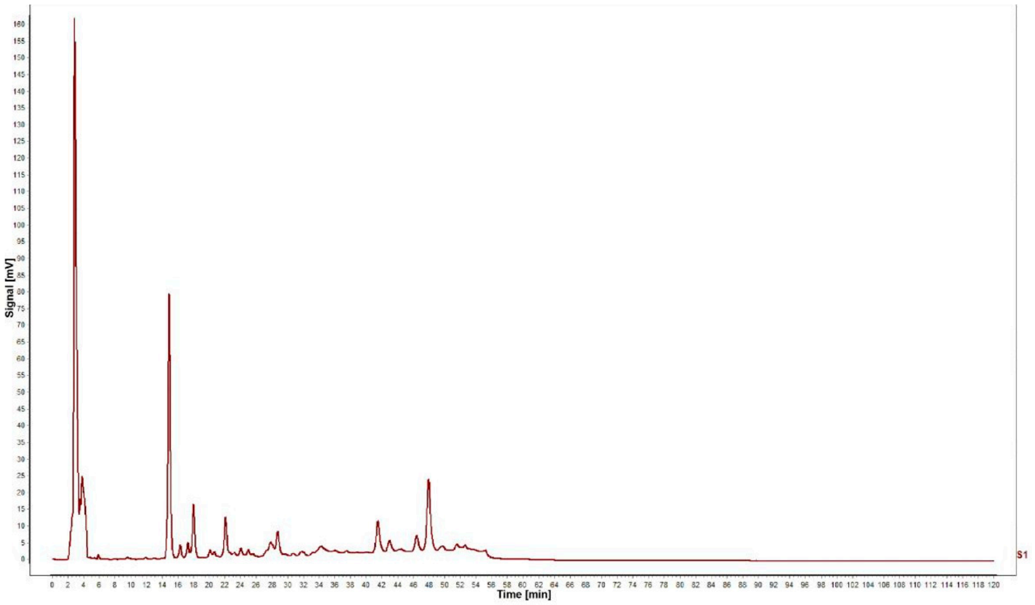

Figure M5-1 Chromatogram of Test Sample for Integrity Test.

5.2 Identification of Major Chromatographic Peaks

Each reference solution prepared following the procedure described in Section 3.3 was injected into the high-performance liquid chromatography system, and the identification results of major chromatographic peaks were recorded.

The results showed that Schisandrol A, Gomisin A, Angeloylgomisin H, Schisantherin B, Schizandrin A and Schizandrin B could be identified via the standard retention time method.

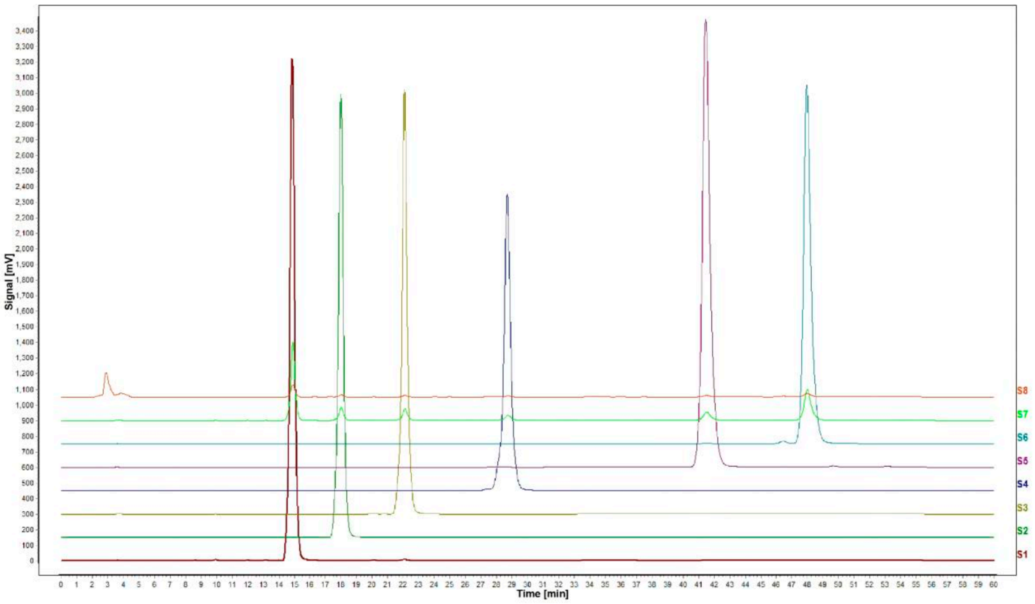

Figure M5-2 Identification of main chromatographic peaks - Chromatograms of reference substances and test sample. (S1-S8 correspond to Schisandrol A, Gomisin A, Angeloylgomisin H, Schisantherin B, Schizandrin A and Schizandrin B, mixed reference substance and SC-HL-36, respectively)

5.3 System Suitability Test

5.3.1 Methods

The determination was performed in accordance with High Performance Liquid Chromatography (General Chapter 0512).

Chromatographic Conditions and System Suitability Test: Octadecylsilane-bonded silica gel is used as the packing agent; acetonitrile is used as mobile phase A, and 0.1% phosphoric acid is used as mobile phase B, and gradient elution is performed according to the procedure specified in the following table; the detection wavelength is 220 nm, the column temperature is 30°C, and the flow rate is 0.8 mL/min. The number of theoretical plates calculated on the basis of the schisandrin peak shall not be less than 2000.

Table M5-1 Gradient Elution Procedure.

Time/min	Mobile Phase A/%	Mobile Phase B/%
0	45	55
20	60	40
26	60	40
50	75	25
51	45	55
60	45	55

Preparation of Reference Solution Accurately weigh approximately 0.2 g of *S. chinensis* powder reference medicinal material, place it in a conical flask with a stopper, precisely add 50 mL of 80% methanol, seal the flask, weigh it, and perform ultrasonic extraction (250 W, 40 KHz) for 30 min. After cooling to room temperature, reweigh the mixture, replenish the weight loss with 80% methanol, shake well, filter, take the subsequent filtrate and pass it through a 0.45 μm filter membrane to obtain the reference medicinal material reference solution. In addition, take appropriate amounts of Schisandrol A, Gomisin A, Angeloylgomisin H, Schisantherin B, Schizandrin A and Schizandrin B reference substances, add methanol to prepare a mixed reference solution containing 40 μg of Schisandrol A, 10 μg each of Gomisin A, Angeloylgomisin H, Schisantherin B and Schizandrin A, and 20 μg of Schizandrin B per 1 mL, which is used as the mixed reference substance solution.

Preparation of Test Solution: Take approximately 0.2 g of *S. chinensis* powder (sieved through No. 4 sieve), weigh it precisely, place it in a conical flask with a stopper, add precisely 50 mL of 80% methanol, seal the stopper, weigh the total mass, perform ultrasonic treatment (250 W, 40 KHz) for 30 min, allow it to cool to room temperature, weigh the total mass again, compensate for the lost mass with 80% methanol, shake well, filter, and take the subsequent filtrate to obtain the test solution.

Assay Method: Precisely aspirate 10 μL of the reference solution and 10 μL of the test solution respectively, inject them into the high-performance liquid chromatograph, and conduct the determination to complete the procedure.

5.3.2 Results

When samples were injected using this method, the peaks of both the reference substance and the test sample showed symmetrical shapes with good resolution. The test sample solution exhibited a chromatographic peak at the retention time corresponding to the reference substance.

Sample injection was performed following the procedure specified in Section 4.3.1. The peak shapes of both the reference substance and test sample were symmetrical, with satisfactory resolution. The test solution exhibited corresponding chromatographic peaks at retention times consistent with those of the standard substances Schisandrol A, Gomisin A, Angeloylgomisin H, Schisantherin B, Schizandrin A and Schizandrin B. The

theoretical plate number calculated based on the Schisandrol A peak was not less than 2000.

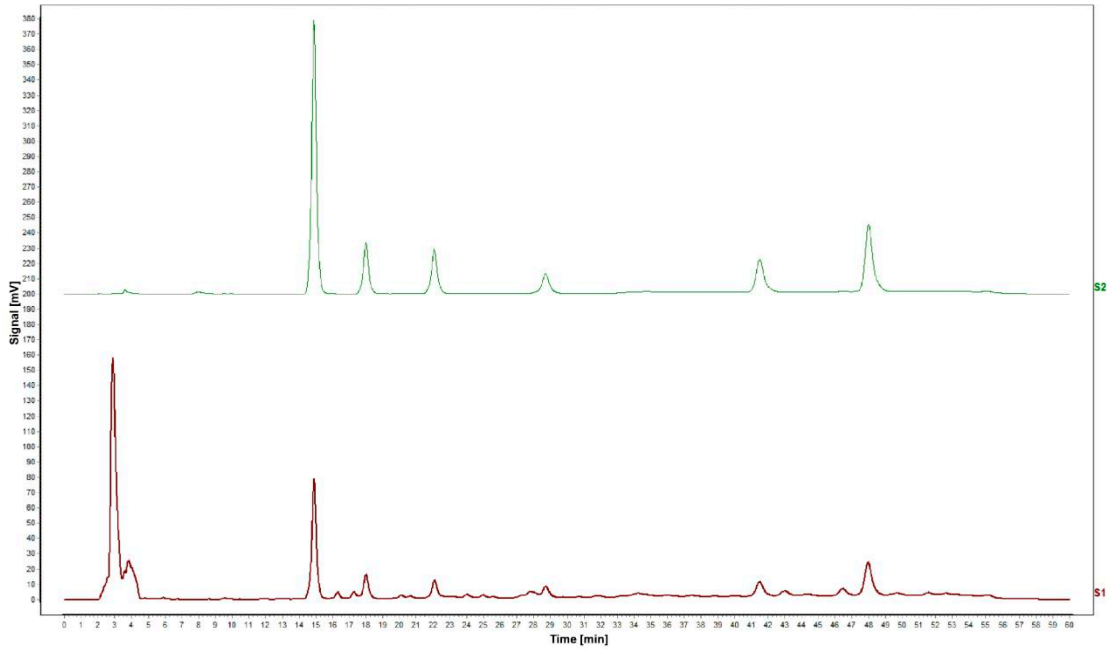

Figure M5-3 System Suitability Test. (S1: SC-HL-36, S2: mixed standard solution)

6 Construction of Reference Chromatogram

6.1 Establishment of Reference Characteristic Chromatogram and Calibration of Common Peaks

In accordance with the requirements specified in National Drug Standard Work Manual (4th Edition), samples involved in the construction of reference spectra shall possess appropriate representativeness. Therefore, 15 batches of the Chinese medicinal material *S. chinensis* (sample information is detailed in Table M6-1) were selected for injection under the chromatographic conditions and treatment procedures specified in Section 8.

Table M6-1 Information of 15 batches of *S. chinensis* samples.

Serial Number	Species	Batch Number	Place of Origin
1	SC	SC-TH-57	TaHe
2	SC	SC-HM-58	HuMa
3	SC	SC-JGDQ-59	JiaGeDaQi
4	SC	SC-TH-20	TongHe
5	SC	SC-BY-23	BaYan
6	SC	SC-AH-49	AiHui
7	SC	SC-SW-51	SunWu
8	SC	SC-WDLC-55	WuDaLianChi
9	SC	SC-BA-56	BeiAn
10	SC	SC-HS-27	HenShan
11	SC	SC-WMH-4	WuMaHe
12	SC	SC-TL-6	TieLi

Serial Number	Species	Batch Number	Place of Origin
13	SC	SC-TW-7	TangWang
14	SC	SC-NC-10	NanCha
15	SC	SC-JL-13	JinLin

The test sample solution was subjected to instrumental measurement in accordance with the preparation method and chromatographic conditions. Using the Similarity Evaluation System for Chromatographic Fingerprint of Traditional Chinese Medicine (2012 version) with a time window of 0.5, we generated the reference chromatogram by the average method and performed full-spectrum peak matching.

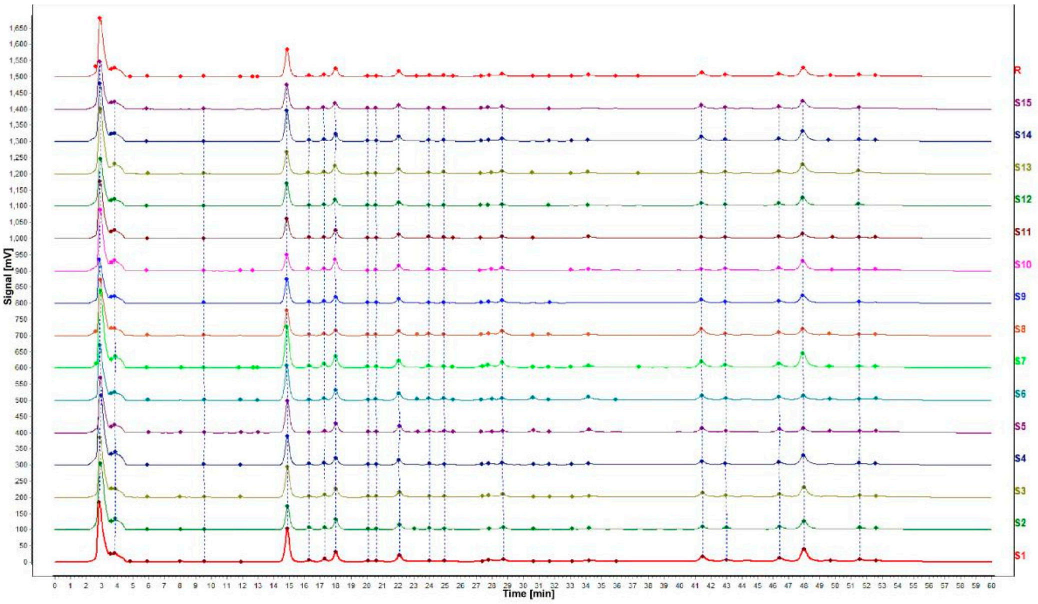

Figure M6-1 Full-spectrum peak matching and fitting results of chromatograms for 15 batches of test samples. (S1-S15 correspond to the chromatograms of test samples listed in Table M6-1, and R is the reference characteristic chromatogram automatically generated by the system via fitting of the 15 batches of test samples mentioned above)

6.2 Fitting of Reference Chromatograms

Based on the chromatographic profiles of each sample, in addition to the 6 components (Schisandrol A, Gomisin A, Angeloylgomisin H, Schisantherin B, Schizandrin A and Schizandrin B) that have been assigned via reference standards, 3 additional chromatographic peaks with satisfactory resolution, large peak area and stable retention were selected as characteristic peaks, which were identified by calculating the relative retention time of each characteristic peak with respect to the peak of Schisandrol A. In the chromatograms of 15 batches of *S. chinensis* test samples, all peaks correspond in retention time to those in the chromatogram of the reference medicinal material, among which 5 peaks match the retention time of the corresponding reference substance peaks, respectively. The retention time of each characteristic peak and the relative retention time data with respect to the Schisandrol A peak (S peak) are shown in the following table.

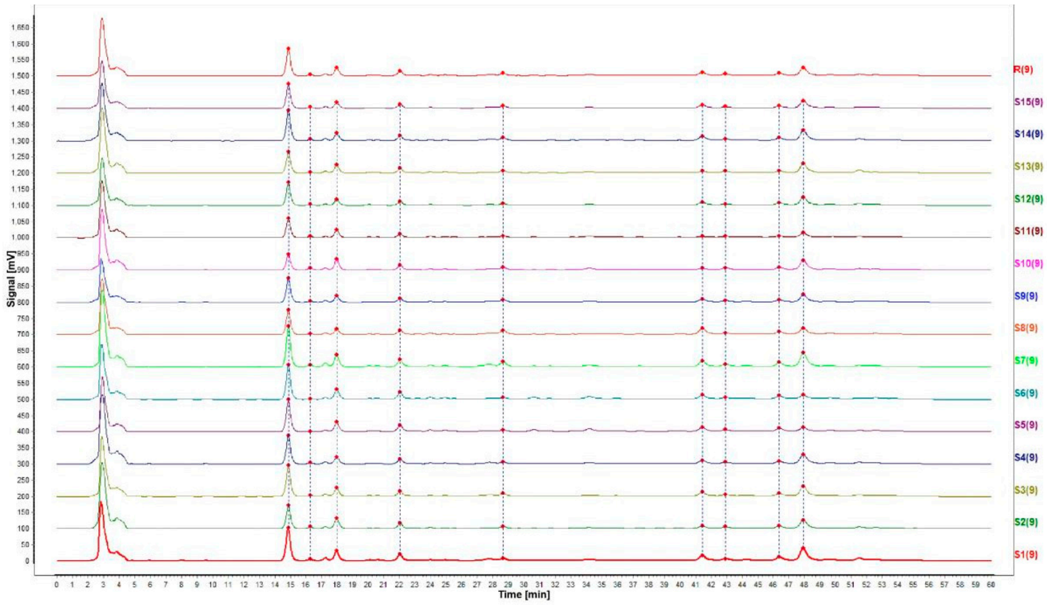

Figure M6-2 Reference characteristic chromatogram before fitting. (S1-S15 correspond to Table M6-1 respectively, R is the reference characteristic chromatogram of the 15 batches of test samples automatically fitted by the system)

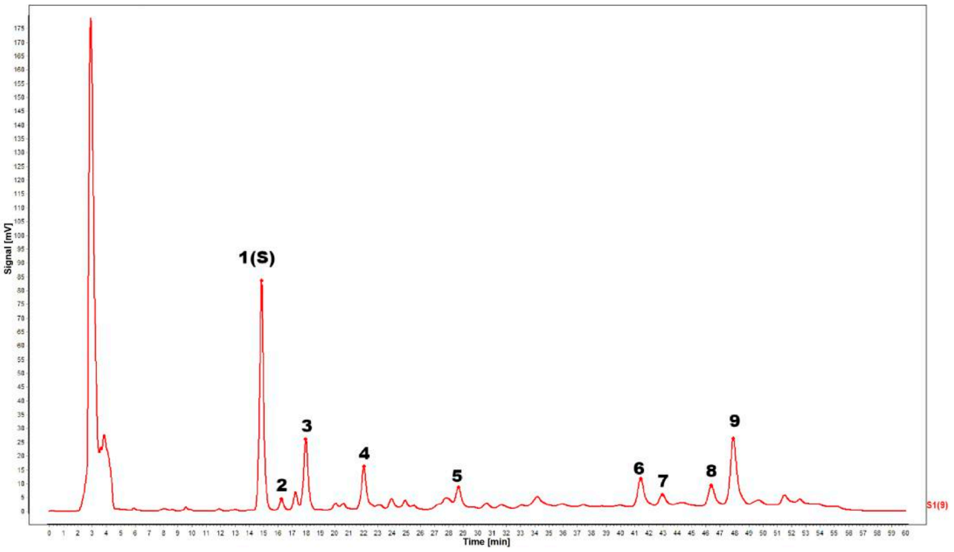

Figure M6-3 Fitted control characteristic chromatogram. (Peak 1 (S): Schisandrol A; Peak 3: Gomisin A; Peak 4: Angeloylgomisin H; Peak 5: Schisantherin B; Peak 6: Schisandrin A; Peak 9: Schisandrin B; Column: Agilent 5HC-C18 (2), 4.6 mm × 250 mm, 5 μm)

Table M6-2 Retention times of each characteristic peak for 15 batches of test samples.

Sample Batch Number	Peak 1(S)	Peak 2	Peak 3	Peak 4	Peak 5	Peak 6	Peak 7	Peak 8	Peak 9
Reference Medicinal Material	14.879	16.283	17.981	22.061	28.7	41.483	42.985	46.433	47.976
SC-TH-57	14.896	16.303	18.001	22.074	28.715	41.492	42.997	46.439	47.981
SC-HM-58	14.902	16.307	18.002	22.071	28.71	41.484	43.001	46.432	47.976
SC-JGDQ-59	14.896	16.3	17.997	22.069	28.699	41.477	42.982	46.42	47.962
SC-TH-20	14.879	16.282	17.979	22.047	28.673	41.441	42.937	46.388	47.933
SC-BY-23	14.891	16.304	17.997	22.071	28.694	41.455	42.978	46.398	47.94
SC-AH-49	14.862	16.274	17.97	22.038	28.668	41.437	42.962	46.386	47.93
SC-SW-51	14.867	16.27	17.965	22.03	28.651	41.418	42.942	46.369	47.914
SC-WDLC-55	14.867	16.272	17.967	22.034	28.65	41.42	42.932	46.375	47.912
SC-BA-56	14.863	16.268	17.965	22.038	28.658	41.419	42.924	46.366	47.911
SC-HS-27	14.856	16.256	17.955	22.023	28.639	41.415	42.932	46.363	47.905
SC-WMH-4	14.861	16.264	17.961	22.03	28.653	41.417	42.934	46.365	47.912
SC-TL-6	14.857	16.259	17.957	22.026	28.643	41.412	42.924	46.363	47.903
SC-TW-7	14.853	16.255	17.951	22.02	28.637	41.407	42.918	46.356	47.903
SC-NC-10	14.862	16.266	17.961	22.034	28.658	41.423	42.934	46.374	47.916
SC-JL-13	14.842	16.245	17.943	22.017	28.644	41.422	42.931	46.377	47.923
Retention Time RSD/%	0.13	0.13	0.11	0.091	0.092	0.068	0.065	0.057	0.055

Table M6-3 Relative retention times of each characteristic peak to the S peak for 15 batches of test samples.

	Peak 1（S）	Peak 2	Peak 3	Peak 4	Peak 5	Peak 6	Peak 7	Peak 8	Peak 9
Reference Medicinal Material	1.000	1.094	1.208	1.483	1.929	2.788	2.889	3.121	3.224
SC-TH-57	1.000	1.094	1.208	1.482	1.928	2.785	2.886	3.118	3.221
SC-HM-58	1.000	1.094	1.208	1.481	1.927	2.784	2.886	3.116	3.219

SC-JGDQ-59	1.000	1.094	1.208	1.482	1.927	2.784	2.885	3.116	3.220
SC-TH-20	1.000	1.094	1.208	1.482	1.927	2.785	2.886	3.118	3.222
SC-BY-23	1.000	1.095	1.209	1.482	1.927	2.784	2.886	3.116	3.219
SC-AH-49	1.000	1.095	1.209	1.483	1.929	2.788	2.891	3.121	3.225
SC-SW-51	1.000	1.094	1.208	1.482	1.927	2.786	2.888	3.119	3.223
SC-WDLC-55	1.000	1.095	1.209	1.482	1.927	2.786	2.888	3.119	3.223
SC-BA-56	1.000	1.095	1.209	1.483	1.928	2.787	2.888	3.120	3.224
SC-HS-27	1.000	1.094	1.209	1.482	1.928	2.788	2.890	3.121	3.225
SC-WMH-4	1.000	1.094	1.209	1.482	1.928	2.787	2.889	3.120	3.224
SC-TL-6	1.000	1.094	1.209	1.483	1.928	2.787	2.889	3.121	3.224
SC-TW-7	1.000	1.094	1.209	1.483	1.928	2.788	2.890	3.121	3.225
SC-NC-10	1.000	1.094	1.209	1.483	1.928	2.787	2.889	3.120	3.224
SC-JL-13	1.000	1.095	1.209	1.483	1.930	2.791	2.893	3.125	3.229
Relative Retention Time RSD/%	0	0.045	0.041	0.042	0.045	0.070	0.077	0.078	0.083

7 Methodology Investigation

This study was conducted in accordance with regulations including Technical Requirements for Fingerprint Research of Traditional Chinese Medicine Injections (Provisional) (hereinafter referred to as "Technical Requirements"), Illustrative Examples of Technical Requirements and Establishment of Fingerprints for Chinese Medicinal Materials, National Drug Standards Work Manual and Chinese Pharmacopoeia. It is specified in the Technical Requirements that when establishing a fingerprint detection method, the corresponding detection method shall be selected based on the characteristics of the test sample and the physicochemical properties of its contained chemical components; the basis for selecting the detection method and the principle of the method shall be specified; and the methodological investigation data and relevant fingerprints (including stability, precision and reproducibility) of the detection method shall be determined.

Stability Test: This procedure mainly investigates the stability of the test solution. The same test solution is sampled and analyzed at different time points to examine the consistency of the relative retention time and peak area ratio of chromatographic peaks, so as to determine the appropriate detection time.

Precision Test: The test primarily examines the precision of the instrument. The same test sample is selected for more than 5 consecutive injections to investigate the consistency of the relative retention time and peak area ratio of chromatographic peaks. For fingerprint spectra established via high-performance liquid chromatography and gas chromatography, the relative standard deviation (RSD) of the peak area ratio of each chromatographic peak with a specified common peak area ratio in the fingerprint spectrum shall not exceed 3%, while for other methods, the RSD shall not exceed 5%.

Reproducibility Test: This test is primarily designed to evaluate the reproducibility of the experimental procedure. No less than 5 test samples from the same batch are prepared and tested in accordance with the established sample preparation and detection methods, to assess the consistency of the relative retention time and peak area ratio of chromatographic peaks. For fingerprint spectra established via high-performance liquid chromatography and gas chromatography, the relative standard deviation (RSD) of the peak area ratio of all common peaks specified in the fingerprint spectrum shall not exceed 3%; for other methods, the RSD shall not exceed 5%.

To verify the reliability of the analytical method, the test sample with batch number SC-HL-36 was used to conduct methodological investigation on the following four indicators. Since the method established in this study is a characteristic chromatogram method, and the results are determined only by the relative retention time of each characteristic peak to the S peak, only the differences in relative retention time are presented in the methodological investigation, and no specific requirements are imposed on the peak area ratio.

7.1 Stability Test

This test mainly investigates the stability of the test solution. The same test sample was collected and detected at 0 h, 6 h, 12 h, 18 h and 24 h respectively, to examine the consistency of the relative retention time of chromatographic peaks. The retention time of each characteristic peak and the relative retention time relative to the S peak are shown in the table below. The relative standard deviation (RSD) of all relative retention time values is less than 2%, which confirms that the sample remains stable within 24 hours.

Table M7-1 Stability Test Data.

Time		Peak 1(S)	Peak 2	Peak 3	Peak 4	Peak 5	Peak 6	Peak 7	Peak 8	Peak 9
0 h	Retention Time	14.949	16.363	18.069	22.153	28.818	41.614	43.103	46.560	48.099
	Relative Retention Time	1.00	1.09	1.21	1.48	1.93	2.78	2.88	3.11	3.22
6 h	Retention Time	14.904	16.314	18.013	22.081	28.720	41.527	43.024	46.479	48.017
	Relative Retention Time	1.00	1.09	1.21	1.48	1.93	2.79	2.89	3.12	3.22
12 h	Retention Time	14.879	16.286	17.984	22.049	28.670	41.460	42.963	46.411	47.949
	Relative Retention Time	1.00	1.09	1.21	1.48	1.93	2.79	2.89	3.12	3.22
18 h	Retention Time	14.859	16.264	17.961	22.020	28.635	41.414	42.917	46.365	47.906
	Relative Retention Time	1.00	1.09	1.21	1.48	1.93	2.79	2.89	3.12	3.22
24 h	Retention Time	14.889	16.296	17.994	22.058	28.678	41.464	42.968	46.416	47.957
	Relative Retention Time	1.00	1.09	1.21	1.48	1.93	2.78	2.89	3.12	3.22
Relative Retention Time RSD/%		0	0	0	0	0	0.20	0.16	0.15	0

7.2 Precision Test

This test mainly investigates the precision of the instrument. The same test sample was injected continuously for 6 times to examine the consistency of the relative retention time of chromatographic peaks. The retention time of each characteristic peak and the relative retention time data relative to the S peak are shown in the table below, and the RSD of all relative retention times is less than 2%.

Table M7-2 Precision Test Data.

		Peak 1(S)	Peak 2	Peak 3	Peak 4	Peak 5	Peak 6	Peak 7	Peak 8	Peak 9
Precision 1	Retention Time	14.856	16.255	17.954	22.030	28.653	41.440	42.939	46.393	47.936
	Relative Retention Time	1.00	1.09	1.21	1.48	1.93	2.79	2.89	3.12	3.23
Precision 2	Retention Time	14.885	16.287	17.990	22.073	28.705	41.488	42.982	46.434	47.974
	Relative Retention Time	1.00	1.09	1.21	1.48	1.93	2.79	2.89	3.12	3.22
Precision 3	Retention Time	14.904	16.309	18.011	22.090	28.724	41.521	43.017	46.466	48.007
	Relative Retention Time	1.00	1.09	1.21	1.48	1.93	2.79	2.89	3.12	3.22
Precision 4	Retention Time	14.892	16.294	17.992	22.072	28.695	41.481	42.980	46.426	47.967
	Relative Retention Time	1.00	1.09	1.21	1.48	1.93	2.79	2.89	3.12	3.22
Precision 5	Retention Time	14.883	16.287	17.989	22.071	28.695	41.479	42.979	46.429	47.967
	Relative Retention Time	1.00	1.09	1.21	1.48	1.93	2.79	2.89	3.12	3.22
Precision 6	Retention Time	14.887	16.291	17.993	22.069	28.702	41.492	42.992	46.445	47.985
	Relative Retention Time	1.00	1.09	1.21	1.48	1.93	2.79	2.89	3.12	3.22
Relative Retention Time RSD/%		0	0	0	0	0	0	0	0	0.13

7.3 Reproducibility Test

This test is primarily designed to evaluate the reproducibility of the experimental method. Six samples of the test product from the same batch were prepared and injected under the chromatographic conditions and treatment procedures specified in Section 6. The consistency of the relative retention times of chromatographic peaks was investigated. The retention time of each characteristic peak and the relative retention time relative to the S peak are summarized in the table below, and the relative standard deviation (RSD) of all relative retention times is less than 2%.

Table M7-3 Reproducibility Test Data.

		Peak 1(S)	Peak 2	Peak 3	Peak 4	Peak 5	Peak 6	Peak 7	Peak 8	Peak 9
Reproducibility 1	Retention Time	14.886	16.288	17.990	22.056	28.684	41.484	42.982	46.437	47.983
	Relative Retention Time	1.00	1.09	1.21	1.48	1.93	2.79	2.89	3.12	3.22
Reproducibility 2	Retention Time	14.889	16.293	17.994	22.063	28.697	41.496	42.991	46.444	47.988
	Relative Retention Time	1.00	1.09	1.21	1.48	1.93	2.79	2.89	3.12	3.22
Reproducibility 3	Retention Time	14.884	16.285	17.987	22.053	28.684	41.480	42.973	46.424	47.971
	Relative Retention Time	1.00	1.09	1.21	1.48	1.93	2.79	2.89	3.12	3.22
Reproducibility 4	Retention Time	14.889	16.292	17.994	22.061	28.689	41.486	42.982	46.435	47.977
	Relative Retention Time	1.00	1.09	1.21	1.48	1.93	2.79	2.89	3.12	3.22
Reproducibility 5	Retention Time	14.882	16.285	17.987	22.054	28.686	41.491	42.988	46.445	47.992
	Relative Retention Time	1.00	1.09	1.21	1.48	1.93	2.79	2.89	3.12	3.22
Reproducibility 6	Retention Time	14.898	16.305	18.006	22.072	28.713	41.510	43.004	46.456	47.999
	Relative Retention Time	1.00	1.09	1.21	1.48	1.93	2.79	2.89	3.12	3.22
Relative Retention Time RSD/%		0	0	0	0	0	0	0	0	0

7.4 Durability Test of Chromatographic Conditions and Results

This study investigated the variations in relative retention time and relative peak area of each characteristic peak under small-range fluctuations of detection wavelength (± 2 nm), small-range fluctuations of column temperature (± 2℃), small-range fluctuations of flow rate (± 0.1 mL/min), and when different chromatographic columns are replaced. Due to the complex components of the test sample and frequent gradient elution changes, variations in temperature, flow rate and wavelength can lead to differences in separation performance, thus the control of temperature, flow rate and wavelength should be emphasized. The variations in relative retention time and relative peak area of each characteristic peak were also investigated when different chromatographic columns were used. The results show that different chromatographic columns exhibit differences in both the response and separation performance for each component in the test sample, and the replacement of chromatographic columns affects the number of peaks observed. Therefore, it is recommended that the Agilent 5HC-C18 (2) (4.6 × 250 mm, 5 μm) be fixed for this analysis. Given the specificity of the test sample and chromatographic conditions in this study, it is recommended that relative retention time be adopted as the identification criterion for the characteristic chromatogram, and the control of relative peak area ratio is not required.

Table M7-5 Durability Test Data. (Under Different Wavelengths)

Wavelength/nm		Peak 1(S)	Peak 2	Peak 3	Peak 4	Peak 5	Peak 6	Peak 7	Peak 8	Peak 9
218	Retention Time	14.934	16.336	18.032	22.091	28.714	41.471	42.976	46.428	47.965
	Relative Retention Time	1.00	1.09	1.21	1.48	1.92	2.78	2.88	3.11	3.21
220	Retention Time	14.951	16.357	18.056	22.116	28.731	41.489	42.996	46.439	47.979
	Relative Retention Time	1.00	1.09	1.21	1.48	1.92	2.78	2.88	3.11	3.21
222	Retention Time	14.893	16.293	17.985	22.037	28.640	41.382	42.898	46.335	47.877
	Relative Retention Time	1.00	1.09	1.21	1.48	1.92	2.78	2.88	3.11	3.21
Relative Retention Time RSD/%		0	0	0	0	0	0	0	0	0

Table M7-6 Durability Test Data. (Under Different Flow Rates)

Flow Rates(mL/min)		Peak 1(S)	Peak 2	Peak 3	Peak 4	Peak 5	Peak 6	Peak 7	Peak 8	Peak 9
0.7	Retention Time	16.517	17.970	19.783	24.018	31.287	44.400	46.117	49.336	50.897
	Relative Retention Time	1.00	1.09	1.20	1.45	1.89	2.69	2.79	2.99	3.08
0.8	Retention Time	14.951	16.357	18.056	22.116	28.731	41.489	42.996	46.439	47.979
	Relative Retention Time	1.00	1.09	1.21	1.48	1.92	2.78	2.89	3.11	3.21
0.9	Retention Time	13.465	14.810	16.404	20.297	26.424	38.674	40.033	43.639	45.158
	Relative Retention Time	1.00	1.10	1.22	1.51	1.96	2.87	2.97	3.24	3.35
Relative Retention Time RSD/%		0	0.53	0.83	2.1	1.9	3.3	3.2	4.1	4.3

Table M7-7 Durability Test Data. (at Different Column Temperatures)

Column Temperature/℃		Peak 1(S)	Peak 2	Peak 3	Peak 4	Peak 5	Peak 6	Peak 7	Peak 8	Peak 9
28	Retention Time	14.802	16.278	17.969	21.993	28.758	41.606	43.285	46.661	48.234
	Relative Retention Time	1.00	1.10	1.21	1.49	1.94	2.81	2.92	3.15	3.26
30	Retention Time	14.951	16.357	18.056	22.116	28.731	41.489	42.996	46.439	47.979
	Relative Retention Time	1.00	1.09	1.21	1.48	1.92	2.77	2.88	3.11	3.21
32	Retention Time	14.755	16.062	17.760	21.842	28.221	40.846	42.221	45.705	47.217
	Relative Retention Time	1.00	1.09	1.20	1.48	1.91	2.77	2.86	3.10	3.20
Relative Retention Time RSD/%		0	0.53	0.48	0.39	0.80	0.83	1.1	0.85	1.0

Table M7-8 Durability Test Data. (Different Chromatographic Columns)

Chromatography Column Manufacturer		Peak 1(S)	Peak 2	Peak 3	Peak 4	Peak 5	Peak 6	Peak 7	Peak 8	Peak 9
Agilent	Retention Time	14.951	16.357	18.056	22.116	28.731	41.489	42.996	46.439	47.979
	Relative Retention Time	1.00	1.09	1.21	1.48	1.92	2.77	2.88	3.11	3.21
Waters	Retention Time	16.126	17.566	19.271	23.638	31.034	43.466	/	47.883	49.245
	Relative Retention Time	1.00	1.09	1.20	1.47	1.92	2.70	/	2.97	3.05
Shimadzu	Retention Time	16.541	17.756	20.055	24.062	31.418	44.372	45.612	49.405	50.726
	Relative Retention Time	1.00	1.07	1.21	1.45	1.90	2.68	2.76	2.99	3.07
Relative Retention Time RSD/%		0	1.1	0.48	1.1	0.61	1.8	/	2.6	2.9

Note: When using a Waters chromatography column, Peak 7 could not be automatically integrated, and therefore the data entry is marked with "/" in the table.

8 Draft Quality Standard

The determination was performed in accordance with High Performance Liquid Chromatography (General Chapter 0512).

Chromatographic Conditions and System Suitability Test: Octadecylsilane chemically bonded silica shall be used as the stationary phase filler; acetonitrile is employed as mobile phase A, and 0.1% phosphoric acid is employed as mobile phase B. Gradient elution shall be performed in accordance with the parameters specified in the following table; the detection wavelength is 220 nm, the column temperature is 30 ℃, and the flow rate is 0.8 mL/min. The number of theoretical plates calculated based on the Schisandrol A peak shall be no less than 2000.

Table M8-1 Gradient Elution Table of Mobile Phase.

Time/min	Mobile Phase A/%	Mobile Phase B/%
0	45	55
20	60	40
26	60	40
50	75	25
51	45	55
60	45	55

Preparation of Reference Solution: Accurately weigh approximately 0.2 g of *S. chinensis* reference medicinal material, place it in a conical flask with a stopper, add precisely 50 mL of 80% methanol, seal the flask with the stopper, weigh the system, perform ultrasonic extraction (250 W, 40 KHz) for 30 min, allow it to cool to room temperature, reweigh the system, replenish the weight loss with 80% methanol, shake well, filter, take the subsequent filtrate and pass it through a 0.45 μm filter membrane, and the resulting filtrate is used as the reference medicinal material reference solution. Separately, take appropriate amounts of Schisandrol A, Gomisin A, Angeloylgomisin H, Schisantherin B, Schizandrin A and Schizandrin B reference substances, add methanol to prepare a mixed reference solution containing 40 μg of Schisandrol A, 10 μg of Gomisin A, 10 μg of Angeloylgomisin H, 10 μg of Schisantherin B, 10 μg of Schizandrin A and 20 μg of Schizandrin B per 1 mL, which is used as the mixed reference substance reference solution.

Preparation of Test Solution: Take approximately 0.2 g of *S. chinensis* powder (sieved through No. 4 sieve), weigh it accurately, place it in a conical flask with a stopper, add precisely 50 mL of 80% methanol, stopper the flask tightly, weigh and record the total weight. Perform ultrasonic treatment (250 W, 40 KHz) for 30 minutes, allow the solution to cool to room temperature, weigh and record the total weight again, compensate for the lost weight with 80% methanol, shake well, filter, and take the subsequent filtrate to obtain the test solution.

Mensuration: Accurately aspirate 10 μL each of the reference solution and the test solution separately, inject into the liquid chromatograph, perform the measurement, and complete the assay.

Standard Determination: The characteristic chromatogram of the test solution shall contain 9 characteristic peaks, which correspond to the 9 characteristic peaks in the chromatogram of the reference medicinal material. Among them, the retention times of Peak 1, Peak 3, Peak 4, Peak 5, Peak 6, and Peak 9 shall be consistent with those of the reference substance peaks of Schisandrol A, Gomisin A, Angeloylgomisin H, Schisantherin B, Schizandrin A and Schizandrin B, respectively. Use the peak corresponding to the Schisandrol A reference peak as the S peak, calculate the relative retention times of each characteristic peak with respect to the S peak; the calculated values shall be within ±10% of the specified values. The specified retention time values are: 1.00 [Peak 1 (S)], 1.09 (Peak 2), 1.21 (Peak 3), 1.48 (Peak 4), 1.92 (Peak 5), 2.78 (Peak 6), 2.89 (Peak 7), 3.12 (Peak 8), 3.22

(Peak 9).

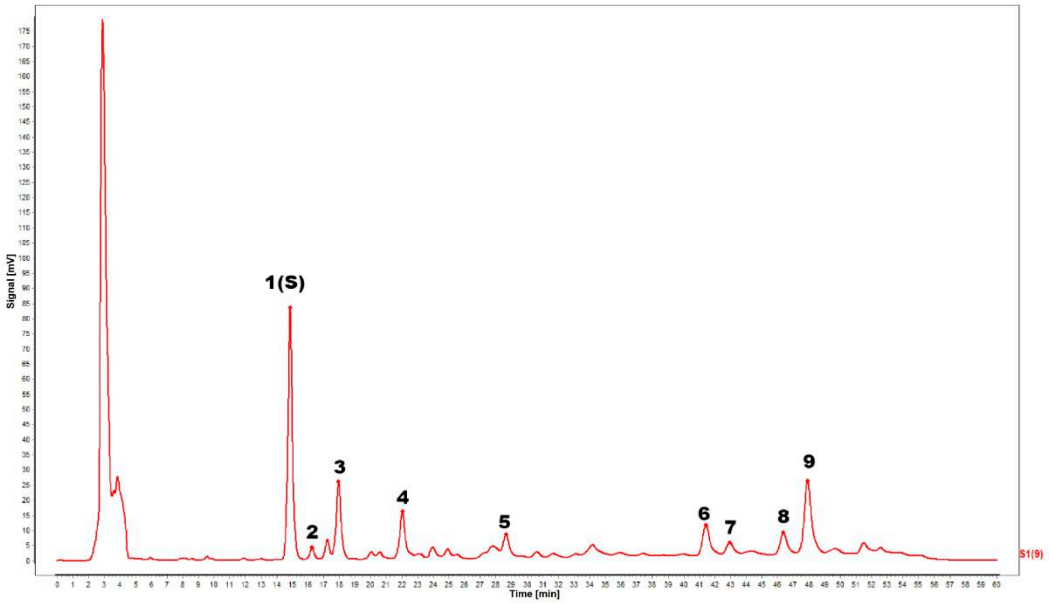

Figure M8-1 Reference chromatogram. (Peak 1(S): Schisandrol A; Peak 3: Gomisin A; Peak 4: Angeloylgomisin H; Peak 5: Schisantherin B; Peak 6: Schizandrin A; Peak 9: Schizandrin B; Agilent 5HC-C18(2), 4.6 mm × 250 mm, 5 μm)

9 Test Results

A total of 46 batches of samples were processed in accordance with the specified pretreatment method and chromatographic conditions specified in Section 7, and corresponding result determinations were conducted.

9.1 Test Method

The determination was performed in accordance with High Performance Liquid Chromatography (General Chapter 0512).

The determination was performed in accordance with the sample preparation method and chromatographic conditions specified under Section 7.

9.2 Results

The relative retention times of all obtained characteristic peaks and S peak are presented in the following table.

Table M9-1 Summary of Test Results for 46 Batches of Test Samples.

	Peak 1（S）	Peak 2	Peak 3	Peak 4	Peak 5	Peak 6	Peak 7	Peak 8	Peak 9
Reference Medicinal Materials	1.000	1.094	1.208	1.483	1.929	2.788	2.889	3.121	3.224
SC-TH-57	1.000	1.094	1.208	1.482	1.928	2.785	2.886	3.118	3.221
SC-HM-58	1.000	1.094	1.208	1.481	1.927	2.784	2.886	3.116	3.219
SC-JGDQ-59	1.000	1.094	1.208	1.482	1.927	2.784	2.885	3.116	3.220
SC-TH-20	1.000	1.094	1.208	1.482	1.927	2.785	2.886	3.118	3.222
SC-BY-23	1.000	1.095	1.209	1.482	1.927	2.784	2.886	3.116	3.219
SC-WC-24	1.000	1.094	1.208	1.482	1.925	2.786	2.887	3.121	3.225
SC-AC-25	1.000	1.094	1.208	1.481	1.924	2.786	2.889	3.121	3.225
AC-YS-26	1.000	1.094	1.208	1.482	1.924	2.786	2.888	3.121	3.225
SC-FZ-28	1.000	1.094	1.208	1.481	1.923	2.785	2.887	3.119	3.224
SC-WH-30	1.000	1.094	1.208	1.482	1.924	2.786	2.888	3.121	3.225
SC-YBL-33	1.000	1.094	1.208	1.481	1.923	2.785	2.887	3.120	3.225
SC-YL-39	1.000	1.094	1.208	1.482	1.924	2.786	2.888	3.120	3.224
SC-SZ-42	1.000	1.094	1.208	1.481	1.923	2.785	2.888	3.120	3.224

SC-LB-46	1.000	1.094	1.208	1.481	1.923	2.784	2.887	3.119	3.223
SC-XK-14	1.000	1.094	1.208	1.481	1.924	2.787	2.890	3.121	3.226
SC-AH-49	1.000	1.095	1.209	1.483	1.929	2.788	2.891	3.121	3.225
SC-SW-51	1.000	1.094	1.208	1.482	1.927	2.786	2.888	3.119	3.223
SC-WDLC-55	1.000	1.095	1.209	1.482	1.927	2.786	2.888	3.119	3.223
SC-BA-56	1.000	1.095	1.209	1.483	1.928	2.787	2.888	3.120	3.224
SC-HS-27	1.000	1.094	1.209	1.482	1.928	2.788	2.890	3.121	3.225
SC-HL-36	1.000	1.094	1.208	1.479	1.922	2.775	2.876	3.106	3.209
SC-MS-43	1.000	1.094	1.208	1.481	1.923	2.785	2.887	3.119	3.224
SC-LS-60	1.000	1.094	1.208	1.481	1.924	2.787	2.890	3.123	3.227
SC-HN-22	1.000	1.094	1.209	1.482	1.926	2.789	2.891	3.124	3.228
SC-TY-38	1.000	1.094	1.208	1.481	1.922	2.779	2.880	3.112	3.216
SC-ML-34	1.000	1.094	1.208	1.481	1.923	2.782	2.883	3.114	3.218
SC-LK-37	1.000	1.094	1.208	1.480	1.921	2.781	2.882	3.115	3.218
SC-DN-40	1.000	1.094	1.208	1.481	1.923	2.780	2.882	3.113	3.216

SC-SFH-41	1.000	1.094	1.208	1.481	1.922	2.780	2.882	3.113	3.217
SC-HL-48	1.000	1.094	1.208	1.480	1.922	2.779	2.880	3.111	3.214
SC-NA-52	1.000	1.094	1.207	1.479	1.920	2.771	2.873	3.102	3.205
SC-BL-31	1.000	1.094	1.208	1.481	1.922	2.781	2.883	3.115	3.219
SC-QTH-32	1.000	1.094	1.208	1.482	1.925	2.787	2.889	3.122	3.226
SC-RH-21	1.000	1.094	1.208	1.481	1.923	2.781	2.882	3.114	3.218
SC-BQ-35	1.000	1.094	1.208	1.481	1.923	2.781	2.883	3.116	3.219
SC-QA-9	1.000	1.094	1.208	1.481	1.924	2.784	2.886	3.117	3.221
SC-BL-15	1.000	1.094	1.208	1.481	1.924	2.784	2.886	3.118	3.222
SC-SL-16	1.000	1.094	1.208	1.481	1.923	2.784	2.886	3.118	3.222
SC-YH-1	1.000	1.094	1.208	1.481	1.923	2.783	2.885	3.116	3.221
SC-WY-3	1.000	1.094	1.208	1.481	1.923	2.783	2.885	3.117	3.221
SC-WMH-4	1.000	1.094	1.209	1.482	1.928	2.787	2.889	3.120	3.224
SC-TL-6	1.000	1.094	1.209	1.483	1.928	2.787	2.889	3.121	3.224
SC-TW-7	1.000	1.094	1.209	1.483	1.928	2.788	2.890	3.121	3.225

SC-NC-10	1.000	1.094	1.209	1.483	1.928	2.787	2.889	3.120	3.224
SC-JL-13	1.000	1.095	1.209	1.483	1.930	2.791	2.893	3.125	3.229
SC-JY-19	1.000	1.094	1.208	1.482	1.925	2.786	2.889	3.120	3.224
Mean	1.00	1.09	1.21	1.48	1.92	2.78	2.89	3.12	3.22

9.3 Test Conclusion

Nine characteristic peaks shall be presented in the characteristic chromatogram of the test sample, which shall correspond to the nine characteristic peaks in the chromatogram of the reference medicinal material.. Among them, the retention times of Peak 1, Peak 3, Peak 4, Peak 5, Peak 6 and Peak 9 are consistent with those of the reference substance peaks of Schisandrol A, Gomisin A, Angeloylgomisin H, Schisantherin B, Schizandrin A and Schizandrin B, respectively.
